# Assessing the Ecotoxicological Effects of Emerging Drug and Dye Pollutants on Plant–Soil Systems Pre- and Post-Photocatalytic Wastewater Treatment

**DOI:** 10.3390/plants14243835

**Published:** 2025-12-16

**Authors:** Maria Paiu, Lidia Favier, Maria Gavrilescu

**Affiliations:** 1 Department of Environmental Engineering and Management, “Cristofor Simionescu” Faculty of Chemical Engineering and Environmental Protection, “Gheorghe Asachi” Technical University of Iasi, 700050 Iasi, Romania; maria.paiu@student.tuiasi.ro; 2 University Rennes, Ecole Nationale Supérieure de Chimie de Rennes, CNRS, ISCR—UMR6226, F-35000 Rennes, France; 3 Academy of Romanian Scientists, 3 Ilfov Street, 050044 Bucharest, Romania; 4 Academy of Technical Sciences of Romania, 26 Dacia Blvd., 010413 Bucharest, Romania

**Keywords:** emerging pollutants, environmental risk assessment, pharmaceuticals, photocatalytic degradation, soil–plant interactions, synthetic dyes, transformation products, phytotoxicity, wastewater reuse

## Abstract

Emerging pollutants such as pharmaceuticals and synthetic dyes increasingly enter agricultural soils through irrigation with treated or untreated wastewater and via biosolid amendments, raising concerns for plant health, soil functionality, and food chain safety. Their environmental behavior is governed by complex interactions between compound physicochemistry, soil properties, and plant physiology, leading to variable persistence, mobility, and ecotoxicological outcomes. This review synthesizes current evidence on the fate, uptake, and phytotoxic effects of drug and dye contaminants in plant–soil systems, and provides a comparative assessment of ecological risks before and after photocatalytic wastewater treatment. The analysis integrates findings from soil- and hydroponic-based studies addressing pollutant sorption–desorption dynamics, leaching, microbial transformations, and plant responses ranging from germination impairment and biomass reduction to oxidative stress and genotoxicity. Special emphasis is given to the formation and behavior of transformation products generated during photocatalytic degradation, which may display altered mobility or toxicity relative to parent compounds. Comparative evaluation reveals that photocatalysis substantially reduces contaminant loads and toxicity in many cases, although incomplete mineralization or the formation of reactive intermediates can sustain or enhance adverse effects under certain conditions. By linking pollutant fate mechanisms with plant and soil responses, this review highlights both the potential and the limitations of photocatalysis as a sustainable strategy for safeguarding agroecosystems in the context of expanding wastewater reuse.

## 1. Introduction

### 1.1. Context and Problem Statement: Increasing Detection of Pharmaceuticals and Dyes in Soil and Water Environments

Evidence from global monitoring campaigns shows that active pharmaceutical ingredients (APIs) are now ubiquitous in rivers worldwide, with potentially harmful levels at a substantial fraction of sampled sites [1]. Updated modeling further indicates large antibiotic loads in river networks, underscoring ongoing emissions and treatment gaps [2]. Regulatory and agency syntheses echo these findings, documenting pharmaceutical residues from surface waters into groundwater and even drinking water, driven by consumption, excretion, and incomplete removal in wastewater treatment [3]. In agroecosystems, reclaimed wastewater irrigation and soil amendments (biosolids/manure) represent major transfer pathways from water to soil and plants [4]. Large-scale field data demonstrate frequent detection of pharmaceuticals along the irrigation water–soil–crop continuum across commercial farms, with leafy vegetables showing higher burdens and composition patterns dominated by persistent compounds [5]. Recent frameworks and reviews highlight that such inputs interact with soil properties to control sorption, mobility, and root uptake, thereby shaping exposure at the plant–soil interface [6,7].

Synthetic dyes, particularly azo dyes, remain prevalent in industrial effluents and are increasingly reported in receiving waters and adjacent soils, where they can persist, hinder light penetration, and generate toxic aromatic amines [8,9]. Contemporary reviews also point to the scale of dye releases from textile processes and the ecological risks for primary producers and soil microbiota [10]. Together, these trends indicate a rising detection frequency and diversity of drug and dye pollutants in plant–soil systems, with implications for soil functioning, crop safety, and wastewater reuse policies.

### 1.2. Environmental Pathways: From Wastewater to Soil and Plant Systems via Irrigation and Sludge Application

The application of treated or untreated municipal wastewater to agricultural lands has emerged as a significant vector for the entry of pharmaceutical and dye contaminants into soil–plant systems. For example, the use of reclaimed wastewater for irrigation can inadvertently introduce a broad suite of emerging contaminants, including pharmaceuticals into agricultural soils. From there, they may then be taken up by crops and potentially transferred into the human food chain [11,12]. Additionally, land–application of biosolids or treated sewage sludge further amplifies this risk: accumulating data indicate that contaminants initially present in sewage sludge persist in soils long term and influence soil health and plant uptake dynamics [13]. Once deposited in soils via irrigation or sludge amendment, pharmaceuticals and dyes follow complex fate pathways, including sorption to soil organic matter, leaching into deeper horizons, biotransformation by microbial communities, and eventual root uptake by plants. Studies reveal that plant uptake is influenced by the physicochemical properties of the compound (e.g., pKa, log Kow, ionization state), soil characteristics (texture, organic carbon content, pH), and plant species physiology [14]. Similarly, for dyes and textile–industry effluents, the irrigation of soils with dye-contaminated water not only changes soil chemistry (e.g., salinity, heavy metal co–contaminants), but also affects plant growth and soil structure, demonstrated in tomato irrigated with multi–stage dyeing wastewater where the soil pollution load index and sodium absorption ratio were significantly elevated [15]. Cumulatively, these pathways establish a continuum from wastewater generation to terrestrial exposure of plants: (i) discharge or land-application of wastewater/sludge → (ii) contaminant ingress into soils → (iii) soil–pollutant interactions (sorption, transformation) → (iv) root uptake and plant accumulation. Recent frameworks emphasize this “source–pathway–receptor” model in agricultural contexts, highlighting that while wastewater reuse offers benefits (nutrients, water savings), it also carries emerging-contaminant risks that are still insufficiently quantified [6]. Given the increasing use of reclaimed water and biosolids in agriculture (especially in water-scarce regions), and the persistent detection of emerging pollutants in the soil–plant continuum, a clear understanding of these environmental pathways is essential for assessing plant and soil ecotoxicological risks and for designing effective remediation or treatment strategies [16].

**Figure 1:**
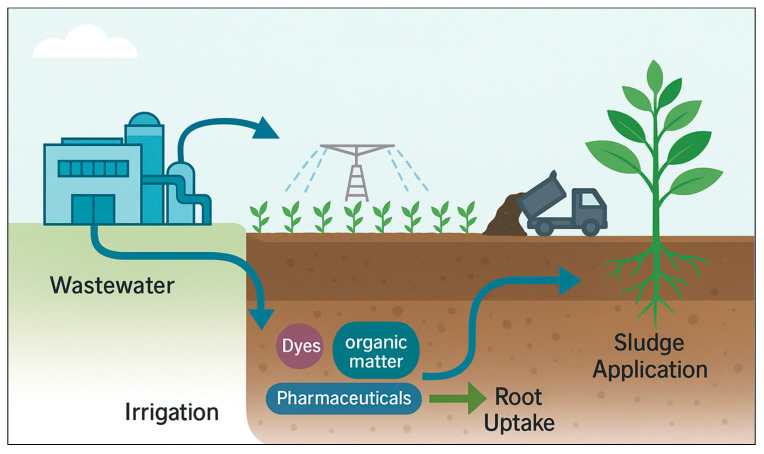
Environmental pathways for the transfer of pharmaceuticals and dyes from wastewater to soil and plant systems through irrigation and sludge application.

The fate of pharmaceuticals and dyes along the wastewater–soil–plant continuum is strongly governed by their physicochemical properties and by key soil parameters that regulate sorption and mobility (Figure 1). Compounds with higher hydrophobicity (log Kow > 3) and neutral charge exhibit stronger affinity for soil organic matter, leading to greater retention near the soil surface, while ionizable pharmaceuticals display pH-dependent partitioning regulated by their pKa values, which determines whether they occur in neutral, cationic, or anionic forms in the soil solution [6,14]. Cationic species such as fluoroquinolones interact electrostatically with clay minerals and soil colloids, whereas weak acids like diclofenac and ibuprofen become increasingly mobile under neutral to alkaline conditions due to electrostatic repulsion from negatively charged soil surfaces [17,18]. Molecular size and structural rigidity further influence diffusion into micropores and the likelihood of leaching into deeper horizons. Soil characteristics—including organic carbon content, clay mineralogy, texture, and pH—co-control these interactions: high-OC or fine-textured soils enhance sorption and reduce mobility, while sandy or low-OC soils promote desorption and vertical transport via infiltration and preferential flow pathways [15,16,17]. These coupled physicochemical and soil-driven processes collectively shape the bioavailability of pollutants in porewater, their residence time within the rhizosphere, and their subsequent uptake by roots through passive diffusion, ion trapping, or carrier-mediated transport [14]. This mechanistic understanding clarifies how pollutant molecular traits and soil properties interact to determine environmental pathways and plant exposure.

### 1.3. Relevance for Plant–Soil Health: Impacts on Soil Biota, Nutrient Cycling, and Plant Physiology

Soil microbial communities underpin decomposition, nutrient cycling, disease suppression, and plant nutrition; disturbances in their diversity and function translate directly into impaired soil fertility and crop performance [17,18,19]. Pharmaceuticals and synthetic dyes reaching soils via reclaimed irrigation water and biosolids interact with these biota in multiple ways. Antibiotics and other pharmaceutically active compounds (PhACs) can suppress key nitrogen–cycling guilds (e.g., ammonia and nitrite oxidizers), alter denitrification enzyme activities, and shift microbial community structure, with nitrification often the most sensitive process [19,20,21]. Field and mesocosm evidence further indicates that chronic inputs from treated wastewater (TWW) reshape soil bacterial assemblages and co-select for metal and antibiotic resistance, with measurable changes in soil physicochemistry over multi–year irrigation histories [21,22,23]. Recent syntheses of wastewater reuse also highlight concomitant risks: ARG dissemination, metal co-selection, and organic micropollutant carryover, alongside agronomic benefits, underscoring the need for stringent risk management [22,24,25]. For dyes, high chromophore stability and auxochrome-linked reactivity promote persistence in soils and interactions with microbial membranes and enzymes, with reported inhibition of microbial activity, disruption of C/N transformations, and broader microbiome shifts [8]. At the plant level, both dyes and PhACs can be taken up and translocated from irrigation water/soil, eliciting sub-lethal yet ecologically relevant responses: oxidative stress, perturbed photosynthetic electron transport, altered stomatal conductance, and phytohormone network imbalances, even at environmentally realistic exposures [16,23,26,27]. These stress responses intersect with nutrient acquisition (e.g., P and N), potentially depressing growth while modifying root exudation patterns that feed back on rhizosphere microbiomes [6,24,28].

**Figure 2:**
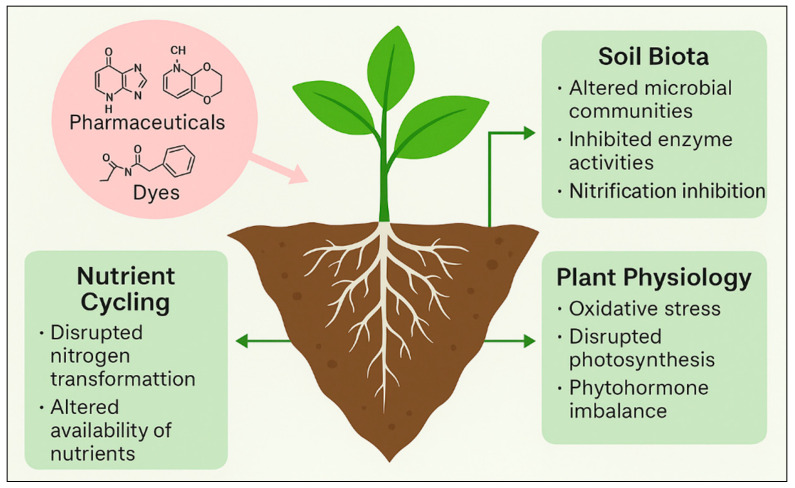
Conceptual representation of the impacts of pharmaceutical and dye pollutants on plant–soil health, emphasizing alterations in soil biota, nutrient cycling, and plant physiological responses.

The practical evidence indicates that emerging drug and dye contaminants, even after advanced treatment, can compromise soil multifunctionality and plant physiological integrity, with nitrogen cycling and redox-regulated processes among the most vulnerable nodes [8,19,20,21,23]. The interactions between pharmaceuticals, dyes, and plant–soil systems extend beyond direct chemical exposure to plants, influencing the microbial diversity, enzymatic activity, and nutrient cycling processes that sustain soil fertility. These contaminants can alter soil microbial community composition, suppress key metabolic pathways involved in nitrogen transformation, and disrupt plant physiological functions such as photosynthesis and hormonal regulation. Figure 2 conceptually illustrates these interconnected effects, highlighting how pharmaceutical and dye pollutants compromise soil biota, nutrient cycling, and plant physiological health through multiple biochemical and ecological pathways.

### 1.4. Need for Advanced Treatment: Limitations of Conventional Wastewater Systems and Role of Photocatalysis

#### 1.4.1. Challenges of Conventional Treatments and Advantages of Photocatalytic Approaches for Micropollutant Removal

Conventional wastewater treatment plants (WWTPs) were primarily designed to remove suspended solids, organic matter, and nutrients, not trace organic micropollutants such as pharmaceuticals and dyes. Consequently, a substantial fraction of these compounds and their metabolites pass through treatment processes unchanged or only partially degraded, leading to their continuous release into aquatic and terrestrial environments [25,26,29,30,31]. Even with tertiary treatments, such as chlorination, ozonation, or activated carbon adsorption, efficiency remains inconsistent, and incomplete mineralization often produces transformation products (TPs) with unknown or even enhanced toxicity [27,31,32,33]. Recent assessments of full-scale WWTPs across Europe and Asia have confirmed that removal efficiencies for pharmaceuticals rarely exceed 70–80%, and concentrations of antibiotics, anti-inflammatories, and synthetic dyes remain measurable in effluents and sludge [28,29,34,35].

The persistence of these compounds arises from their structural stability, polarity, and resistance to biodegradation, while sorption to sludge only transfers contaminants from the aqueous phase to the solid fraction, eventually returning them to soil when biosolids are reused in agriculture [30,36]. In addition, chlorination and ozonation can generate halogenated by-products, while activated carbon processes demand high energy and regeneration costs, limiting their sustainability and scalability [26,30,31,37]. These challenges underscore the need for advanced oxidation processes (AOPs) that can achieve complete mineralization rather than partial degradation.

Among AOPs, photocatalysis has emerged as a promising technology due to its ability to utilize light energy (UV or visible) to generate reactive oxygen species (ROS) such as hydroxyl and superoxide radicals capable of oxidizing recalcitrant organic compounds to CO_2_ and H_2_O [8,32,38]. Titanium dioxide (TiO_2_), zinc oxide (ZnO), and graphitic carbon nitride (g-C_3_N_4_) are the most widely investigated photocatalysts owing to their stability, non-toxicity, and reusability [32,33,38,39]. Doping and heterojunction engineering have further enhanced photocatalytic performance by extending light absorption into the visible spectrum and suppressing electron–hole recombination [8,26,30].

Photocatalysis presents several advantages as an advanced oxidation treatment, including high oxidative capacity, broad-spectrum degradation of persistent pharmaceuticals and dyes, and the possibility of operation under solar irradiation using stable and reusable catalysts such as TiO_2_, ZnO, or g-C_3_N_4_ [25,26,29,30,40]. These characteristics support efficient transformation of recalcitrant contaminants and reduction in their environmental loads prior to agricultural reuse. However, photocatalysis also exhibits limitations that require critical consideration: incomplete mineralization under suboptimal conditions, the formation of transformation products with altered toxicity, catalyst recovery requirements, and energy demands for UV-driven systems [8,32,38]. These strengths and constraints place photocatalysis among the most promising yet technically sensitive approaches in the portfolio of water treatment strategies, complementing other environmentally oriented remediation methods discussed in recent assessments, including studies on plant-based decontamination technologies.

Beyond efficient pollutant degradation, photocatalysis holds the advantage of generating fewer toxic intermediates compared with chlorination or ozonation and can be operated under solar irradiation, promoting sustainable water treatment. However, recent research emphasizes that complete detoxification is not always guaranteed, as some transformation products retain or even enhance phytotoxic potential [34,41]. Therefore, coupling photocatalytic treatment with ecotoxicological assessment, especially using plant–soil systems remains essential to ensure both removal efficiency and environmental safety.

#### 1.4.2. Operational Limitations and Engineering Advances in Photocatalytic Systems

Despite the growing adoption of photocatalysis in advanced wastewater treatment, several operational constraints continue to restrict its large-scale implementation. A primary challenge is catalyst recovery, particularly for TiO_2_, ZnO, and g-C_3_N_4_ in nanoparticulate form. Suspended catalysts provide high surface area and fast reaction kinetics but require downstream solid–liquid separation, which increases hydraulic retention time and operational cost [35,42,43,44]. Repeated use also induces surface fouling, aggregation, and partial loss of active sites, processes already documented to affect long-term photocatalyst stability and reusability. Immobilized catalysts on glass, ceramics, or carbonaceous supports facilitate recovery but often suffer from reduced active surface area and light-exposed catalytic sites [32,33,34,35,38,39,41,44].

Another intrinsic limitation is UV attenuation in large reaction volumes. Because only ~5% of the solar spectrum corresponds to UV wavelengths, penetration depths in turbid, colored, or particle-rich wastewaters remain low, reducing photon availability at catalyst surfaces. This constraint is particularly relevant for TiO_2_ and ZnO, whose wide band gaps (~3.2 eV) require UV excitation, and whose performance decreases substantially under sunlight when optical path lengths exceed a few centimeters. Reactor geometry, mixing intensity, and optical window design therefore become critical determinants of field-scale efficiency [31,33,37,39,40,45].

Recent research has focused on engineering solutions to overcome these limitations. Metal and non-metal doping (e.g., Fe, Cu, N, C, S) and the construction of heterojunction systems with semiconductors such as g-C_3_N_4_, CdS, BiVO_4_, or carbon-based materials extend absorption into the visible region and enhance charge separation. Visible-light-responsive materials address both the energy limitations of UV activation and the attenuation issues observed in deep or highly absorbing water matrices. Parallel innovations in reactor engineering, including immobilized photocatalytic membranes, fluidized-bed photoreactors, and solar concentrator systems, further aim to improve light distribution, minimize catalyst loss, and enhance process scalability [36,37,46,47].

Collectively, these advances provide pathways to overcome recovery difficulties, enhance photon utilization, and reduce energy demands, thereby supporting the transition of photocatalysis from laboratory-scale demonstrations toward robust, field-deployable water treatment technologies.

### 1.5. Scope and Objectives: Comparative Assessment of Plant–Soil Ecotoxicity Before and After Photocatalytic Degradation

This review aims to provide a broad comparative assessment of the ecotoxicological effects of pharmaceutical and dye pollutants on plant–soil systems, focusing on the differences observed before and after photocatalytic wastewater treatment. The section addresses how these emerging contaminants, when released through irrigation or sludge application, influence soil quality, microbial activity, nutrient cycling, and plant physiological performance, and how advanced photocatalytic processes alter their toxicity profiles.

The scope of this assessment extends across experimental studies conducted in soil-based systems, hydroponic setups, and controlled greenhouse or field conditions that investigate the uptake, translocation, and physiological effects of pharmaceuticals and dyes on plants. The analysis integrates information on both untreated and photocatalytically treated effluents to evaluate whether pollutant degradation leads to genuine detoxification or merely to the formation of transformation products that may still exert adverse effects on plant–soil health.

This comparative approach seeks to clarify the extent to which photocatalytic degradation mitigates or transforms ecological risks within agroecosystems. The objective is to synthesize evidence on pollutant behavior, fate, and biological effects, identifying patterns of detoxification, persistence, or residual toxicity. By contrasting pre– and post–treatment outcomes, this review contributes to understanding the environmental relevance of photocatalysis as a sustainable wastewater management strategy and provides insights into its implications for soil functionality, plant growth, and long-term ecosystem resilience.

Photocatalysis offers strong oxidation efficiency, broad-spectrum removal of persistent organic pollutants, and potential operation under solar irradiation. Its main limitations are incomplete mineralization under suboptimal conditions, formation of transformation products with residual toxicity, and catalyst recovery requirements.

### 1.6. Methodology

This review was elaborated through a systematic and integrative literature analysis combining recent peer–reviewed studies, regulatory reports, and high–impact reviews published preponderantly between 2019 and 2025. A total of approximately 320 peer-reviewed studies were examined to support this synthesis.

The methodological framework included four main stages:

1. Literature collection and selection

Publications were identified using major scientific databases (Scopus, Web of Science, ScienceDirect, MDPI, SpringerLink, and PubMed) with search terms such as pharmaceuticals, synthetic dyes, soil–plant systems, photocatalytic degradation, transformation products, and ecotoxicity.

Only articles providing quantitative or mechanistic insight into pollutant behavior in soil–plant systems before and after photocatalytic treatment were retained. Preference was given to studies with validated analytical methods (LC–MS/MS, HRMS, or GC–MS), controlled soil or hydroponic experiments, and well-defined exposure conditions.

2. Screening and categorization

The selected references were screened and categorized into thematic groups addressing:

(i) Sources and environmental entry pathways;

(ii) Pollutant fate and persistence in soils;

(iii) Plant uptake mechanisms and phytotoxic responses;

(iv) Photocatalytic degradation efficiency and transformation product (TP) formation;

(v) Post-treatment ecotoxicological outcomes.

This ensured a structured synthesis across chemical, biological, and technological dimensions.

3. Comparative and critical synthesis

Data from independent studies were compared to identify patterns of persistence, detoxification, or residual toxicity under pre– and post–photocatalytic conditions. Emphasis was placed on linking physicochemical parameters (log Kow, pKa, solubility), soil properties (pH, texture, organic matter), and plant physiological responses (biochemical markers, oxidative stress indicators) to pollutant behavior.

Case studies were integrated to illustrate real-field implications of wastewater reuse and long-term soil exposure.

4. Integration of analytical and ecological insights

The findings were synthesized into conceptual frameworks and summary tables (e.g., pollutant fate mechanisms, soil–plant interaction pathways, and monitoring indicators). This integrative approach allowed for the evaluation of the environmental relevance and safety of photocatalytic processes as sustainable treatment technologies for agricultural wastewater reuse.

To ensure the reliability of transformation product (TP) identification and quantification, this review includes largely studies that applied validated instrumental analytical techniques such as liquid chromatography coupled with tandem mass spectrometry (LC-MS/MS), high-resolution mass spectrometry (HRMS), or gas chromatography–mass spectrometry (GC–MS). These techniques offer the sensitivity and selectivity required to detect micropollutants and their transformation products in complex matrices and to distinguish between parent compounds and newly formed TPs [48,49,50]. The use of such validated methods—including appropriate quality controls, matrix-matched calibration, and stringent identification criteria—ensures that only datasets with traceable chemical confirmation are considered, while studies relying solely on bulk parameters (e.g., COD, TOC, color, absorbance) or unvalidated assays are excluded.

A quantitative synthesis of representative toxicity endpoints reported in the reviewed studies is presented in Table 1, summarizing EC_50_/LC_50_ values before and after advanced oxidation or photocatalytic treatment [51,52,53,54,55,56].

## 2. Sources, Environmental Fate, and Soil Entry of Emerging Drug and Dye Pollutants

### 2.1. Sources and Release Pathways

**Figure 3:**
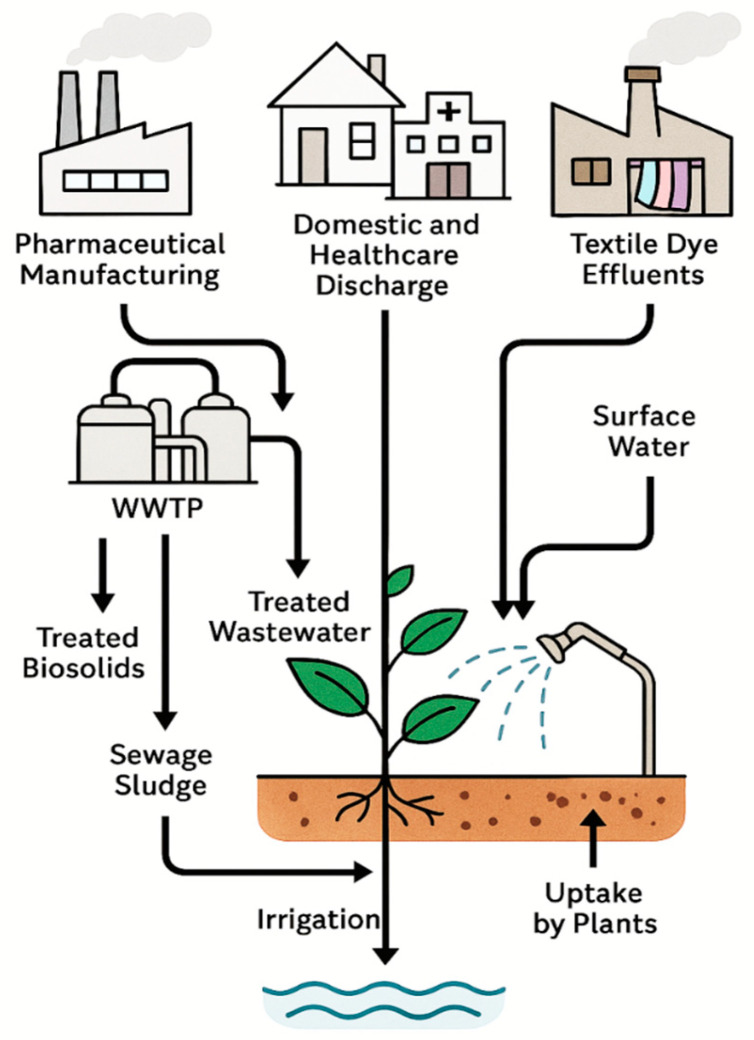
Schematic representation of the main sources and environmental pathways through which pharmaceutical and dye pollutants enter plant–soil systems via wastewater, biosolids, and irrigation processes.

The release of pharmaceuticals and synthetic dyes into the environment originates from multiple anthropogenic activities that together create a continuous input of micropollutants into aquatic and terrestrial ecosystems. These compounds reach soils and plants through diverse, interconnected pathways, depending on their origin, physicochemical properties, and the management practices applied to wastewater and solid residues. The release and environmental spreading of pharmaceuticals and synthetic dyes occur through multiple interconnected sources and pathways, linking industrial, domestic, and agricultural activities [21,31]. These pollutants enter aquatic environments via effluents from manufacturing facilities, household and healthcare discharges, and textile operations, before reaching soils and plants through irrigation and biosolid application. Figure 3 illustrates the major sources, environmental routes, and points of entry of emerging drug and dye pollutants into the plant–soil continuum, emphasizing both root uptake from contaminated soils and foliar absorption from aerial irrigation.

#### 2.1.1. Major Dye Classes and Their Environmental Behaviour

Synthetic dyes used in textile, food, cosmetic, and paper industries constitute one of the most environmentally persistent groups of organic contaminants due to their structural complexity and resistance to biodegradation. The most widely produced industrial dyes fall into a few dominant structural categories, including azo dyes, anthraquinone dyes, xanthene dyes, and triphenylmethane dyes, each exhibiting distinct physicochemical properties that determine their fate in soil–water–plant systems [57,58].

**Azo dyes,** which account for more than 60% of global dye production, contain one or more –N=N– azo linkages and typically exhibit high polarity and strong water solubility. These dyes sorb to clay–organic matrices predominantly through electrostatic interactions and hydrogen bonding, with sorption enhanced in soils rich in organic carbon and iron/aluminum oxides [59]. Under aerobic conditions they persist due to the stability of the azo bond; however, under anaerobic or reducing conditions commonly found in sediments, sludge, and the rhizosphere, azo dyes undergo reductive cleavage, generating aromatic amines that may display higher mutagenicity or phytotoxicity than the parent dyes [60,61]. These aromatic amines can further participate in soil-binding reactions or undergo partial oxidation, forming quinone- and nitro-substituted intermediates with demonstrated ecotoxicological relevance.

**Anthraquinone dyes** possess fused aromatic ring systems that confer high photostability and low biodegradability. Their sorption to soils is governed by hydrophobic interactions and π–π stacking with organic matter, while their planar geometry facilitates strong binding to mineral surfaces [62]. Under sunlight, anthraquinone dyes may undergo photosensitized degradation, producing radicals and partially oxidized intermediates such as anthrones and benzoic acid derivatives. These transformation products exhibit variable toxicity, with several studies reporting increased oxidative stress responses in plants exposed to anthraquinone photoproducts [63].

**Xanthene dyes,** including rhodamine B and fluorescein derivatives, show high molar absorptivity and strong fluorescence, properties responsible for their widespread use but also for their ability to act as photosensitizers. Upon irradiation, these dyes can generate reactive oxygen species (ROS), inducing oxidative damage in microorganisms, aquatic invertebrates, and plant tissues [64,65]. Their sorption behavior is strongly pH-dependent due to multiple ionizable groups, leading to increased mobility under neutral to alkaline conditions.

**Triphenylmethane dyes,** such as malachite green and crystal violet, carry delocalized cationic charges that promote strong adsorption to negatively charged clay minerals and humic substances [66]. However, their cationic nature also enhances their interaction with biological membranes, often resulting in high acute toxicity. Photocatalytic and oxidative degradation of triphenylmethane dyes may produce carbinol, demethylated, or benzophenone-type intermediates, several of which retain antimicrobial and phytotoxic properties [67].

Overall, the environmental behavior of dyes is governed by their ionic state, aromaticity, and functional-group chemistry, which together determine their sorption, light reactivity, mobility, and transformation pathways. Their persistence and the formation of toxic intermediates underscore the need to evaluate dye degradation not only through color removal or parent-compound decay, but also through comprehensive transformation-product identification and ecotoxicological assessment, particularly using soil–plant systems.

#### 2.1.2. Major Pharmaceutical Classes and Their Environmental Behaviour

Pharmaceuticals detected in treated wastewater, biosolids, and agricultural soils encompass a broad range of therapeutic classes, including non-steroidal anti-inflammatory drugs (NSAIDs), antibiotics, antidepressants, anticonvulsants, and β-blockers. Their environmental behaviour is governed by their molecular structure, acidity/basicity (pKa), hydrophobicity (log Kow), and charge distribution, which together determine sorption affinity, mobility, persistence, and bioavailability in soil–plant systems [68,69].

NSAIDs such as diclofenac, ibuprofen, ketoprofen, and naproxen are widely reported in municipal effluents and agricultural soils. Many NSAIDs exist as anionic species at neutral pH, resulting in weak sorption to negatively charged clay minerals and enhanced mobility in sandy or alkaline soils [70]. Diclofenac is particularly persistent due to its chlorinated aromatic structure and steric hindrance, exhibiting limited biodegradability and strong resistance to conventional treatment [71].

Antibiotics, including sulfonamides, macrolides, tetracyclines, and fluoroquinolones, show highly variable environmental behaviour. Sulfonamides, weak acids with relatively low sorption coefficients, often leach through soil profiles and contaminate groundwater. In contrast, tetracyclines and fluoroquinolones strongly bind to clay minerals and organic matter through cation exchange, surface complexation, and hydrogen bonding, leading to long-term persistence in topsoil [72]. These compounds can accumulate in the rhizosphere, alter microbial community structure, and reduce nitrogen-cycling functionality [73].

Antidepressants (e.g., fluoxetine, sertraline, venlafaxine) are typically cationic at environmental pH values due to protonated amine groups. As a result, they show strong sorption to organic matter and fine-textured soils, but also a high affinity for biological membranes, often resulting in bioaccumulation and chronic toxic effects on plants and soil fauna [74].

Anticonvulsants, particularly carbamazepine, are among the most recalcitrant pharmaceuticals in wastewater treatment due to their low biodegradability, neutral charge, and high structural stability. Carbamazepine poor removal in WWTPs leads to its continual release to irrigation waters and agricultural soils, where it persists for long periods and can translocate into edible plant tissues [75,76].

β-blockers such as metoprolol and propranolol are moderately hydrophilic yet exhibit high sorption potential due to their aromatic moieties and protonated amine groups. Their oxidation during treatment may generate hydroxylated or quinone-type transformation products, some of which display higher phytotoxicity or genotoxicity than the parent compound [77,78].

Overall, pharmaceutical fate is dictated by their ionization state, aromaticity, functional groups, and interaction with soil minerals and organic carbon. Their persistence, sorption–desorption dynamics, and potential to form biologically active transformation products highlight the need to assess not only parent-compound removal but also the ecotoxicological relevance of transformation pathways in soil–plant systems.

#### 2.1.3. Pharmaceutical Manufacturing

Industrial discharges from pharmaceutical manufacturing plants are among the most concentrated point sources of APIs in the environment. When process water and waste streams are not adequately treated, effluents can contain exceptionally high concentrations of antibiotics, analgesics, antipyretics, and other drug residues, far exceeding those from domestic sources [35,44].

Localized contamination “hotspots” have been documented near bulk-drug production centers, especially in developing countries, where wastewater treatment capacity and enforcement are limited. In recent years, global health and environmental agencies have emphasized that manufacturing effluents can significantly contribute to the spread of biologically active substances, antimicrobial resistance genes, and residual toxicity in receiving waters and soils [36,37,46,47]. Strengthened process control, on-site pre-treatment, and zero-liquid-discharge technologies are now being prioritized to minimize emissions at the source.

#### 2.1.4. Domestic and Healthcare Discharge

At the global scale, the majority of pharmaceutical residues entering wastewater systems originate from households and healthcare facilities. After consumption, unmetabolized fractions of drugs are excreted or discarded via sinks and toilets, entering municipal sewers where conventional treatment only partially removes them.

Consequently, APIs and their metabolites are continuously detected in the effluents, sludges, and biosolids produced by wastewater treatment plants (WWTPs) [1,30,34]. These discharges form the dominant diffuse pathway of pharmaceuticals into aquatic environments and, subsequently, agricultural soils through the reuse of treated wastewater for irrigation or through the application of biosolids as fertilizers. European policy frameworks have recently acknowledged the environmental load from the household and medical sectors, encouraging source-control measures such as eco-pharmacovigilance and green pharmacy initiatives [38,79].

#### 2.1.5. Textile Dye Effluents

The textile and dyeing industries represent one of the largest point sources of synthetic organic dyes and auxiliary chemicals. During coloration processes, up to 20% of dyes used may not bind to fibers and are discharged with the effluent [10]. These wastewater streams are complex mixtures of organic and inorganic constituents, often containing salts, surfactants, and heavy-metal mordants. When inadequately treated, such effluents are released into surface waters, altering physicochemical characteristics such as color, light penetration, and oxygen demand, and introducing compounds that persist and bioaccumulate in sediments and soils [8,19].

Many dye molecules, particularly azo and anthraquinone structures, are resistant to biodegradation, enabling their transfer into soils through irrigation using contaminated water or by deposition of particulate residues on farmland.

#### 2.1.6. Pathways to Soils and Plants

The entry of these pollutants into the soil–plant continuum occurs through both direct and indirect mechanisms. The most widespread route is agricultural irrigation with reclaimed or partially treated wastewater, which carries residual pharmaceuticals, dyes, and their transformation products. Upon infiltration, these contaminants can adsorb to soil particles, undergo chemical or microbial transformation, or remain bioavailable for uptake by plant roots. In parallel, the application of sewage sludge or biosolids as soil amendments introduces sorbed pharmaceuticals and dyes directly into the topsoil, from which they may leach, volatilize, or be absorbed by crops [13,21,26].

Beyond root exposure, aerial irrigation systems such as sprinklers or high-pressure sprays create a secondary pathway by depositing contaminants onto leaf surfaces. Experimental evidence demonstrates that foliar uptake can occur when droplets containing hydrophilic APIs or soluble dyes adhere to the cuticle, leading to direct penetration through stomata or surface microcracks. This exposure route is particularly relevant for leafy vegetables and forage crops irrigated with treated or untreated wastewater, where spray irrigation facilitates both surface contamination and systemic absorption [5,12,13,14,15].

#### 2.1.7. Integrated Environmental Relevance

Once introduced into plant–soil systems, these compounds interact with soil organic matter and biota, affecting microbial processes, nutrient cycling, and plant physiology. Their persistence in soils and the repeated use of reclaimed water amplify long-term accumulation, while volatilization or runoff can redistribute them within the landscape.

Understanding these release and exposure pathways is therefore essential for evaluating environmental risks and for designing effective wastewater treatment and reuse strategies that prevent contaminant transfer to crops and soils [19,20,23].

### 2.2. Transport to Soil Systems

The environmental behavior of pharmaceuticals and dyes in plant–soil systems is largely governed by their physicochemical properties, which determine mobility, persistence, and bioavailability. Parameters such as hydrophobicity (log Kow), acid dissociation constant (pKa), and water solubility influence processes including adsorption to soil particles, leaching potential, volatilization, and plant uptake through roots or foliage. Table 2 summarizes representative compounds commonly detected in wastewater–impacted environments and highlights how their physicochemical traits shape their fate and interactions within soil–plant systems.

#### 2.2.1. Irrigation with Reclaimed Water

Reuse of treated wastewater (TWW) transfers residual pharmaceuticals and dyes (and their transformation products) from effluents to agricultural fields, where compounds partition among soil solids, porewater, and the rhizosphere. Field and greenhouse studies show plant exposure via root uptake under TWW irrigation, with compound-specific behavior governed by sorption, ionization, and persistence [16,44,85]. Synthesis papers highlight that while concentrations often decline along the water → soil → plant continuum, detectable residues and metabolites still enter edible tissues in some systems [45,86].

Large-scale surveys and case studies further indicate compound-dependent accumulation patterns and variability across crop types and management regimes [1,16]. For dyes, irrigation with dye-bearing waters alters soil chemistry and can affect crop performance and soil quality, underscoring transport with irrigation water and retention near the soil–water interface [15].

#### 2.2.2. Sludge/Biosolid Amendment

Land application of sewage sludge or biosolids introduces a different transport regime dominated by sorbed phases. Pharmaceuticals and dyes associated with organic matter and fine minerals in biosolids are transferred to topsoils, where gradual desorption and transformation control porewater exposure and potential root uptake [13,40,46].

Reviews emphasize that physicochemical properties (e.g., pKa, logKow, charge) and soil traits (organic carbon, clay content, pH) regulate sorption–desorption hysteresis and thus bioavailability after amendment [4,47,87]. The biosolids route also co-introduces complex mixtures (e.g., salts, metals, auxiliaries), potentially modifying transport by competition for sorption sites or by altering microbial communities that mediate [13,48,88].

#### 2.2.3. Infiltration and Leaching

Following irrigation or rainfall, infiltration drives vertical transport from the surface into the vadose zone. Mobility depends on aqueous speciation and sorption affinity: weakly sorbing, ionizable, or highly soluble compounds exhibit greater breakthrough, whereas strongly sorbing cationic species (e.g., fluoroquinolones) are often retained in upper horizons [4,47,87]. Experimental studies document leaching of selected pharmaceuticals under recycled-water irrigation scenarios, evidencing potential transfer below the root zone and toward groundwater when sorption is limited [45,49,86,89].

Hydrological factors (irrigation intensity, soil texture/structure, preferential flow) further modulate fluxes, with leaching recognized as a key driver of solute redistribution in irrigated agroecosystems [50,90]. For textile dyes, persistence and high polarity in some classes support retention at interfaces, but do not preclude transport of more mobile dye species or dye–auxiliary mixtures with percolating water, particularly in coarse-textured soils or under high irrigation loads [15,51,91]. Collectively, these pathways show that TWW irrigation supplies a continuous dissolved input to soils, biosolids deliver sorbed loads that can desorb over time, and infiltration/leaching redistributes contaminants through soil profiles. Transport and exposure are controlled by compound properties, soil characteristics, and water/irrigation regimes, determining the fraction that remains near roots, enters plants, or migrates below the root zone [4,47,52,87,92].

### 2.3. Chemical Persistence and Mobility

The persistence and mobility of pharmaceutical and dye pollutants in soils are controlled by a complex interplay of physicochemical and biological processes, including sorption–desorption dynamics, leaching through soil profiles, photolytic transformation, and microbial degradation. These processes determine whether contaminants remain bioavailable for plant uptake, accumulate in the rhizosphere, or migrate toward groundwater systems.

#### 2.3.1. Sorption and Desorption

Sorption is the primary mechanism limiting pollutant mobility in soils, governed by interactions between chemical functional groups and soil constituents such as organic carbon, clay minerals, and metal oxides. Compounds with higher hydrophobicity (log Kₒw > 3) and neutral charge, such as diclofenac and ibuprofen, tend to bind strongly to soil organic matter, whereas ionizable pharmaceuticals (e.g., sulfonamides, fluoroquinolones) exhibit pH-dependent sorption behavior due to ion exchange or complexation reactions [47,53,87,93].

Dyes, particularly cationic types such as methylene blue, adsorb effectively onto negatively charged clay surfaces, while anionic azo dyes display weaker interactions and greater potential for leaching [8,90]. Sorption–desorption hysteresis is common, indicating partial irreversibility and strong retention in the solid phase, but environmental fluctuations (e.g., moisture, temperature, competing ions) can promote remobilization [4].

#### 2.3.2. Leaching and Transport

Leaching represents a major route for downward pollutant movement into subsurface soils and groundwater. The extent of leaching depends on solubility, charge, and soil texture. Weakly sorbing and highly soluble compounds such as carbamazepine or atenolol demonstrate higher leaching potential under irrigation or rainfall [49,54,89,94]. Conversely, strongly adsorbed species like ciprofloxacin or cationic dyes remain concentrated in surface layers [50,90]. Soil hydraulic properties, particularly preferential flow through macropores accelerate transport of pharmaceuticals and dyes beyond the root zone, especially in coarse-textured or heavily irrigated soils [47,50,87,90].

#### 2.3.3. Photolysis and Abiotic Degradation

Exposure to sunlight at the soil surface or during irrigation contributes to the degradation of light-sensitive compounds. Photolysis plays an important role for dyes such as Rhodamine B and Reactive Black 5, as well as for pharmaceuticals with aromatic or halogenated structures capable of absorbing UV–visible radiation [55,95].

However, the efficiency of photodegradation depends on soil surface properties, moisture content, and shading by vegetation or organic matter. Photoproducts may display altered polarity or toxicity, and some persist longer than parent compounds, extending ecological risk [55,56,95,96].

#### 2.3.4. Microbial Degradation

Microbial transformation is a dominant natural attenuation pathway, though degradation rates vary widely across compound classes and soil types. Aerobic degradation often proceeds through oxidation [48,88]. Pharmaceuticals such as sulfamethoxazole and fluoroquinolones are particularly resistant to microbial attack due to their complex ring structures and antimicrobial properties that suppress degraders [4]. For textile dyes, microbial decolorization can occur through reductive cleavage of azo bonds under anaerobic conditions, yet complete mineralization is rare and dependent on microbial community composition and enzyme availability [23,51,66,91]. Bioavailability is further influenced by sorption: compounds strongly bound to soil particles are less accessible for microbial degradation, enhancing persistence.

**Figure 4:**
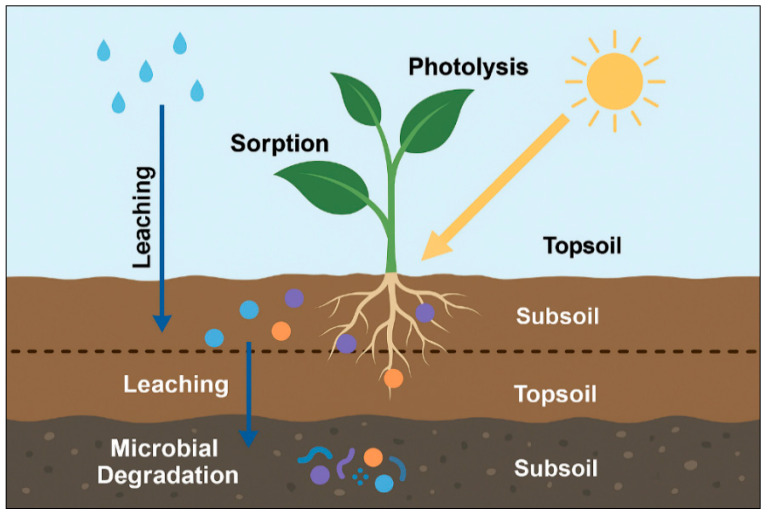
Conceptual representation of the main processes influencing the persistence and mobility of pharmaceutical and dye pollutants in soils, including sorption, leaching, photolysis, and microbial degradation pathways.

The environmental persistence and movement of pharmaceuticals and dyes in soils result from a combination of physicochemical interactions and biological transformations that determine their long–term fate and ecological impact. These processes include sorption to soil particles, downward leaching through soil profiles, surface photolysis under sunlight exposure, and microbial degradation within the rhizosphere. Figure 4 conceptually illustrates these interconnected mechanisms, showing how compound-specific properties and soil characteristics control pollutant retention, transformation, and mobility in agricultural soils.

Overall, chemical persistence and mobility in soils are tightly coupled to compound structure and environmental conditions. Weakly sorbed, hydrophilic, or ionized substances tend to be more mobile and persistent, while strongly adsorbed compounds accumulate in surface soils with limited biodegradation. Understanding these coupled mechanisms is essential for predicting long-term pollutant dynamics and assessing risks to soil health and plant systems.

### 2.4. Influence of Soil Properties—pH, Texture, Organic Matter, and Cation Exchange Capacity Affecting Pollutant Bioavailability

The fate, persistence, and bioavailability of pharmaceutical and dye pollutants in soils are strongly influenced by intrinsic soil properties such as pH, texture, organic matter content, and cation exchange capacity (CEC). These parameters determine the balance between sorbed and dissolved fractions, thereby modulating pollutant accessibility to soil microorganisms and plant roots [19,87].

The influence of soil physicochemical properties on the fate and bioavailability of pharmaceutical and dye pollutants is well-documented across diverse soil types and environmental settings. Variations in pH, texture, organic matter content, and cation exchange capacity (CEC) determine the degree of pollutant sorption, mobility, and persistence, shaping their ecological impacts and potential for plant uptake. Table 3 compiles representative experimental data from recent literature, illustrating how these parameters quantitatively influence pollutant partitioning, retention, and transport behavior in soils.

#### 2.4.1. Soil pH

Soil pH governs the ionization state of both contaminants and soil surfaces, directly affecting adsorption, desorption, and solubility. Weakly acidic pharmaceuticals such as diclofenac or ibuprofen are predominantly anionic at neutral to alkaline pH, resulting in reduced sorption to negatively charged soil particles and greater mobility [4,47,87].

Conversely, cationic species such as fluoroquinolones and basic dyes (e.g., methylene blue) exhibit stronger binding in neutral to alkaline conditions due to electrostatic attraction [8,61,101]. Under acidic conditions, protonation of soil surfaces diminishes negative charge density, promoting sorption of anionic compounds but limiting adsorption of cationic ones. The overall effect is that pH fluctuations within typical agricultural ranges (5.5–8.5) can markedly alter contaminant partitioning and bioavailability [47,87].

#### 2.4.2. Soil Texture

The particle size distribution of soils affects both retention and transport of micropollutants. Fine-textured soils (rich in clays and silts) possess higher surface area and reactive sites for sorption, thus retaining hydrophobic and ionizable compounds more effectively than coarse-textured sandy soils [50,90].

However, preferential flow pathways in structured clays may facilitate rapid vertical transport of weakly sorbing pharmaceuticals under intense irrigation or rainfall [49,89]. In contrast, sandy soils have lower sorption capacity and higher hydraulic conductivity, increasing the likelihood of leaching of soluble and weakly sorbing contaminants such as carbamazepine, sulfamethoxazole, and some hydrophilic dyes [4,54,94].

#### 2.4.3. Soil Organic Matter (SOM)

SOM plays a dual role as both a sorbent and a substrate for microbial degradation. The hydrophobic domains of humic and fulvic substances provide binding sites for nonpolar compounds, while polar functional groups interact with ionizable pollutants through hydrogen bonding and complexation [47,87]. High SOM content typically reduces pollutant mobility by increasing sorption and sequestration, thereby lowering immediate bioavailability but potentially prolonging persistence through reduced biodegradation rates [48,88].

In contrast, soils with low organic carbon exhibit weaker sorptive capacity, facilitating faster pollutant transport and root exposure [61,101]. SOM composition, particularly the aromatic-to-aliphatic carbon ratio, further influences sorption mechanisms and pollutant partition coefficients.

#### 2.4.4. Cation Exchange Capacity (CEC)

CEC reflects the soil’s ability to retain and exchange cations, influencing electrostatic interactions with charged pollutants. High-CEC soils, usually rich in clay minerals or organic colloids, favor retention of cationic species such as fluoroquinolones, β-blockers, and cationic dyes via ion exchange and surface complexation [4,8].

Conversely, in low-CEC sandy soils, these interactions are weaker, enhancing leaching potential. Moreover, multivalent cations (Ca^2+^, Fe^3+^, Al^3+^) can form bridges between pollutants and soil colloids, strengthening sorption and reducing bioavailability [47,87].

#### 2.4.5. Integrated Implications for Bioavailability

The combined influence of pH, texture, SOM, and CEC determines whether contaminants remain in soil solution, are immobilized on solids, or are accessible for plant uptake and microbial degradation. Generally, soils with neutral to alkaline pH, high clay or organic matter content, and high CEC immobilize pharmaceuticals and dyes more effectively, reducing immediate bioavailability but promoting long-term accumulation. In contrast, coarse, acidic, low-CEC soils enhance mobility and bioavailability, increasing the potential for plant uptake and leaching into deeper layers. These interactions highlight the importance of site-specific soil characterization in evaluating ecological risks and in managing the reuse of treated wastewater or biosolids in agriculture [26,96].

**Figure 5:**
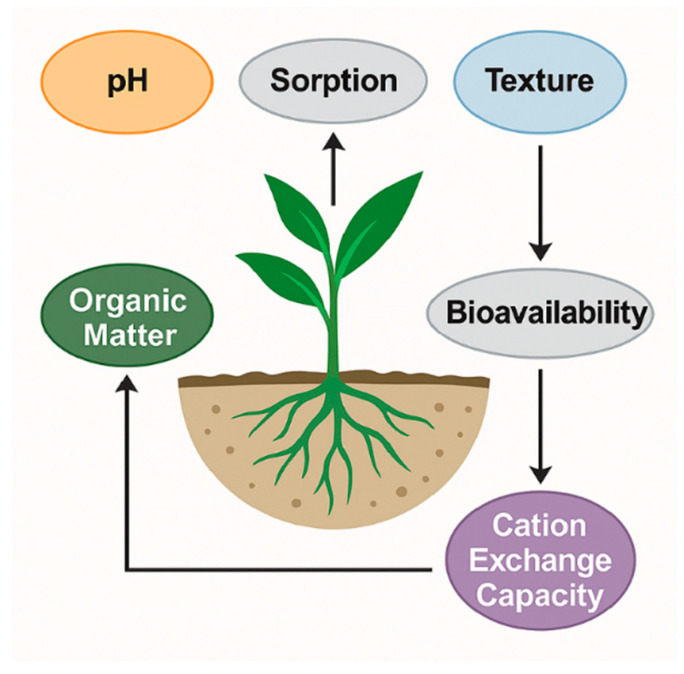
Conceptual framework showing how key soil properties (pH, texture, organic matter, and cation exchange capacity) affect the sorption, mobility, and bioavailability of pharmaceutical and dye pollutants in soil–plant systems.

The interaction between soil properties and pollutant behavior governs the extent to which pharmaceuticals and dyes remain mobile, persist in the soil matrix, or become bioavailable for plant uptake. Parameters such as soil pH, organic matter content, texture, and cation exchange capacity (CEC) act synergistically to control sorption–desorption dynamics, transformation rates, and root accessibility. Figure 5 conceptually illustrates how these soil characteristics influence pollutant retention and mobility, highlighting the contrasting behavior of hydrophobic versus hydrophilic and ionic compounds across different soil conditions.

## 3. Plant Uptake and Phytotoxicity of Drugs and Dyes in Contaminated Soils

### 3.1. Uptake Mechanisms—Root Absorption, Xylem Transport, Translocation, and Sequestration

The uptake and internal distribution of pharmaceuticals and synthetic dyes by plants are governed by complex physicochemical interactions at the soil–root interface and within plant tissues. These mechanisms include root absorption, xylem loading and upward transport, phloem redistribution, and cellular sequestration, all of which depend on compound properties (e.g., polarity, charge, molecular weight) and plant physiology [62,63,102,103].

#### 3.1.1. Root Absorption

Root uptake represents the primary pathway for contaminant entry into plants from soil or soil pore water. Pharmaceuticals and dyes in the dissolved phase can penetrate root epidermal cells via passive diffusion, facilitated transport, or ion trapping, depending on their speciation and lipophilicity [54,64,94,104]. Weakly acidic pharmaceuticals such as diclofenac and ibuprofen tend to be absorbed more readily at low pH, where the neutral form predominates, enhancing membrane permeability [65,105].

Conversely, cationic compounds such as fluoroquinolones or cationic dyes like methylene blue interact electrostatically with negatively charged cell walls and root exudates, reducing transmembrane diffusion but promoting surface binding and rhizosphere retention [66,106]. Hydrophobicity, expressed by log Kow, also plays a critical role: compounds with log Kow ≈ 2–4 show optimal uptake, whereas highly hydrophilic or hydrophobic compounds display reduced permeability [62,102].

#### 3.1.2. Xylem Transport and Translocation

Once absorbed, compounds may be loaded into the xylem stream and transported upward with the transpiration flow. This process primarily depends on compound polarity, molecular size, and ionization state [62,102]. Neutral or weakly polar molecules such as carbamazepine, caffeine, and sulfamethoxazole have been frequently detected in shoots and leaves, indicating efficient xylem mobility [65]. In contrast, strongly sorbing or ionized compounds (e.g., ciprofloxacin, methylene blue) tend to remain confined to root tissues.

Translocation factors (TFs), defined as the shoot/root concentration ratio, are often below 0.1 for highly sorptive compounds but may exceed 1 for hydrophilic or uncharged species [63]. The transpiration stream concentration factor (TSCF) further reflects the efficiency of transport through the xylem and correlates with molecular hydrophobicity and acidity.

#### 3.1.3. Phloem Redistribution and Sequestration

Some compounds can undergo limited redistribution through the phloem, particularly those with amphiphilic properties or molecular structures resembling endogenous metabolites [67,107]. Following uptake, pharmaceuticals and dyes may be sequestered into vacuoles, bound to cell wall components, or conjugated enzymatically into less bioavailable forms [62,102].

Vacuolar sequestration reduces cytoplasmic toxicity and prevents interference with primary metabolism. In the case of dyes, conjugation with organic acids or integration into lignin-like structures contributes to long-term retention within tissues. These mechanisms are analogous to plant detoxification pathways for xenobiotics, involving glutathione S-transferase or UDP–glucosyltransferase–mediated conjugation [64,104].

**Figure 6:**
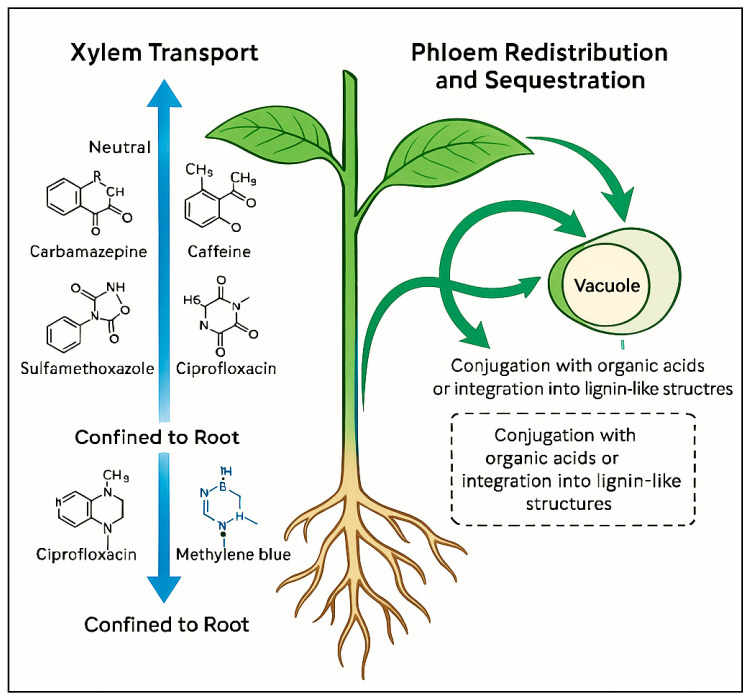
Schematic illustration of xylem transport and phloem redistribution of pharmaceuticals and dyes in plants, highlighting the upward movement of neutral compounds, root confinement of strongly sorbing species, and vacuolar sequestration or conjugation of amphiphilic molecules.

Figure 6 summarizes the internal movement of pharmaceuticals and dyes within plant tissues. The illustration highlights the contrasting behaviors of neutral, weakly polar compounds that readily move through the xylem toward aerial organs, compared with strongly sorbing or ionized species that remain confined to root tissues. It also depicts the limited phloem redistribution observed for amphiphilic molecules and the subsequent sequestration processes, such as vacuolar storage and conjugation that reduce cytoplasmic toxicity and contribute to long-term retention within plant cells.

#### 3.1.4. Integrated Dynamics

The relative importance of these mechanisms varies with species and growth stage. Fast-growing crops with high transpiration rates (e.g., lettuce, spinach, maize) show greater systemic transport and accumulation than woody species or deep-rooted plants [16,67,107]. Dyes, due to their larger molecular size and lower solubility, generally exhibit limited systemic mobility but strong rhizosphere adsorption and localized accumulation in roots. The balance between absorption, translocation, and sequestration ultimately determines not only plant exposure but also pollutant persistence within agricultural systems.

**Figure 7:**
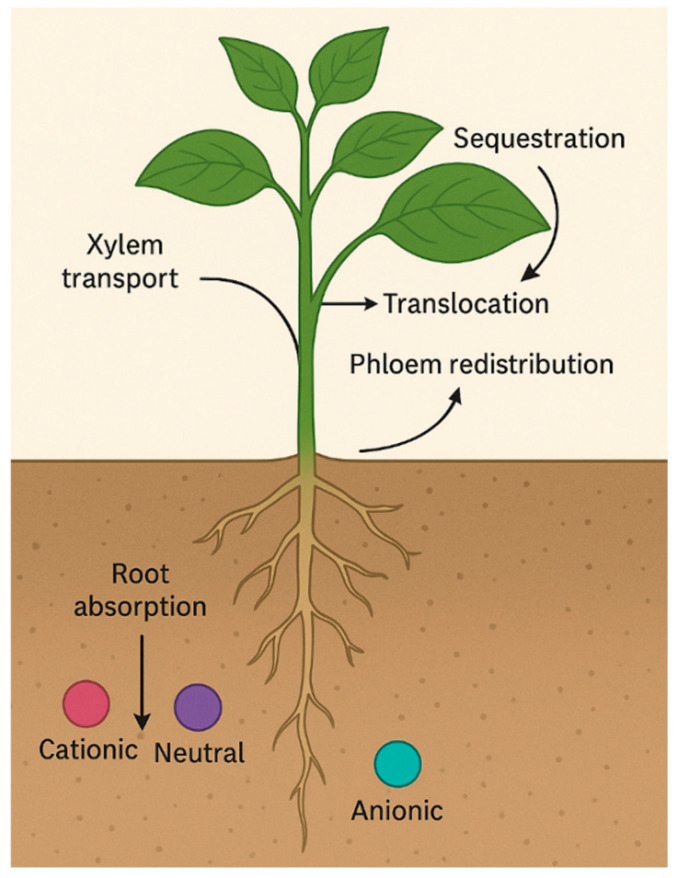
Conceptual illustration of the uptake and translocation mechanisms of pharmaceuticals and dyes in plants, showing root absorption, xylem transport, phloem redistribution, and cellular sequestration processes.

The movement of pharmaceuticals and dyes within plants involves multiple interacting physiological and physicochemical processes that determine their accumulation patterns and potential toxicity [19,31]. These include root absorption from soil pore water, translocation through xylem and phloem tissues, and subsequent sequestration or transformation within cellular compartments. Figure 7 schematically illustrates these uptake mechanisms, highlighting the main pathways from root surface adsorption and membrane transport to systemic distribution and vacuolar storage, emphasizing differences between hydrophilic and hydrophobic properties.

Understanding these mechanisms is essential for assessing the potential for contaminant transfer into food chains and for evaluating phytoremediation strategies that exploit plant uptake while minimizing ecotoxicological risks.

### 3.2. Phytotoxicity Endpoints—Germination, Biomass, Pigment Content, Oxidative Stress, Genotoxicity

The exposure of plants to pharmaceuticals and synthetic dyes through contaminated soils or irrigation water triggers a variety of phytotoxic responses that affect germination, growth, physiology, and genetic stability. The severity of these effects depends on contaminant type, concentration, plant species, and exposure duration. Common endpoints used to assess phytotoxicity include seed germination, biomass accumulation, photosynthetic pigment content, oxidative stress biomarkers, and genotoxic indicators, each providing insight into plant tolerance and potential ecological risks [45,68,86,108].

**Figure 8:**
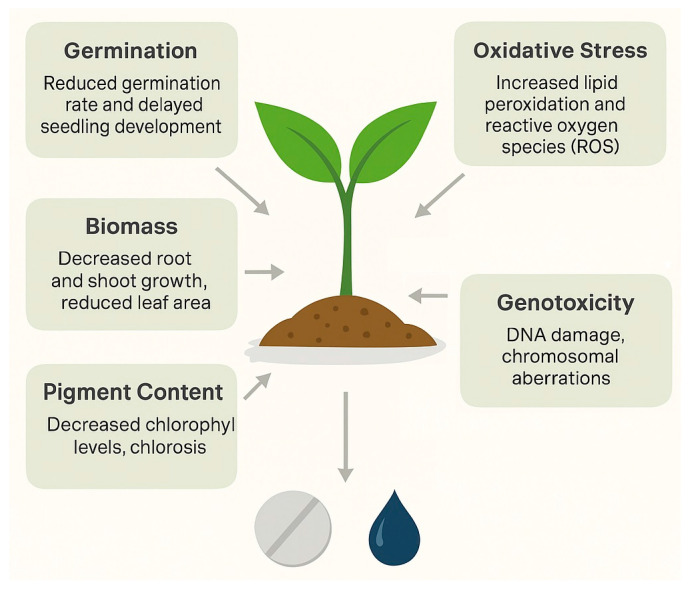
Overview of the main phytotoxicity endpoints associated with pharmaceutical and dye pollutants in plants, illustrating their effects on germination, biomass, pigment content, oxidative stress responses, and genotoxic alterations.

Phytotoxicity induced by pharmaceuticals and synthetic dyes manifests through multiple, interconnected physiological and biochemical pathways in plants. These include early developmental inhibition during germination, reductions in biomass and photosynthetic pigments, oxidative stress marked by reactive oxygen species (ROS) accumulation, and genotoxic effects at the cellular level. Figure 8 provides a conceptual overview of these key toxicity endpoints and their underlying mechanisms, highlighting how pollutant exposure disrupts growth, metabolism, and genomic integrity across plant organs.

#### 3.2.1. Seed Germination and Early Growth

Germination is one of the most sensitive stages in plant development and serves as an early bioindicator of soil contamination. Pharmaceuticals such as diclofenac, ibuprofen, and carbamazepine have been shown to delay germination, reduce radicle elongation, and impair seedling vigor, particularly in lettuce (*Lactuca sativa*), wheat (*Triticum aestivum*), and maize (*Zea mays*) [69,70,109,110].

Synthetic dyes such as methylene blue, crystal violet, and reactive black 5 can interfere with water uptake and cell division in embryonic tissues, reducing germination rates by up to 60% under high exposure levels [71,111]. The inhibition is often linked to osmotic stress, changes in enzymatic activity, or physical blockage of root pores due to dye adsorption.

#### 3.2.2. Biomass and Morphological Responses

Reductions in root and shoot biomass are frequently reported following exposure to pharmaceutical residues or dye pollutants. For example, exposure to fluoroquinolones and β-blockers significantly decreased biomass in spinach and mustard, correlating with inhibited nutrient uptake and impaired root growth [67,107].

Dyes such as malachite green and methylene blue reduce chlorophyll synthesis and alter leaf morphology, resulting in chlorosis, necrosis, and reduced leaf area [8]. Morphological deformities, such as twisted roots or shortened hypocotyls, have been attributed to hormonal disruption and interference with auxin transport.

#### 3.2.3. Pigment Content and Photosynthetic Efficiency

Alterations in chlorophyll and carotenoid content are widely recognized as indicators of stress caused by xenobiotic exposure. Non-steroidal anti-inflammatory drugs (NSAIDs) like diclofenac and ibuprofen disrupt chlorophyll biosynthesis and electron transport chains, leading to a decline in photosystem II activity.

Dyes that absorb visible light (e.g., azo and anthraquinone dyes) can physically reduce light availability and induce photooxidative stress in leaves [72,112]. Reduced chlorophyll a/b ratios and fluorescence parameters (Fv/Fm) indicate impaired photochemical efficiency and oxidative damage to thylakoid membranes.

#### 3.2.4. Oxidative Stress and Antioxidant Responses

Pharmaceuticals and dyes can induce reactive oxygen species (ROS) accumulation, resulting in lipid peroxidation, protein oxidation, and membrane injury. Plants respond by activating antioxidant defense systems, including enzymes such as superoxide dismutase (SOD), catalase (CAT), and peroxidases (POD) [73,113].

Elevated malondialdehyde (MDA) levels in exposed plants indicate lipid peroxidation and oxidative imbalance. Antibiotics such as sulfamethoxazole and ciprofloxacin have been reported to increase ROS production and disrupt redox homeostasis in *Arabidopsis thaliana* and *Brassica napus* [74,114]. Similarly, dye exposure triggers ROS generation via photoexcitation, amplifying oxidative stress and metabolic inhibition.

#### 3.2.5. Genotoxic and Cytological Effects

Genotoxicity represents a more persistent and often irreversible endpoint of pollutant exposure. Pharmaceuticals and dyes can cause chromosomal aberrations, DNA strand breaks, and micronucleus formation in root meristematic cells [75,76,115,116]. Studies using *Allium cepa* assays have demonstrated that fluoroquinolones and cationic dyes disrupt spindle formation and DNA integrity, reducing the mitotic index and inducing nuclear abnormalities.

Reactive intermediates generated during dye degradation (e.g., aromatic amines) can further enhance genotoxic potential. Such genetic damage not only compromises plant development but also poses long-term ecological consequences if heritable mutations occur. These phytotoxicity indicators provide a multidimensional understanding of plant stress responses to pharmaceutical and dye pollutants. While sublethal concentrations may not visibly impair growth, biochemical and molecular disruptions can still occur, influencing plant productivity and ecosystem stability. The combination of physiological (growth, pigments) and biochemical (ROS, enzymes) endpoints, alongside cytogenetic assays, offers a comprehensive approach to evaluating plant tolerance and environmental safety in contaminated soils.

### 3.3. Interactions with Soil Microbiota—Indirect Effects Through Microbiome Disruption

Soil and rhizosphere microbiomes regulate nutrient cycling, disease suppression, and plant growth; disturbances from pharmaceutical and dye pollutants can therefore trigger indirect phytotoxicity even when plant exposure is limited [77,117]. Reuse of treated wastewater and land application of biosolids introduce complex contaminant mixtures that restructure bacterial communities, enrich antibiotic-resistance determinants, and shift metabolic functions critical for nitrogen and carbon turnover [21,23,78,118].

Long-term irrigation with secondary treated wastewater, for example, has been associated with changes in soil physicochemistry, increased tolerance to metals/antibiotics, and altered diversity–function relationships, indicating sustained selection pressures in the microbiome [21,23]. Historic and contemporary evidence also shows that wastewater irrigation elevates the abundance of antibiotic resistance genes (ARGs) in soils and on crops, with the magnitude related to irrigation intensity and ARG loads in the water matrix [78,79,118,119]. At the process level, pharmaceuticals can inhibit key nitrogen-cycling guilds (ammonia- and nitrite-oxidizers; denitrifiers), depress nitrification/denitrification enzyme activities, and perturb N_2_O pathways, effects that cascade into nutrient stress for plants [19,20]. These responses are compound- and dose-dependent, reflecting a combination of direct antibiotic pressure and indirect competition/selection within the community. In parallel, textile dyes, especially azo and cationic dyes, interact with microbial cell envelopes and redox enzymes, suppressing basal respiration and altering phospholipid fatty-acid profiles; persistent chromophores can reduce light/oxygen microgradients and further impair heterotrophic activity [51,91]. While specialized consortia can reductively cleave azo bonds and decolorize effluents, incomplete mineralization and intermediates (e.g., aromatic amines) may continue to disrupt microbial networks and soil enzyme systems [80,81,120,121].

Microbiome disruption feeds back to plant performance through several pathways: (i) reduced nutrient provisioning (e.g., N availability) and impaired cycling; (ii) weakened biocontrol against pathogens as beneficial taxa decline; (iii) altered root exudation driven by plant stress, which can further reshape rhizosphere assembly; and (iv) changes in microbial signaling and hormone analogs affecting root architecture [77,117]. Collectively, these findings indicate that even when direct plant uptake is modest, microbiome-mediated effects can diminish plant vigor and resilience in soils receiving pharmaceutical- or dye-bearing inputs, underscoring the need to combine chemical monitoring with functional microbial endpoints in risk assessment [19,20,21,23].

To ensure consistency and reproducibility in assessing microbiome-mediated effects of pharmaceutical and dye pollutants on plant–soil systems, it is essential to harmonize experimental and reporting practices. Variations in soil properties, molecular techniques, and analytical workflows often hinder cross-study comparisons and mechanistic understanding. Box 1 summarizes key microbiological, biochemical, and molecular endpoints relevant for evaluating soil microbiome disruption, alongside a checklist of minimal reporting requirements and recommended visualization formats. This framework supports standardized assessment of microbial responses and strengthens the integration of plant–microbe interactions in ecotoxicological research.

Box 1 **Recommended microbiome endpoints and reporting checklist.**

Core endpoints (select at least one from each block)

Community structure

16S rRNA (bacteria/archaea), ITS (fungi); alpha (Shannon, Simpson) and beta diversity (Bray–Curtis, UniFrac); differential taxa.

Functional potential (genes)

N-cycle: amoA, nirK/nirS, nosZ, nifH; C-cycle: mcrA, pmoA, CAZy families; ARGs/MGEs by qPCR or metagenomics.

Activity & physiology

Soil enzyme assays (urease, β-glucosidase, dehydrogenase, phosphatase); microbial respiration (basal/substrate-induced); PLFA/FAME for biomass.

Rhizosphere interactions

Root exudate profiling (TOC, metabolites); root colonization (microscopy/qPCR).

Co-endpoints for plant health

N availability (NH_4_^+^, NO_3_^−^), mineral N transformations, tissue N/P, biomass, photosynthetic efficiency (Fv/Fm).

Minimal reporting checklist (to enable cross-study comparison)

1. Site & matrix

Soil type, texture, pH, EC, CEC, TOC, moisture, temperature, irrigation/sludge history.

2. Exposure

Source and chemistry of reclaimed water/sludge, contaminant concentrations, dose, frequency, duration.

3. Design

Plot/pot layout, replication (≥3), randomization, controls (clean water/matrix), blocking.

4. Sampling

Depth, rhizosphere vs. bulk soil, time points (baseline + ≥2 post-exposure).

5. Molecular methods

Extraction kit, primers, target region, sequencing platform, negative/positive controls.

6. Bioinformatics

Pipeline, databases, normalization, contaminant filtering, versioning.

7. qPCR/metagenomics

Standards, efficiencies, normalization (per g soil or ng DNA), internal controls.

8. Enzyme/respiration assays

Units, substrate, incubation time, blanks, calibration, temperature.

9. Statistics

Model specification, multiple testing correction, effect sizes, PERMANOVA.

10. Data availability

Raw reads, metadata, code, and workflow DOI.

Suggested visualization set

Stacked barplots (phylum/genus); PCoA/NMDS ordinations; volcano or MA plots; heatmaps for ARGs/functional genes; co-occurrence networks (SparCC/SPIEC-EASI) with caution; forest plots for enzyme activity effect sizes.

### 3.4. Dose–Response Relationships—Influence of Pollutant Concentration and Exposure Duration

Quantitative data on concentration–effect patterns, hormetic responses, exposure duration, and mixture toxicity provide valuable insights into the ecological relevance of emerging pollutants in plant–soil systems. The evidence compiled in Table 4 summarizes experimentally derived dose–response data for representative pharmaceuticals and dyes, illustrating how pollutant concentration, exposure time, and physicochemical interactions determine phytotoxic outcomes.

This synthesis highlights the variability in ECx thresholds across plant species and compound classes, the occurrence of hormesis at low doses, and the amplification of toxic effects under prolonged or mixed exposures, emphasizing the importance of integrated assessment frameworks in evaluating pollutant risks to terrestrial vegetation.

#### 3.4.1. Concentration–Effect Patterns and Thresholds

For pharmaceuticals and dyes in soil–plant systems, responses typically follow sigmoidal (Hill/Weibull) curves, allowing for derivation of ECx (e.g., EC10, EC50), NOEC and LOEC values for germination, biomass, pigment content and oxidative-stress endpoints.

Across crop and wild species, oxytetracycline (OTC) shows wide interspecific variability: a recent comparative assessment reported EC10 ranges of 0.39–26.64 mg L^−1^ and EC50 ranges of 18.0–846.78 mg L^−1^ in crops, and EC_10_ 0.18–64.34 mg L^−1^ with EC50 46.02–2611.49 mg L^−1^ in wild species [82,122]. Weakly sorbing, neutral or anionic compounds (e.g., carbamazepine, some sulfonamides) often display lower ECx in sandy/low-organic soils because higher pore-water availability increases root exposure, whereas strongly sorbing cationic species (e.g., fluoroquinolones; cationic dyes) tend to show higher ECx for shoot endpoints but strong root-localized effects [47,87].

#### 3.4.2. Hormesis and Low-Dose Stimulation

Several studies describe biphasic responses in which sub-inhibitory concentrations stimulate seedling vigour or biomass, while higher doses are inhibitory, as a hormetic pattern. For sulfamethoxazole (SMX), low soil doses enhanced growth in sorghum, but ≥25 mg kg^−1^ significantly reduced germination and biomass; microplastics modulated SMX bioavailability and attenuated toxicity [83,123]. A broader toxicology synthesis likewise notes low-dose stimulation with high-dose inhibition for SMX and related antibiotics in plants [84,124].

#### 3.4.3. Exposure Duration and Time-Dependent Toxicity

Extending exposure from acute to sub-chronic/chronic windows generally lowers apparent thresholds (time-dependent toxicity) and increases the probability of carry–over stress (e.g., ROS accumulation, pigment loss). In vegetable crops exposed to SMX, multi-week exposure altered rhizosphere composition and increased resistance-gene abundances, coincident with growth and physiological impairment compared to short exposure [85,125].

For rice, the SMX metabolite N_4_–acetyl–SMX formed over time and contributed to toxicity, underscoring that transformation products can shift the dose–response during longer exposures [86,126]. For dyes, acute tests often show strong concentration dependence (e.g., methylene blue in primary producers), while longer soil exposures highlight indirect effects (light attenuation, redox changes) that compound dose responses [87,90,127,130].

#### 3.4.4. Mixtures and Interaction Effects

Environmentally relevant exposures are mixtures. Binary/complex mixtures of NSAIDs or antibiotics can produce additive to synergistic phytotoxicity depending on ratios, with diclofenac frequently driving mixture toxicity at shared targets [88,89,128,129]. Consequently, single–compound EC_x_ may underestimate inhibition in reclaimed-water or biosolids scenarios where multiple residues co-occur.

#### 3.4.5. Modeling Implications

For regulatory comparison, ECx from standard endpoints (germination, biomass, F_v_/F_m_, MDA, SOD/CAT/POD) should be fitted with four-parameter log-logistic or Weibull models; when hormesis is evident, Brain–Cousens models are more appropriate. Where transformation products arise during the test, time-weighted average (TWA) concentrations or benchmark dose (BMD) approaches better represent exposure than nominal starting concentrations [62,85,86,102,125,126].

### 3.5. Soil Modulation of Toxicity—Adsorption/Desorption Dynamics and Pollutant Bioavailability

The toxicity of pharmaceutical and dye pollutants in plant–soil systems is not governed solely by the intrinsic chemical properties of the compounds but is strongly modulated by the adsorption–desorption equilibria within soil matrices. Adsorption reduces the freely available pollutant fraction in soil solution, whereas desorption or remobilization processes restore availability, driving the time-dependent exposure profile that plants and microorganisms experience [47,87]. The balance between these processes determines the bioaccessible fraction, which more accurately reflects ecological risk than total concentration.

#### 3.5.1. Adsorption Mechanisms and Influencing Factors

Soil organic matter (SOM), clay minerals, and iron/aluminum oxides provide the primary reactive surfaces for adsorption. Hydrophobic interactions dominate the retention of non-polar compounds, while electrostatic attraction or cation exchange governs the binding of charged pharmaceuticals and dyes. For example, the fluoroquinolone antibiotic ciprofloxacin exhibits strong sorption to soils rich in organic carbon and clay minerals due to cation bridging and surface complexation, substantially limiting its mobility and bioavailability [91,131]. Similarly, cationic dyes such as methylene blue or malachite green are efficiently adsorbed by negatively charged clay surfaces and humic colloids, leading to strong immobilization but potential persistence under changing redox conditions [92,132]. Soil pH and ionic strength critically influence adsorption.

Increasing pH enhances deprotonation of soil functional groups, thereby increasing negative charge and favoring cationic pollutant adsorption, while reducing the sorption of anionic species such as diclofenac [8,47,87]. Ionic strength and competing cations (Ca^2+^, Na^+^) may screen electrostatic interactions, decreasing adsorption and enhancing mobility, particularly in saline or reclaimed-water-irrigated soils [4].

#### 3.5.2. Desorption and Bioavailability

Desorption processes, often non-linear and hysteretic, control pollutant persistence in the soil solution phase. Strongly bound residues are desorbed slowly, reducing short-term toxicity but potentially prolonging long-term exposure [61,101].

Temperature and moisture fluctuations can further modulate desorption rates, while repeated wetting–drying cycles or organic amendments may remobilize previously sorbed residues. In particular, the addition of dissolved organic matter (DOM) can enhance the desorption and mobility of hydrophobic compounds, increasing their bioavailability to plants and soil microbes [93,133].

#### 3.5.3. Implications for Plant Uptake and Soil Toxicity

Bioavailability-controlled toxicity explains why similar nominal concentrations can yield divergent phytotoxic responses across soils of differing texture and organic carbon content. In fine-textured or organic-rich soils, adsorption reduces the freely available fraction of sulfamethoxazole or carbamazepine, lowering acute phytotoxicity but enabling slow desorption and chronic exposure [33,39].

Conversely, in sandy or low-CEC soils, the reduced adsorption capacity increases the soluble fraction, amplifying root exposure and translocation. Photocatalytic degradation products may also display altered sorption behavior, more polar intermediates often show reduced soil retention, thereby temporarily increasing bioavailability post-treatment [93,133].

#### 3.5.4. Environmental Dynamics and Risk Perspective

Overall, adsorption–desorption dynamics create a dynamic equilibrium that mediates short-term detoxification and long-term persistence. Risk assessments and plant uptake models should therefore incorporate site-specific soil parameters, particularly pH, SOM, texture, and CEC, to predict realistic exposure scenarios.

Advanced characterization approaches, such as sequential extraction and passive sampling, are increasingly recommended to quantify the bioavailable fraction rather than total concentration [47,91,131].

**Figure 9:**
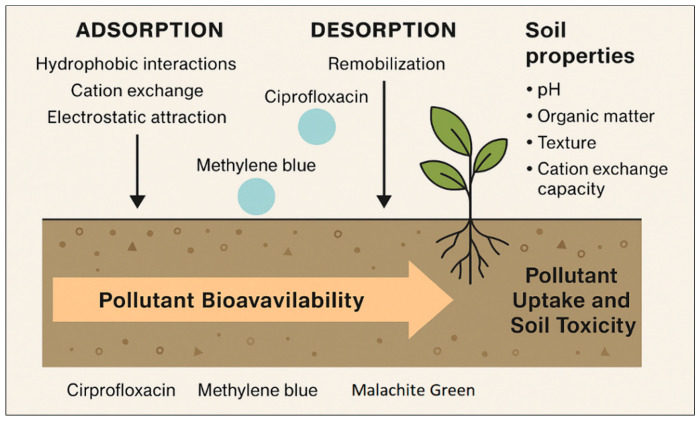
Conceptual representation of soil-mediated modulation of pharmaceutical and dye toxicity through adsorption–desorption dynamics, highlighting the roles of organic matter, pH, texture, and CEC in pollutant mobility and bioavailability.

The modulation of pollutant toxicity by soil physicochemical properties arises from dynamic adsorption–desorption equilibria that govern pollutant mobility, persistence, and plant bioavailability. These processes determine the proportion of contaminants that remain dissolved in the soil solution and thus accessible for uptake or degradation. Figure 9 schematically illustrates the principal interactions controlling pollutant behavior in soils, emphasizing the influence of organic matter, clay minerals, pH, and cation exchange capacity (CEC) on adsorption, desorption, and plant uptake pathways.

## 4. Photocatalytic Wastewater Treatment as a Source Control Strategy

### 4.1. Principles of Photocatalytic Degradation—ROS Generation, Catalyst Activation, Reaction Pathways

Photocatalytic degradation represents one of the most advanced and sustainable advanced oxidation processes (AOPs) for the removal of persistent pharmaceuticals and dyes from wastewater prior to its discharge or reuse in agriculture. The process is based on the activation of a semiconductor catalyst by light irradiation, typically ultraviolet (UV), visible, or solar, which initiates a cascade of redox reactions capable of mineralizing complex organic molecules into simpler, non-toxic products such as CO_2_, H_2_O, and inorganic ions [94,95,134,135].

The underlying mechanisms of photocatalytic degradation rely on complex photoinduced redox reactions that enable the breakdown of pharmaceuticals and dyes into less harmful or mineralized products. Upon illumination with photons of suitable energy, semiconductor photocatalysts such as TiO_2_ or ZnO undergo electronic excitation, promoting electrons from the valence band to the conduction band and leaving behind positively charged holes. These charge carriers migrate to the catalyst surface, where they trigger a cascade of oxidative and reductive reactions with adsorbed molecules.

**Figure 10:**
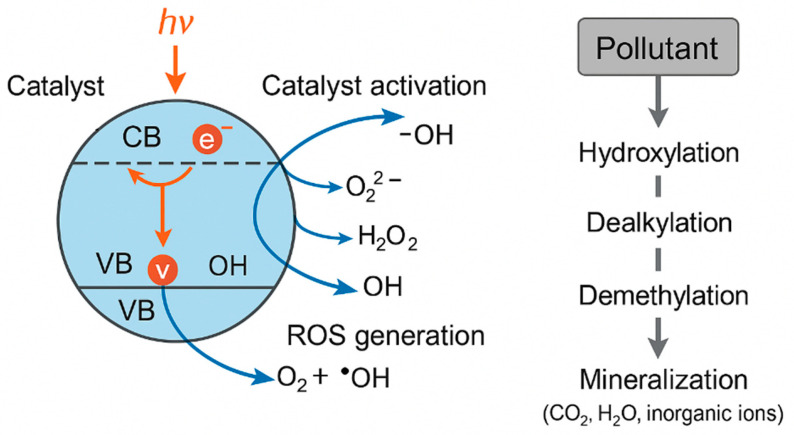
Mechanistic representation of the photocatalytic degradation of pharmaceuticals and dyes, showing catalyst activation, ROS generation, and oxidative pathways leading to pollutant mineralization.

As shown in Figure 10, this process initiates the generation of highly reactive oxygen species (ROS), including hydroxyl radicals (•OH), superoxide anions (O_2_•^−^), and hydrogen peroxide (H_2_O_2_), through interactions with water and molecular oxygen. The radicals attack the aromatic and heterocyclic structures of pharmaceutical and dye pollutants, leading to hydroxylation, dealkylation, demethylation, and eventual ring cleavage. Continuous oxidation results in the progressive mineralization of pollutants into carbon dioxide, water, and inorganic ions.

Figure 10 highlights each mechanistic stage: light absorption, charge separation, ROS generation, surface oxidation, and pollutant degradation, illustrating how photocatalytic processes transform persistent organic contaminants into environmentally benign products.

#### 4.1.1. Catalyst Activation and Electron–Hole Generation

Upon illumination with photons of energy equal to or greater than the semiconductor band gap, an electron (e^−^) is excited from the valence band (VB) to the conduction band (CB), leaving behind a positively charged hole (h^+^). This fundamental photoexcitation event produces highly reactive charge carriers that serve as the driving force for oxidation–reduction reactions at the catalyst surface. Widely studied photocatalysts include TiO_2_, ZnO, g–C_3_N_4_, and doped composites incorporating metals or carbonaceous materials, which extend light absorption into the visible spectrum and enhance charge separation efficiency [62,96,102,136].

#### 4.1.2. ROS Generation and Reactive Pathways

The photogenerated holes (h^+^) oxidize adsorbed water (H_2_O) or hydroxide ions (OH^−^) to form hydroxyl radicals (•OH), while the excited electrons (e^−^) reduce dissolved oxygen (O_2_) to superoxide radicals (O_2_•^−^). These species, together with other derived intermediates such as hydrogen peroxide (H_2_O_2_) and singlet oxygen (^1^O_2_), constitute a network of reactive oxygen species (ROS) capable of non-selective oxidation of pollutants. The overall mechanism can be summarized as follows:

- Catalyst excitation:

Catalyst + hν → e^−^ + h^+^

- Oxidation reactions:

h^+^ + H_2_O/OH^−^ → •OH

- Reduction reactions:

e^−^ + O_2_ → O_2_•^−^ → H_2_O_2_ → •OH

The •OH radical is the dominant oxidative species, possessing a redox potential of +2.8 V, enabling it to break aromatic rings, cleave dye chromophores, and attack pharmaceutical molecular backbones [95,135].

#### 4.1.3. Degradation Pathways of Pharmaceuticals and Dyes

Photocatalytic degradation of pharmaceuticals (e.g., sulfamethoxazole, carbamazepine, diclofenac) and dyes (e.g., methylene blue, malachite green, rhodamine B) proceeds through successive hydroxylation, dealkylation, demethylation, and ring-opening reactions, leading to the formation of oxygenated intermediates and eventual mineralization. The process often follows Langmuir–Hinshelwood kinetics, where adsorption of the pollutant onto the catalyst surface precedes photooxidation [62,102]. However, incomplete degradation can generate transformation products that differ in polarity, toxicity, and soil mobility, underscoring the need for full characterization of treated effluents prior to reuse [97,137].

#### 4.1.4. Photocatalyst Stability and Reusability

An essential criterion for practical implementation is the stability and recyclability of the photocatalyst. Repeated photoactivation cycles can induce surface fouling, particle agglomeration, or loss of active sites due to the deposition of degradation by–products. Strategies such as doping, surface modification, and immobilization on inert supports (glass, zeolite, biochar) are increasingly applied to improve recovery, minimize leaching, and maintain catalytic activity over multiple cycles [96,136].

Overall, photocatalytic degradation transforms wastewater from a contamination source into a treated resource with significantly reduced ecotoxicological impact. Its integration into agricultural water reuse systems offers a promising approach to prevent soil and plant exposure to persistent organic pollutants while aligning with circular economy and sustainability goals.

### 4.2. Photocatalysts and Operational Conditions

The efficiency of photocatalytic degradation processes strongly depends on the type of catalyst, its structural and electronic properties, and the operational parameters under which the process is conducted. Optimizing these factors determines the rate of reactive oxygen species (ROS) formation, the selectivity toward degradation pathways, and the mineralization efficiency of pharmaceuticals and dyes before wastewater reuse in agriculture.

#### 4.2.1. TiO_2_-Based Catalysts

Titanium dioxide (TiO_2_) remains the most extensively studied photocatalyst due to its chemical stability, non-toxicity, and strong oxidative potential. Among its crystalline polymorphs, anatase exhibits the highest photocatalytic activity owing to its superior electron mobility and higher density of surface hydroxyl groups. However, TiO_2_ has a wide band gap (~3.2 eV), limiting activation to UV wavelengths that constitute less than 5% of solar radiation [95,97,135,137]. To overcome this limitation, doping with metal or non-metal elements (e.g., Fe, N, C, S) and forming heterojunctions with conductive carbon materials or narrow-bandgap semiconductors (e.g., g-C_3_N_4_, CdS, BiVO_4_) have been effective in extending light absorption into the visible spectrum and suppressing charge recombination [8,96,136].

#### 4.2.2. ZnO Photocatalysts

Zinc oxide (ZnO) is another widely applied semiconductor owing to its similar band gap (3.2–3.3 eV) and conduction band potential close to that of TiO_2_, but with the advantage of higher quantum efficiency under UV illumination [62,102].

ZnO exhibits high photonic responsiveness and cost-effectiveness, making it suitable for large–scale applications. However, its photocorrosion under prolonged irradiation and high-pH conditions can reduce stability. Composite systems, such as ZnO–TiO_2_, ZnO–graphene, or ZnO doped with transition metals (e.g., Mn, Fe, Cu), have demonstrated improved durability, broader light absorption, and enhanced ROS generation efficiency [98,138].

#### 4.2.3. g-C_3_N_4_ and Visible-Light Photocatalysis

Graphitic carbon nitride (g–C_3_N_4_) represents a new generation of metal-free visible-light photocatalysts with a moderate band gap (~2.7 eV) and high chemical stability. Its layered structure facilitates charge transport, while the presence of nitrogen-rich functional groups enables strong interaction with pollutants and co-catalysts [96,136].

Nevertheless, pristine g–C_3_N_4_ suffers from limited surface area and rapid electron–hole recombination. These limitations are mitigated through nanostructuring, doping, and heterojunction formation with materials such as TiO_2_, ZnO, or metal–organic frameworks (MOFs), which enhance visible-light absorption and interfacial charge transfer [97,137].

#### 4.2.4. Doped and Hybrid Photocatalysts

Doping and hybridization strategies aim to modulate the band structure, narrow the band gap, and promote efficient charge separation. Metal doping (Fe^3+^, Cu^2+^, Ag^+^) introduces new energy levels that facilitate visible-light utilization, while non-metal doping (N, S, C, B) improves surface hydrophilicity and oxygen adsorption.

Hybrid systems combining semiconductors, carbon-based materials (graphene, biochar), or plasmonic nanoparticles create Z-scheme heterojunctions, maintaining high redox potentials while extending photon absorption to the visible and near-infrared regions [4,62,102]. These engineered catalysts are particularly valuable in solar-driven wastewater treatment systems designed for agricultural reuse.

#### 4.2.5. Operational Conditions

Operational parameters, including light source, pollutant concentration, pH, catalyst dosage, aeration, and reaction time strongly influence photocatalytic performance. UV systems typically ensure rapid activation of TiO_2_ and ZnO, while visible and solar systems require doped or hybrid catalysts to maintain efficiency. Neutral to slightly alkaline pH often favors ROS formation and pollutant adsorption, though pH–specific ionization states of pharmaceuticals can modulate degradation kinetics [97,137].

Aeration enhances oxygen availability for O_2_•^−^ formation, while optimal catalyst dosage prevents light scattering and aggregation effects. Increasingly, solar-driven photocatalysis is being explored as a low-energy, sustainable option compatible with decentralized wastewater treatment units for irrigation reuse [31,37].

In summary, selecting an appropriate catalyst system and tuning operational conditions are fundamental to achieving efficient photocatalytic degradation of pharmaceuticals and dyes. Advances in doped and hybrid semiconductors now enable visible-light operation, improved reusability, and compatibility with solar systems, thereby supporting integrated wastewater management strategies that minimize pollutant transfer to soil and plant systems.

### 4.3. Performance Metrics—Degradation Rates, Mineralization, and Reduction in Toxicity

The evaluation of photocatalytic treatment performance extends beyond the simple disappearance of pollutants from solution, requiring a comprehensive assessment of kinetic efficiency, mineralization extent, and ecotoxicity reduction. These metrics collectively determine whether photocatalytic degradation effectively transforms pharmaceuticals and dyes into environmentally safe products suitable for agricultural water reuse.

#### 4.3.1. Degradation Rates and Kinetic Models

Degradation kinetics are commonly modeled using pseudo-first-order or Langmuir–Hinshelwood (L–H) expressions, reflecting both surface adsorption and photochemical reaction dynamics. The apparent rate constant (*kₐₚₚ*) is obtained from the linear relationship between pollutant concentration and irradiation time, allowing for comparison between catalysts and operating conditions. Reported *kₐₚₚ* values for pharmaceutical degradation under UV or solar photocatalysis typically range from 0.01 to 0.25 min^−1^, depending on light intensity, catalyst dosage, and pollutant structure [62,95,102,135].

Dyes such as methylene blue, malachite green, and rhodamine B are often degraded more rapidly (*kₐₚₚ* ≈ 0.15–0.50 min^−1^) due to their strong light absorption and high affinity for catalyst surfaces [42,83]. Enhanced kinetics have been achieved in doped and heterojunction systems (e.g., TiO_2_–g–C_3_N_4_, ZnO–graphene), where efficient charge separation and visible-light activation accelerate ROS formation and pollutant oxidation.

#### 4.3.2. Mineralization Efficiency

Complete removal of parent molecules does not necessarily ensure environmental safety, as partially oxidized intermediates may persist and exhibit biological activity. Therefore, mineralization, quantified by total organic carbon (TOC) or chemical oxygen demand (COD) reduction, serves as a more robust indicator of photocatalytic efficacy. TOC removals of 70–95% are frequently reported for pharmaceuticals such as sulfamethoxazole, diclofenac, and carbamazepine after 120–180 min of solar-assisted photocatalysis [96,97,136,137].

Similarly, near-complete decolorization of dyes often precedes full mineralization, reflecting the multi-step degradation pathway involving chromophore cleavage, aromatic ring opening, and eventual conversion to CO_2_ and H_2_O. Incomplete mineralization can yield short-chain acids, aldehydes, or quinone-like species that must be characterized to assess treatment completeness and avoid secondary pollution [4].

#### 4.3.3. Photocatalytic Mineralization Versus Partial Degradation

Although photocatalysis is often conceptualized as a complete mineralization process leading to the conversion of organic pollutants into CO_2_, H_2_O, and inorganic ions, total mineralization is rarely achieved under practical conditions. Instead, photocatalytic systems usually drive partial oxidation, producing transformation products (TPs) that may persist and, in some cases, exhibit higher mobility or toxicity than their parent compounds [86,90,126,130].

The degree of mineralization is governed by multiple interrelated factors, including catalyst composition, light intensity, reaction time, and pollutant structure. Under optimal laboratory conditions, many photocatalysts such as TiO_2_, ZnO, and g–C_3_N_4_ achieve > 90% degradation of pharmaceuticals and dyes, yet their total organic carbon (TOC) removal often remains limited to 60–85%, indicating incomplete oxidation [95,97,135,136]. The discrepancy arises because the generation of reactive oxygen species (ROS), particularly hydroxyl radicals (•OH) and superoxide radicals (O_2_•^−^) is kinetically sufficient to break aromatic rings and functional groups, but mineralization (the ultimate conversion to CO_2_) proceeds more slowly, especially when intermediates are strongly adsorbed or stabilized on the catalyst surface [62,102].

During photocatalysis, pollutants typically undergo sequential hydroxylation, dealkylation, demethylation, nitration, and aromatic ring cleavage reactions, yielding hydroxylated, carboxylated, and quinone-like intermediates. These TPs have been documented for several recalcitrant compounds, including carbamazepine, which transforms into 10,11-epoxycarbamazepine and acridone, and diclofenac, which produces 4′-hydroxydiclofenac and 2,6-dichloroaniline under TiO_2_ and ZnO photocatalysis [95,97,135,137]. For azo and triarylmethane dyes such as methylene blue and malachite green, N-demethylated and leuco derivatives are frequently observed prior to full decolorization, indicating incomplete ring mineralization [99,139].

The persistence of TPs in treated effluents has been confirmed in both bench-scale and pilot-scale systems, where TOC removal rarely matches the near-total disappearance of the parent compound. These intermediates can resist further oxidation due to steric hindrance, halogen substitution, or aromatic resonance stabilization, slowing down mineralization kinetics [4]. Furthermore, interactions between TPs and catalyst surfaces can induce adsorption–desorption equilibria, temporarily protecting certain molecules from further ROS attack [86,126].

From an environmental standpoint, the generation of TPs represents a double-edged outcome: while photocatalysis successfully reduces the concentration of the parent pollutant, it may yield compounds that remain chemically reactive, bioavailable, and ecotoxic [85,100,125,140]. These oxidation by–products can interact with soil organic matter and microbial communities when effluents are reused for irrigation, emphasizing the importance of coupling chemical monitoring (e.g., LC–MS/MS, TOC) with bioassays to evaluate the full extent of detoxification. Thus, while photocatalysis is an essential component of advanced wastewater treatment, complete mineralization remains the exception rather than the rule under real-world operational conditions.

Table 5 illustrates that photocatalytic degradation is highly effective in decomposing pharmaceuticals and dyes at the molecular level, yet total organic carbon (TOC) removal rarely exceeds 85%. The partial oxidation process results in stable, oxygenated intermediates such as hydroxylated aromatics, carboxylic acids, and quinones. These transformation products often persist in treated water and exhibit distinct physicochemical and biological reactivities compared with their parent molecules. Therefore, assessing both degradation and mineralization endpoints is critical to avoid underestimating environmental risks associated with the reuse of photocatalytically treated wastewater.

#### 4.3.4. Reduction in Toxicity and Ecological Safety

A critical performance metric, especially for water intended for agricultural reuse, is the reduction in residual ecotoxicity following photocatalytic treatment. Studies integrating bioassays with degradation tests consistently show significant reductions in phytotoxicity, microbial inhibition, and aquatic organism mortality after photocatalysis. Solar-driven and photocatalytic advanced oxidation processes have been widely evaluated for both pollutant degradation and ecotoxicological safety, often using aquatic primary producers as indicators of detoxification. In the case of phenolic contaminants, Herrera-Melián et al. (2012) demonstrated that solar TiO_2_-photocatalysis, combined with photo-Fenton processes, achieved substantial mineralization of 4-nitrophenol, with total organic carbon reductions approaching 80% after 180 min, and the treated effluents exhibited negative growth inhibition of *Lemna minor*, confirming effective detoxification [101,141]. Similar observations of toxicity reduction toward *Lemna minor* have been reported for non-photocatalytic remediation systems treating real dye effluents. For example, fungal biosorption significantly decreased the toxicity of textile wastewater after colour removal, indicating that dye degradation corresponded to genuine detoxification. In shallow-pond microcosms, Yaseen and Scholz (2016) showed that *Lemna minor* enhanced the removal of azo dyes and COD, producing treated effluents with physicochemical characteristics that met environmental discharge requirements [102,142].

Beyond plant-based toxicity assays, photocatalytic processes have also been examined using microalgae. Baran et al. (2006) demonstrated that TiO_2_–mediated photocatalysis of sulfonamide antibiotics resulted in marked toxicity reductions toward the green alga *Chlorella vulgaris*, with degradation products showing significantly lower inhibitory effects than the parent compounds while biodegradability increased over irradiation time [103,143]. Additional photocatalytic studies involving TiO_2_ nanoparticles confirmed the relevance of algal assays for assessing both catalyst-related effects and pollutant degradation behaviour, as seen in the aquatic toxicity evaluation of imidacloprid and TiO_2_ carried out by Andronic et al. (2021) [104,144].

By contrast, when *Chlorella vulgaris* is used directly as a bioremediation agent rather than as a toxicity indicator, the removal efficiencies differ considerably. In high-rate algal pond systems, Lim et al. (2010) reported moderate colour removal (≈42–50%) and substantial reductions in nutrients and COD during the treatment of textile wastewater, driven largely by biosorption mechanisms [105,145].

The toxicity decline correlates with progressive aromatic ring oxidation and mineralization, indicating the conversion of bioactive molecules into less harmful or inert species. However, temporary increases in toxicity have been observed during intermediate formation, underscoring the need for time-resolved toxicity assessment.

#### 4.3.5. Integrated Performance Assessment

An integrated evaluation of photocatalytic performance thus combines kinetic (degradation rate), stoichiometric (mineralization), and biological (toxicity reduction) metrics. This approach allows for more accurate determination of environmental benefits and informs scale-up strategies for real wastewater systems. Benchmarking across catalysts reveals that while UV-driven TiO_2_ remains a performance standard, visible-light-activated materials such as g-C_3_N_4_ heterojunctions now achieve comparable removal and detoxification under solar conditions with markedly lower energy input [62,102]. Future system optimization should balance degradation rate enhancement with long-term catalyst stability, transformation product management, and assurance of ecological safety before agricultural reuse.

**Figure 11:**
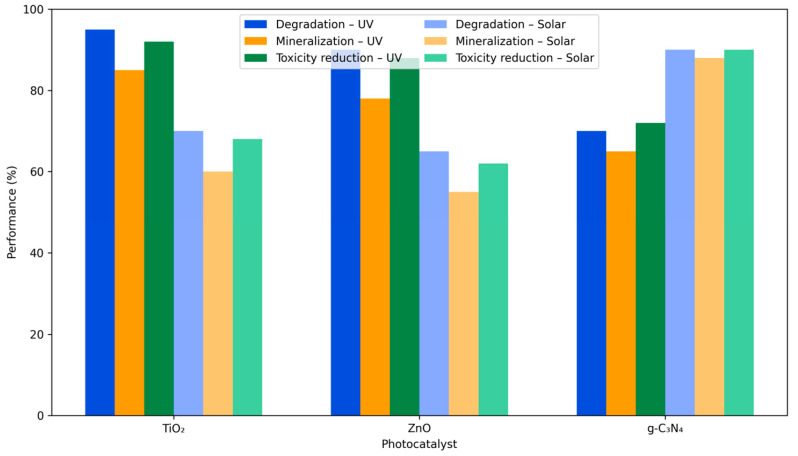
Comparative photocatalytic performance of TiO_2_, ZnO, and g-C_3_N_4_ under UV and solar irradiation. Bars represent degradation efficiency, mineralization (TOC removal), and toxicity reduction. Solid bars correspond to UV irradiation, while light-shaded bars indicate solar irradiation. Data highlight the benchmark UV performance of TiO_2_ and the enhanced visible-light activity of g-C_3_N_4_-based systems [107,148].

The comparative performance of different photocatalysts under UV and solar irradiation provides essential insights into their efficiency, sustainability, and suitability for wastewater treatment applications targeting pharmaceutical and dye pollutants. As summarized in Figure 11, the three representative materials: TiO_2_, ZnO, and g-C_3_N_4_ exhibit distinct activity profiles reflecting their electronic structures and light-harvesting capabilities. Under UV irradiation, TiO_2_ demonstrates the highest overall performance, achieving degradation efficiencies near 95%, mineralization rates of approximately 85%, and toxicity reductions exceeding 90%, confirming its role as a benchmark catalyst for laboratory-scale systems [106,146].

ZnO displays comparable behavior with slightly lower mineralization efficiency due to partial photocorrosion during prolonged irradiation. Conversely, g–C_3_N_4_ shows moderate UV activity but superior solar-driven performance, reaching nearly 90% degradation and 88% mineralization with notable detoxification efficiency (~90%). These results highlight the increasing relevance of g–C_3_N_4_ and doped composite photocatalysts for solar-assisted processes, which combine visible-light activation with reduced energy input and improved environmental compatibility. The comparative data emphasize that while TiO_2_ remains the most robust catalyst under UV conditions, g–C_3_N_4_–based systems are emerging as the most promising materials for scalable, solar-powered wastewater treatment solutions that align with sustainable agriculture and circular economy principles [108,147].

### 4.4. Transformation Product Formation—Mechanisms, Persistence, and Implications for Soil–Plant Exposure

Although photocatalytic treatment significantly enhances the degradation of pharmaceuticals and dyes, the process rarely leads to instantaneous mineralization. Instead, it proceeds through a series of intermediate reactions that generate transformation products (TPs), as new chemical entities derived from partial oxidation, reduction, or rearrangement of parent compounds. These intermediates may differ in polarity, stability, and biological activity, and can substantially influence the environmental safety of treated wastewater when applied to agricultural soils. Understanding the mechanistic pathways, persistence characteristics, and ecotoxicological implications of these TPs is therefore essential to fully assess the benefits and risks of photocatalytic wastewater reuse [109,110,149,150].

The characterization of photocatalytic systems based on their efficiency, degradation pathways, and transformation products (TPs) provides critical insight into the environmental implications of advanced oxidation processes for drug and dye removal. Although photocatalysis effectively reduces pollutant concentrations, the process often generates intermediate compounds that vary widely in structure, persistence, and ecotoxicity. These intermediates may retain bioactivity or form new reactive moieties capable of interacting with soil–plant and microbial systems after treated wastewater reuse.

Table 6 summarizes representative photocatalytic systems applied for the degradation of pharmaceuticals and synthetic dyes under UV and solar irradiation, highlighting the main catalysts, operational conditions, identified transformation products, and corresponding mechanistic findings. Semiconductor catalysts such as TiO_2_, ZnO, and g-C_3_N_4_ dominate current research due to their tunable band gaps, stability, and capacity for reactive oxygen species (ROS) generation. However, the Table reveals that even highly efficient systems (e.g., TiO_2_–g–C_3_N_4_ or TiO_2_–Ag hybrids) produce multiple intermediate species, such as hydroxylated or N-demethylated derivatives, aromatic amines, and low-molecular-weight carboxylic acids before achieving complete mineralization. These results underscore the dual role of photocatalysis as both a powerful pollutant degradation tool and a potential source of secondary contamination if transformation products persist in effluents used for agricultural irrigation.

#### 4.4.1. Mechanistic Pathways of Transformation Product Formation

The generation of TPs during photocatalytic degradation results from sequential radical-mediated reactions involving reactive oxygen species (ROS), including hydroxyl radicals (•OH), superoxide anions (O_2_•^−^), and singlet oxygen (^1^O_2_). These radicals attack molecular sites with high electron density, such as aromatic rings, heterocyclic nitrogen atoms, and unsaturated bonds, initiating hydroxylation, dealkylation, demethylation, nitration, and ring-opening reactions [112,152].

For instance, the antibiotic sulfamethoxazole (SMX) undergoes successive oxidation of the aniline moiety and cleavage of the sulfonamide bond, yielding intermediates such as 3-amino-5-methylisoxazole, sulfanilic acid, and N^4^–acetyl-SMX [85,86,125,126]. Similarly, the antiepileptic drug carbamazepine (CBZ) forms hydroxylated and epoxide derivatives (e.g., 10,11–epoxy–CBZ) before eventual ring-opening and mineralization [97,137]. In the case of synthetic dyes, photocatalytic cleavage of the azo (–N=N–) or anthraquinone chromophore produces aromatic amines and quinone intermediates that may transiently persist in treated effluents [87,90,127,130].

Each of these transformation steps is influenced by the nature of the catalyst, light spectrum, oxidant availability, and reaction pH, which together control the oxidation potential and pathway selectivity. For example, visible-light-active catalysts such as g-C_3_N_4_/TiO_2_ composites tend to favor hydroxylation and oxygenation routes over deep oxidation, often leading to more hydrophilic TPs that remain soluble and potentially mobile in soil–water systems [62,102].

#### 4.4.2. Persistence and Environmental Behavior of Transformation Products

While photocatalysis effectively degrades parent molecules, many TPs exhibit greater persistence in the environment due to enhanced polarity, reduced volatility, or decreased reactivity with radicals. The formation of stable carboxylic acids, phenolic derivatives, and nitroaromatic compounds has been observed following the photocatalytic degradation of antibiotics and dyes under both UV and solar irradiation [153,154]. Such TPs often resist further photolysis and microbial mineralization once discharged into natural waters or applied to soils through irrigation.

Persistence is also influenced by sorption–desorption dynamics in soils. More polar TPs typically exhibit weaker adsorption to organic matter and clay minerals, increasing their mobility and leaching potential, while less polar or aromatic intermediates may strongly sorb to soil particles, contributing to long-term accumulation [61]. For example, oxidation intermediates of CBZ and SMX have been detected in agricultural soils months after wastewater application, indicating limited biodegradability and potential chronic exposure of plants and soil microbiota [85,87,125,127].

#### 4.4.3. Implications for Soil–Plant Systems and Ecotoxicological Risk

Transformation products introduce a complex layer of ecotoxicological uncertainty, as their behavior and biological effects are not always predictable from the parent compound’s structure. In soil–plant systems, TPs can enter the rhizosphere through infiltration of treated wastewater or biosolid application, subsequently interacting with soil biota or being taken up by plant roots. Some TPs are known to retain antimicrobial, genotoxic, or oxidative properties, thereby disrupting root-associated microbial communities, altering enzyme activities, and affecting nutrient cycling [93,133].

Plant uptake studies have confirmed that TPs such as hydroxylated CBZ derivatives and sulfonamide oxidation products can be translocated within plant tissues, accumulating in leaves and edible parts. This occurs primarily for polar, low-molecular-weight compounds that remain bioavailable in soil pore water [33,39]. Additionally, TPs of dyes, particularly aromatic amines from azo bond cleavage, may induce oxidative stress and pigment disruption in plant tissues when present in irrigation water [127]. These findings highlight that even when photocatalytic treatment reduces the apparent pollutant load, incomplete mineralization may lead to secondary risks through persistent by-products.

#### 4.4.4. Strategies for TP Management and Risk Mitigation

To minimize TP formation and persistence, recent research emphasizes process optimization and post-treatment integration. Combining photocatalysis with biological polishing steps (e.g., biofiltration or constructed wetlands) facilitates the biodegradation of partially oxidized intermediates, reducing ecotoxicity [62,102]. Furthermore, reactor design improvements, such as controlled irradiation wavelength, photocatalyst immobilization, and reaction time optimization help balance efficient degradation with minimal TP accumulation. Analytical advances including high–resolution mass spectrometry (HRMS) and suspect/non-target screening now enable more comprehensive TP identification, improving environmental risk assessments for treated effluents [41,75,82,95,115,135].

**Figure 12:**
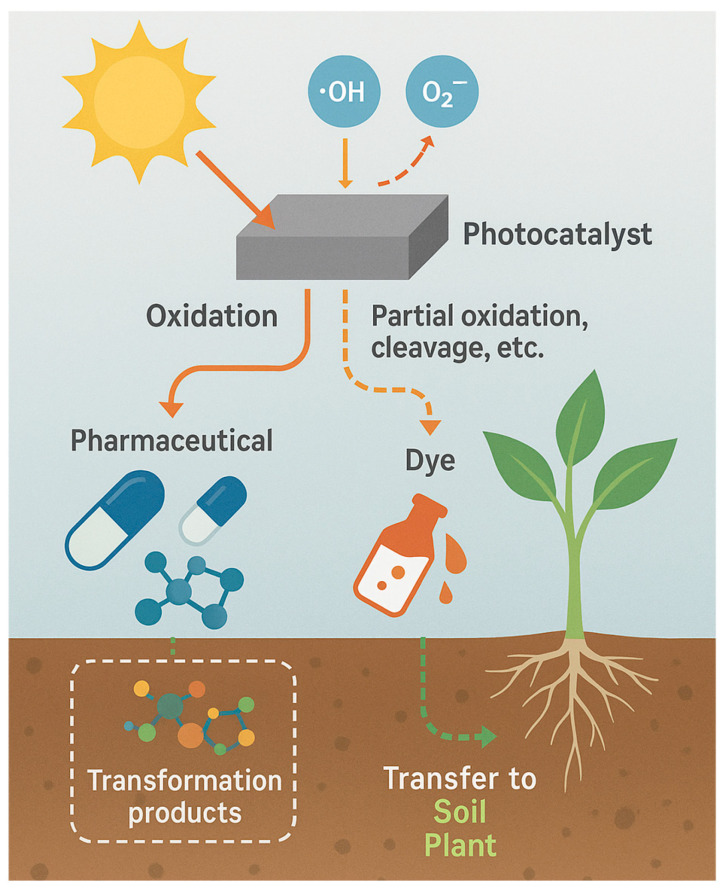
Schematic representation of transformation product formation during photocatalytic degradation of pharmaceuticals and dyes, showing ROS generation, intermediate formation, and subsequent entry of transformation products into soil and plant systems through irrigation and root uptake.

Overall, the formation of transformation products (TPs) during photocatalytic degradation represents a critical stage that bridges pollutant removal and environmental safety assessment. These intermediates, often produced through partial oxidation, hydroxylation, dealkylation, or chromophore cleavage, can persist in treated wastewater and subsequently enter soil and plant systems upon agricultural reuse. As illustrated in Figure 11, the photocatalytic process begins with the irradiation of semiconductors such as TiO_2_, ZnO, or g-C_3_N_4_ under UV or solar light, generating reactive oxygen species (ROS) that attack pharmaceuticals and dyes. This initiates a cascade of chemical transformations, producing hydroxylated, carboxylated, or amine derivatives that exhibit altered polarity, bioavailability, and toxicity. These transformation products can migrate with irrigation water into the soil–plant continuum, where they may adsorb to mineral or organic fractions, leach through soil layers, or be absorbed by plant roots and translocated to aerial tissues. Figure 12 summarizes this interconnected process, from ROS-driven oxidation to environmental dissemination emphasizing the need to evaluate not only the degradation efficiency but also the persistence and ecological impact of the resulting transformation products.

Ultimately, recognizing transformation products as integral endpoints rather than side effects of photocatalysis is essential to ensure safe wastewater reuse. A holistic evaluation combining chemical analysis, bioassays, and plant–soil exposure tests should be integrated into treatment design to verify that photocatalytic systems truly deliver ecotoxicological neutrality and prevent pollutant transfer into terrestrial food chains.

### 4.5. Integration of Monitoring Tools and Risk Modeling Metrics (PEC, PNEC, HQ) in AOP Evaluation

Monitoring frameworks and predictive modeling tools are increasingly essential for evaluating advanced oxidation processes, particularly when transformation products (TPs) rather than parent compounds are the main contributors to environmental hazard. In contemporary assessments, comprehensive analytical monitoring is paired with risk-based modeling metrics to establish a scientifically grounded basis for selecting and optimizing photocatalytic technologies.

Advances in LC–MS/MS and high-resolution mass spectrometry (HRMS) now allow for simultaneous detection of parent pollutants and dozens of TPs, including transient quinone-imines, halogenated aromatics, and nitrogen-containing intermediates. Recent studies have demonstrated the capacity of non-target HRMS workflows, supported by molecular networking and in silico prediction, to identify >50 previously unreported TPs formed during photocatalysis or photo-Fenton processes [155,156]. These analytical capabilities significantly improve TP characterization, enabling the integration of toxicity-driven prioritization in AOP evaluation.

Risk modeling approaches such as Predicted Environmental Concentrations (PEC), Predicted No-Effect Concentrations (PNEC), and derived Hazard Quotients (HQ = PEC/PNEC) provide quantitative indicators of ecological impact for both parent compounds and their TPs. Regulatory bodies, including the European Chemicals Agency (ECHA) and European Water Framework Directive (WFD), increasingly emphasize HQ-based risk screening, especially for pharmaceuticals and dyes detected in agricultural reuse systems. Several pharmaceuticals included on the EU Watch List, such as diclofenac and sulfamethoxazole, routinely exhibit HQ values > 1, indicating significant risk under typical wastewater reuse scenarios [68,157]. Recent environmental monitoring confirms that certain TPs exceed risk thresholds more frequently than their parent compounds. For example: Diclofenac-quinone-imine has been detected in treated effluents at 0.02–0.08 µg L^−1^, yielding HQ values of 4–16, exceeding those calculated for diclofenac itself [158,159]. Halogenated anilines produced from diclofenac or other chlorinated pharmaceuticals were observed at 0.005–0.03 µg L^−1^ in reclaimed water, with PNEC values as low as 0.001 µg L^−1^ yielding HQ > 5 [160,161]. Acridone and acridine derivatives from carbamazepine photocatalysis exhibit PNECs of 0.003–0.01 µg L^−1^ and measurable PECs of 0.005–0.02 µg L^−1^, often giving HQ values exceeding 1 [162,163].

Leucomalachite green, a dominant TP of malachite green, persists at 0.005–0.05 µg L^−1^ in agricultural pond systems and exhibits PNECs below 0.0001 µg L^−1^ due to strong mutagenicity, resulting in HQ values between 50 and 500 [163,164]. These values highlight the central point that TPs can be the principal drivers of ecological risk, particularly when photocatalytic degradation is incomplete or when matrix components (e.g., chloride, nitrate, dissolved organic carbon) promote formation of highly stable TP families.

Incorporating PEC/PNEC modeling during process development enables the identification of hazard-driving intermediates, including quinone-imines, halogenated anilines, and benzophenone-type carbonyls, whose formation risks may outweigh the benefits of parent-compound degradation. Such modeling is increasingly supported by probabilistic risk assessment (PRA) tools, which integrate temporal concentration profiles, TP formation kinetics, and exposure frequency in agricultural reuse systems [165].

Embedding these metrics early in the evaluation framework strengthens conceptual continuity between analytical identification, mechanistic interpretation, and AOP optimization. This integration ensures that photocatalytic systems are selected not only for degradation efficiency but also for their capacity to suppress or avoid formation of persistent, bioaccumulative, or toxicologically potent TPs. Table 7 synthesizes representative PEC, PNEC, and HQ values for widely occurring micropollutants and their high-impact TPs, illustrating how risk-based modeling identifies intermediates that may drive ecological hazard even when parent-compound concentrations decline substantially during photocatalysis.

## 5. Post–Photocatalytic Residuals: Transformation Products and Soil–Plant Toxicity

### 5.1. Classification of Transformation Product (TP) Types

Photocatalytic degradation of pharmaceuticals and dyes rarely proceeds directly to CO_2_ and inorganic ions. Instead, pollutants follow sequences of oxidation, reduction, and rearrangement steps that generate families of transformation products (TPs) with distinct physicochemical properties and toxicological profiles [4,102,135,137,149,150]. These TPs differ from their parent compounds in polarity, charge, aromaticity, and reactivity, which control their sorption to soil constituents, mobility in the soil–plant continuum, and uptake by roots or foliar tissues. Based on current evidence for photocatalytic treatment of pharmaceuticals and dyes, the most frequently reported TP types include: (i) hydroxylated and dealkylated derivatives, (ii) ring-cleavage products and short-chain carboxylic acids, (iii) aromatic amines and related nitrogen-containing species, (iv) halogenated and nitrated intermediates, and (v) quinone-type and other carbonyl compounds [126,130,135,139,151].

The following sub-subsections summarize the main structural features, formation patterns, and environmental implications of these TP classes, with emphasis on systems relevant for wastewater reuse in agriculture.

#### 5.1.1. Hydroxylated and Dealkylated Derivatives

Hydroxylated TPs represent one of the most common product families during photocatalytic oxidation. Attack by hydroxyl radicals (·OH) on aromatic rings or aliphatic substituents generates mono- and poly-hydroxylated derivatives and promotes stepwise dealkylation or demethylation [4,102]. For sulfonamide antibiotics such as sulfamethoxazole, identified TPs include N^4^-acetyl-SMX, 3-amino-5-methylisoxazole, sulfanilic acid, and short-chain carboxylic acids, all formed through sequential hydroxylation and cleavage of the sulfonamide linkage [126]. For carbamazepine, photocatalytic and solar-assisted systems frequently yield 10,11-epoxycarbamazepine, 2-hydroxycarbamazepine, and acridone as primary and secondary intermediates [135,137,151]. Hydroxylation generally increases polarity and reduces log Kₒw, which enhances solubility and potential mobility in soil pore water. However, these derivatives retain aromatic structures and often preserve biological activity; so, toxicity reduction is not guaranteed [125,126,135]. In plant–soil systems, more polar hydroxylated TPs can more easily reach the rhizosphere, cross root membranes, and translocate to shoots compared with their more hydrophobic parents.

**Figure 13:**
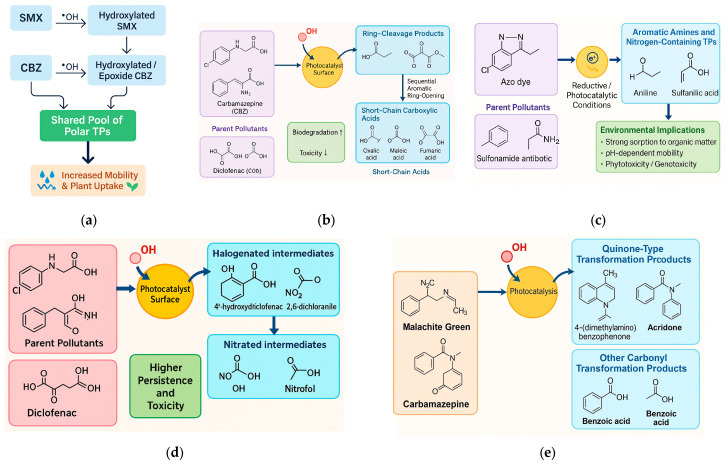
Overview of major transformation product (TP) pathways occurring during photocatalytic oxidation of pharmaceuticals and dyes: (**a**) Conceptual pathway of hydroxylation and dealkylation during photocatalytic oxidation; (**b**) Formation of ring-cleavage products and short-chain carboxylic acids during photocatalytic oxidation; (**c**) Azo dye cleavage and sulfonamide transformation generate aniline-type nitrogen-containing transformation products (TPs) with strong sorption behavior, pH-dependent mobility, and potential phytotoxic and genotoxic effects; (**d**) Formation pathways of halogenated and nitrated transformation products (TPs) during photocatalytic degradation, highlighting key intermediates and their increased persistence and toxicity compared with the parent compound; (**e**) Quinone-type and other carbonyl transformation products formed during photocatalytic oxidation of aromatic pharmaceuticals and dyes, highlighting key intermediates and their relevance for plant–soil ecotoxicity.

To clarify the underlying mechanistic routes through which hydroxylated and dealkylated derivatives arise during photocatalytic oxidation, Figure 13a provides a conceptual overview of the dominant transformation pathways triggered by hydroxyl radical (•OH) attack. The illustration highlights how parent compounds such as sulfamethoxazole (SMX) and carbamazepine (CBZ) undergo sequential hydroxylation and dealkylation steps, generating more polar transformation products (TPs) that exhibit increased solubility and mobility in soil–water systems. This schematic representation supports the discussion by integrating the key reaction steps, the types of intermediates formed, and their broader environmental relevance.

#### 5.1.2. Ring-Cleavage Products and Short-Chain Carboxylic Acids

Further oxidation of hydroxylated intermediates produces ring-opening products, including dicarboxylic and tricarboxylic acids such as oxalic, maleic, and fumaric acids [102,135,137]. During photocatalytic treatment of carbamazepine, diclofenac, and tetracycline, several studies report sequential aromatic ring cleavage and gradual conversion into low-molecular-weight carboxylic acids, which dominate at advanced mineralization stages [102,135,136].

These TPs exhibit high polarity, low sorption to soil solids, and relatively rapid biodegradation under aerobic conditions. They generally show lower ecotoxicity than parent pharmaceuticals and are often considered markers of effective detoxification [135,137]. However, incomplete conversion can leave mixtures of carboxylic acids together with more persistent aromatic intermediates; so, both classes need to be considered when treated effluents are reused for irrigation.

To illustrate the progression from early oxidative steps to advanced stages of photocatalytic mineralization, Figure 13b summarizes the formation of ring-cleavage products and low-molecular-weight carboxylic acids derived from parent pharmaceuticals such as carbamazepine and diclofenac. As hydroxylated intermediates undergo sequential aromatic ring opening, structurally simplified dicarboxylic and tricarboxylic acids, such as oxalic, maleic, and fumaric acids become dominant transformation products. The figure highlights these key oxidative routes and emphasizes the increased polarity, biodegradability, and generally reduced toxicity associated with short-chain acids formed during advanced photocatalytic processing.

#### 5.1.3. Aromatic Amines and Other Nitrogen-Containing Species

Reductive processes, particularly under low-oxygen conditions or in systems that combine photocatalysis with biological steps, generate aromatic amines and related nitrogen-containing TPs. For azo dyes, cleavage of the –N=N– bond yields anilines and other aromatic amines that often display strong mutagenic and carcinogenic potential [130,139]. For sulfonamide antibiotics, photocatalytic oxidation produces sulfanilic acid and other aniline-type intermediates, which persist in treated effluents and possess distinct sorption behavior compared with their parent molecules [126].

These aromatic amines exhibit higher basicity and frequently stronger interactions with soil organic matter and clays than the original dye or drug. They can bind to humic substances through covalent and non-covalent mechanisms, accumulate in the topsoil, or leach as protonated species depending on pH and soil composition. Toxicological evaluations indicate that such amines often retain phytotoxic, genotoxic, or endocrine-disrupting potential; so, their occurrence in photocatalytically treated water intended for irrigation represents a critical concern [125,130,140].

To visualize the key reductive pathways leading to nitrogen-containing transformation products, Figure 13c summarizes the formation of aromatic amines from two major pollutant groups: azo dyes and sulfonamide antibiotics. Under reductive or photocatalytic conditions, cleavage of the azo (–N=N–) bond and transformation of sulfonamide structures yield aniline derivatives such as aniline and sulfanilic acid, which commonly persist in treated effluents. The diagram highlights these core reaction routes and their environmental significance, emphasizing the strong sorption behavior, pH-dependent mobility, and potential phytotoxic and genotoxic effects associated with aromatic amines.

#### 5.1.4. Halogenated and Nitrated Intermediates

Halogenated and nitrated TPs form when halide ions, residual chlorine species, or nitrate/nitrite coexist with target pollutants during oxidative treatment. For halogenated NSAIDs such as diclofenac, photocatalytic degradation in the presence of chloride generates intermediates including 4′-hydroxydiclofenac, 5-hydroxydiclofenac, and 2,6-dichloroaniline [135,137]. These products show higher persistence and, in some cases, higher aquatic and plant toxicity than the parent compound due to the stabilizing effect of halogen substituents on aromatic rings [135,140].

Nitrated intermediates arise during advanced oxidation of phenolic and aromatic compounds in nitrate-containing waters through reactions involving ·OH, NO_2_·, and related species. Nitro-aromatic TPs exhibit strong electron-withdrawing character and high redox stability, which favors persistence in soil and potential accumulation in biota [4,149]. Both halogenated and nitrated TPs often display lower biodegradability and elevated genotoxicity; so, their presence in treated effluents demands careful monitoring when designing photocatalytic processes for agricultural reuse.

Figure 13d illustrates the formation of halogenated and nitrated transformation products (TPs) during photocatalytic degradation when chloride, nitrate, or nitrite ions are present. As detailed above, these reactions generate intermediates such as hydroxylated diclofenac derivatives, 2,6-dichloroaniline, and nitro-aromatic compounds, which often display increased persistence, strong electron-withdrawing character, and higher toxicity than the parent pollutant. The figure highlights these pathways and underscores the need to monitor such stable TPs when evaluating the safety of treated effluents for agricultural reuse.

#### 5.1.5. Quinone-Type and Other Carbonyl Transformation Products

Quinone-type and related carbonyl compounds represent another important TP class, particularly for aromatic pharmaceuticals and dyes. Oxidation of phenolic and anilide moieties converts them into benzoquinones, anthraquinones, benzophenones, and other carbonyl structures [102,130,151]. For example, photocatalytic treatment of carbamazepine produces acridone as a key quinone-type TP, while oxidation of malachite green yields 4-(dimethylamino)benzophenone and benzoic acid intermediates [102,130,139,151].

Quinones participate in redox cycling and generate reactive oxygen species in biological systems, which enhances oxidative stress in plants and soil microorganisms [125,130,140]. Their relatively high hydrophobicity and planar aromatic structure promote sorption to organic matter and possible bioaccumulation. Consequently, the presence of quinone-type TPs after photocatalysis has direct relevance for plant–soil ecotoxicity, even when parent compounds appear fully degraded based on concentration measurements or color removal.

Figure 13e illustrates the formation of quinone-type and other carbonyl transformation products (TPs) generated during photocatalytic oxidation of aromatic pharmaceuticals and dyes. As described above, oxidation of phenolic and anilide groups produces benzoquinones, benzophenones, acridone, and related carbonyl intermediates, such as those arising from carbamazepine and malachite green. These quinone-type TPs are environmentally relevant due to their redox-cycling potential, ROS generation, and tendency for sorption and bioaccumulation, highlighting that ecotoxicological risks may persist even when parent compounds appear fully degraded.

#### 5.1.6. Ecotoxicologically Relevant Transformation Products: Case Studies

Transformation products may display toxicological profiles that equal or surpass those of the parent compound, particularly when redox-active, halogenated, or carbonyl-containing intermediates accumulate during photocatalytic treatment. The following case studies elaborate two well-characterized systems, as diclofenac and malachite green, where experimentally validated TPs have demonstrated enhanced persistence, ROS generation capacity, or biological toxicity, underscoring the importance of evaluating TPs within plant–soil exposure scenarios.

##### Diclofenac: Formation of ROS-Generating Quinone-Imine Derivatives and Persistent Halogenated Aromatics

Diclofenac (DCF) undergoes several concurrent oxidative pathways during photocatalytic treatment, driven predominantly by •OH addition, single-electron transfer reactions, and, in chloride-containing waters, electrophilic substitution. Hydroxylation yields 4′-hydroxydiclofenac and 5-hydroxydiclofenac, both of which are prone to subsequent oxidation to quinone-imine structures [158]. These quinone-imines exhibit strong electrophilicity and participate in redox cycling, repeatedly generating H_2_O_2_ and O_2_•^−^ in biological systems. This mechanism has been directly associated with lipid peroxidation, glutathione depletion, and alterations in antioxidant enzyme activity in aquatic organisms and plant tissues (Figure 13).

In parallel, diclofenac’s dichlorinated anilide structure favors formation of 2,6-dichloroaniline and related halogenated TPs through dechlorination–rechlorination pathways observed in chloride-rich matrices [158]. These halogenated aromatics display high hydrophobicity, low biodegradability, and a substantial capacity for sorption to soil organic matter. Their persistence increases the likelihood of plant uptake and root-zone accumulation, with documented inhibition of root elongation, membrane integrity, and soil microbial respiration.

Importantly, several studies report that ecotoxicity during photocatalytic treatment does not follow parent-compound decay. For example: Kim et al. (2010) observed higher oxidative stress markers following partial DCF oxidation than with DCF alone [158]. This behavior reflects the continued presence of redox-active and halogenated TP families that are more resistant to mineralization than DCF itself [140,143,155,162,163,167]. As a result, the formation, persistence, and mobility of these intermediates are of direct relevance for agricultural reuse, where plant root tissues and soil microorganisms are primary receptors of chronic oxidative stress.

##### Malachite Green: Persistent N-Demethylated Aromatic Intermediates and Ecotoxicologically Relevant Benzophenone-Type TPs

Malachite green (MG) follows a stepwise series of N-demethylation and oxidative cleavage reactions during photocatalytic degradation. Initial pathways produce mono- and di-demethylated MG, followed by reduction to the non-chromophoric but highly persistent leucomalachite green (LMG) [66,170]. LMG has been reported to persist in soil and sediment for periods extending to several months due to its resistance to microbial metabolism and strong affinity for organic matter. Its toxicological significance includes carcinogenicity, reproductive toxicity, and bioaccumulation in aquatic species, with documented transfer across trophic levels. Further oxidation leads to carbinol intermediates and ultimately to 4-(dimethylamino)benzophenone and benzoic acid derivatives [51,52,67,171]. These benzophenone-type TPs have two properties of particular environmental relevance:

- Planar aromatic structure, enhancing sorption to humic substances and soil particles.
- Photoreactivity, enabling secondary ROS generation under sunlight, thereby prolonging oxidative pressure in exposed plant tissues.

MG and its TPs also demonstrate notable toxicity toward terrestrial plants. Srivastava et al. (2004) showed that MG and its demethylated derivatives induce chromosomal aberrations, micronucleus formation, and mitotic inhibition in root meristem cells, effects more pronounced than with the parent dye alone [164]. These findings suggest that photocatalytic MG degradation may temporarily yield an intermediate mixture with greater genotoxic potential than the starting compound. Furthermore, color loss is not a reliable indicator of detoxification. Some researchers documented increased acute toxicity during intermediate formation despite significant decolorization, highlighting the need for TP-focused monitoring [67,164,168,169].

Given MG’s strong sorption behavior and the hydrophobicity of its intermediates, these compounds may accumulate in agriculturally amended soils, affecting root systems, seed germination, and rhizosphere microbial communities.

##### Environmental Implications

The extended case studies clearly illustrate that photocatalytic degradation may generate structurally reactive, persistent, or bioaccumulative TPs whose environmental behavior diverges substantially from that of the parent compounds. Quinone-imines, halogenated anilines, demethylated aromatic dyes, and benzophenone-type carbonyls each pose distinct risks due to their redox activity, chemical stability, or strong affinity for soil organic matter. Moreira et al. (2025) directly underlines that TPs “cannot be pushed to the background”: they may affect photocatalytic performance, exhibit higher toxicity than parent compounds, and pose persistence/eco-toxicity risks [150]. Praus et al. demonstrates real cases where pharmaceuticals (e.g., ofloxacin, diclofenac) under visible-light photocatalysis produce intermediate TPs identified via high-resolution mass spectrometry—highlighting the necessity of TP profiling and not just parent-compound disappearance [172].

These characteristics promote prolonged environmental residence times, increase potential for sorption to rhizosphere substrates, and facilitate bioaccumulation in plant tissues. Ellepola et al. (2022) shows that degradation pathways (and thus TPs) depend on environmental context, implying that lab-scale results may not straightforwardly translate to environmental fate, relevant for soil/groundwater risk assessment [173].

Redox-active TPs, such as quinone-imines, are capable of continuous ROS cycling, producing sustained oxidative pressure on plant cells even at low exposure levels. Conversely, hydrophobic and planar aromatic intermediates, such as benzophenone-type carbonyls or halogenated anilines, exhibit limited biodegradability and strong interactions with humic substances, thereby shaping their vertical mobility and long-term retention in agricultural soils.

Such behavior complicates predictions of plant–soil exposure outcomes and challenges the assumption that parent-compound degradation inherently correlates with decreased toxicity. In many cases, TP mixtures present during intermediate stages of photocatalysis may exert higher phytotoxicity, genotoxicity, or microbial inhibition than the untreated pollutant. This is particularly relevant for agricultural reuse scenarios, where irrigation with treated effluents exposes plants, soil microorganisms, and root-associated ecosystems to evolving TP profiles over time. Consequently, evaluating the safety of photocatalytic processes cannot rely solely on bulk parameters such as color removal, COD reduction, or parent-compound disappearance. Kumar et al. (2025) exposes ecological risks related to textile dyes and their degradation by-products: soil toxicity, plant growth inhibition, altered nutrient/water uptake, oxidative stress, and bioaccumulation as an example bridging photocatalytic treatment and terrestrial ecosystem impacts [8].

To ensure environmentally meaningful assessments, photocatalytic treatment performance must be supported by TP profiling, molecular-level identification of persistent intermediates, and ecotoxicity bioassays reflecting plant–soil exposure routes. Complementary evaluation of TP fate, through sorption studies, biodegradation tests, and plant uptake measurements, provides essential evidence for determining whether treatment processes reduce overall ecological risk. Chen et al. (2022) reviewed anthraquinone-based photocatalysis and illustrated that, even when advanced catalysts are used, formation of persistent or reactive by-products is possible, emphasizing the need for careful mechanistic and by-product scrutiny before upscaling [17]. Kumari et al. (2023) provides a broader overview of limitations and potential drawbacks of photocatalytic wastewater treatments—including incomplete mineralization, formation of unknown TPs, and lack of standardization in toxicity/fate assessment [149]. Integrating these approaches into treatment design and monitoring frameworks enhances the reliability of photocatalysis for sustainable water management and minimizes unintended impacts on terrestrial ecosystems.

### 5.2. Identification and Characterization of Transformation Products

The identification and characterization of transformation products (TPs) generated during photocatalytic degradation are essential for understanding the environmental relevance of advanced oxidation processes. Although photocatalysis effectively reduces the concentration of pharmaceuticals and dyes, it rarely leads to complete mineralization; instead, partial oxidation often yields a cascade of intermediate compounds with variable persistence and toxicity. These transformation products may possess higher polarity, altered functional groups, or enhanced mobility in aqueous and soil matrices, thereby influencing their bioavailability to plants and soil microorganisms. Comprehensive TP profiling is thus a prerequisite for assessing the true ecotoxicological footprint of photocatalytic wastewater treatment systems.

#### 5.2.1. Analytical Strategies for TP Identification

State–of–the–art analytical approaches combine high-resolution chromatographic separation with mass spectrometric detection to elucidate TP structures and degradation pathways. Liquid chromatography coupled with high-resolution mass spectrometry (LC–HRMS or LC–QTOF–MS) has become the principal tool for non–target and suspect screening, enabling precise mass measurement and isotopic pattern analysis for structural elucidation [113,174]. Complementary spectroscopic methods such as Fourier-transform infrared spectroscopy (FTIR), nuclear magnetic resonance (NMR), and UV–Vis spectrophotometry assist in identifying functional group changes and structural modifications generated during photocatalytic degradation: functional group changes, including hydroxylation, dealkylation, deamination, or cleavage of chromophoric bonds. In dye degradation studies, for example, the disappearance of the –N=N– azo linkage and the emergence of aromatic amines have been confirmed through combined LC–MS and FTIR analysis [90,130].

#### 5.2.2. Transformation Pathways and Chemical Markers

Transformation products often follow distinct degradation routes depending on the parent compound structure and photocatalyst employed. For antibiotics such as sulfamethoxazole and tetracycline, hydroxylation and desulfonation represent the primary pathways under TiO_2_ or ZnO photocatalysis, yielding products such as sulfanilic acid and N^4^-acetylated derivatives [86,126]. For aromatic dyes such as malachite green and methylene blue, sequential N–demethylation, oxidative deamination, and aromatic ring cleavage dominate, producing leucomalachite green, benzophenones, and low–molecular–weight carboxylic acids [87,90,127,130]. The detection of such characteristic intermediates serves as chemical markers for monitoring degradation progress and evaluating catalyst selectivity.

#### 5.2.3. Relevance to Environmental Monitoring and Toxicity

Accurate TP identification not only delineates photocatalytic mechanisms but also supports risk assessment of treated effluents reused in agriculture. Several transformation products have been shown to retain antimicrobial or toxic properties, potentially affecting soil microbial balance or inducing oxidative stress in plants upon exposure. For instance, hydroxylated carbamazepine and benzoquinone–type derivatives exhibit residual phytotoxicity, influencing germination and chlorophyll synthesis [25,97,111,116,126,137]. Similarly, aromatic amines resulting from azo dye degradation are known to persist and accumulate in soil organic fractions, where they may undergo secondary transformations or leach into groundwater. These findings highlight the necessity of integrating analytical chemistry with ecotoxicological evaluation to establish the safety of photocatalytically treated wastewater for agricultural reuse.

Therefore, comprehensive TP identification, supported by advanced analytical platforms and mechanistic interpretation, forms a critical link between photocatalytic efficiency and environmental safety. Future research should focus on standardized methodologies for TP detection, improved databases for structure–toxicity prediction, and in situ monitoring protocols that bridge laboratory-scale findings with real-field applications.

### 5.3. Fate of TPs in Soils—Sorption, Mobility, and Biodegradability

Once transformation products (TPs) formed during photocatalytic treatment enter agricultural systems, their subsequent behavior in soils determines their long-term ecological impact and potential for plant uptake. Despite being derived from the partial oxidation of parent pharmaceuticals and dyes, many TPs persist in the environment and display distinct chemical and biological reactivities. Their mobility, bioavailability, and persistence are governed by multiple interrelated factors as: molecular structure, soil physicochemical parameters, redox potential, and microbial community composition, which collectively influence their partitioning, degradation, and transport [175,176]. Understanding these processes is fundamental for establishing safe reuse of photocatalytically treated wastewater and for predicting contaminant transfer within the soil–plant–water continuum.

#### 5.3.1. Sorption Processes and Binding Mechanisms

Sorption determines the extent to which TPs remain available in soil solution for microbial degradation or plant uptake. Depending on their polarity, ionization state, and functionalization, TPs can either strongly associate with soil colloids or remain mobile in pore water. Hydroxylated or carboxylated derivatives such as 10,11–epoxycarbamazepine, sulfanilic acid, or oxalic acid display lower sorption coefficients (Kd < 5 L kg^−1^) than their hydrophobic parent molecules (Kd 20–100 L kg^−1^) due to increased polarity and reduced hydrophobic interactions [4,61,105].

Electrostatic interactions also play a major role: under neutral to alkaline conditions, anionic TPs are repelled by negatively charged soil surfaces, whereas cationic intermediates such as N-demethylated dyes or fluoroquinolone–derived amines experience enhanced retention through cation exchange and surface complexation with clay minerals or humic colloids [8]. Moreover, π–π and hydrogen-bond interactions between aromatic TPs and humic macromolecules contribute to their selective immobilization in organic-rich soils.

The strength and reversibility of sorption control the balance between short–term bioavailability and long–term accumulation. While strong sorption may limit acute plant exposure, it can result in progressive buildup in the rhizosphere, where desorption may occur under changing redox or pH conditions.

#### 5.3.2. Mobility and Leaching Dynamics

The mobility of TPs in soils depends on the interplay between their aqueous solubility, soil texture, and hydrodynamic conditions. Hydrophilic TPs, such as short-chain organic acids, hydroxylated antibiotics, and N-demethylated dye fragments, are typically characterized by high mobility and low affinity for solid phases. Field and column studies show that such compounds can migrate through soil profiles and reach groundwater, particularly under intensive irrigation regimes using reclaimed water [48,85,88,125].

In sandy soils with low organic carbon and CEC, vertical transport of TPs is enhanced due to weak sorption and high hydraulic conductivity, while in fine-textured or clayey soils, diffusion and micropore entrapment slow down percolation. The presence of dissolved organic matter (DOM) in treated effluents can significantly increase TP mobility by forming soluble organo–pollutant complexes or colloidal carriers, particularly for aromatic and polar intermediates [93,133].

Additionally, environmental factors such as rainfall events, irrigation frequency, and temperature fluctuations influence leaching fluxes. Under high moisture conditions, desorbed TPs may move downward and interact with subsoil microbial communities, altering their degradation potential. Conversely, drying–wetting cycles can enhance sorption hysteresis and promote aging of bound residues.

#### 5.3.3. Biodegradability and Microbial Transformation

Biodegradability is one of the key determinants of TP persistence. Photocatalytic oxidation frequently introduces oxygenated groups (–OH, –COOH, –CHO), increasing hydrophilicity and susceptibility to enzymatic degradation. However, not all intermediates are biodegradable; some acquire structural stability that resists microbial attack. For example, hydroxylated carbamazepine, benzoquinone derivatives, and aromatic amines formed during TiO_2_ or ZnO photocatalysis show limited mineralization in soil microcosms, with half-lives exceeding 60 days [85,90,125,130]. Conversely, low-molecular-weight products such as oxalic, maleic, or formic acids are rapidly metabolized by heterotrophic bacteria within hours to days.

Microbial transformation routes often involve oxidative or reductive enzymatic steps mediated by soil bacteria and fungi, including peroxidases, laccases, and oxygenases. The diversity and abundance of microbial taxa, particularly *Pseudomonas*, *Bacillus*, and *Trichoderma* spp., strongly influence TP biodegradation rates [93,133]. Environmental parameters such as pH, moisture, and temperature further regulate enzymatic activity and oxygen diffusion, creating spatial heterogeneity in TP turnover.

In addition, secondary transformation of TPs can yield both detoxified and more toxic species. For instance, aromatic amines resulting from azo dye degradation may undergo oxidative coupling to form persistent azoxy or polyaromatic compounds, while hydroxylated pharmaceuticals may form reactive quinones capable of binding to soil macromolecules, creating bound [33,39]. This duality underscores the importance of integrating biodegradation studies with toxicity assessments.

The complex behavior of transformation products (TPs) derived from photocatalytic degradation of pharmaceuticals and dyes in soils is summarized in Table 8, which compiles data from recent literature concerning their sorption characteristics, mobility, and biodegradability. The Table highlights how physicochemical factors, such as soil texture, organic matter, and pH, govern the retention and transport of TPs including 10,11–epoxycarbamazepine, N^4^–acetyl–sulfamethoxazole, and aromatic amines. Comparisons among studies show that hydroxylated or carboxylated derivatives tend to exhibit lower sorption coefficients and higher mobility than their parent compounds, while structural complexity and aromaticity increase persistence. The integration of batch, column, and microbial degradation data offers insight into the coupled processes that control TP fate, providing a foundation for evaluating their long-term environmental implications in agricultural systems irrigated with photocatalytically treated wastewater.

#### 5.3.4. Persistence, Aging, and Soil Compartmentalization

Over time, TPs can transition from labile to bound fractions, becoming part of the soil organic matrix through covalent linkage or co-precipitation with minerals. This “aging” process reduces extractability and immediate bioavailability but does not necessarily imply complete detoxification. Studies show that aged residues can be remobilized under changing environmental conditions, such as pH reduction, root exudation, or flooding, potentially re-exposing soil biota and crops [61,101].

Persistent TPs accumulate predominantly in the topsoil (0–20 cm), where microbial activity and organic matter are highest, but some polar intermediates can leach to subsoil layers. Their persistence is often correlated with low mineralization rates (k < 0.01 day^−1^) and high partition coefficients to organic carbon (K_OC_ > 200 L kg^−1^). The combined processes of sorption, entrapment, and slow microbial transformation contribute to the long-term environmental footprint of photocatalytically treated wastewater residues [4].

#### 5.3.5. Environmental and Agronomic Implications

The persistence and movement of TPs in soil–plant systems carry potential ecological risks. Weakly sorbing and mobile intermediates can enter the root zone, accumulate in plant tissues, and induce oxidative stress or genotoxic responses. Conversely, strongly bound TPs may alter microbial dynamics, enzyme activity, and nutrient cycling within the rhizosphere [16].

Long-term exposure scenarios raise concerns about cumulative toxicity, especially under continuous irrigation with photocatalytically treated effluents. Therefore, assessing TP fate requires a multi-parameter approach that combines physicochemical modeling (e.g., sorption isotherms, leaching indices), biodegradation kinetics, and bioassays. Future research must emphasize the development of predictive frameworks linking soil properties, TP structure, and biological effects to support risk-based management of treated wastewater reuse in agriculture [12].

**Figure 14:**
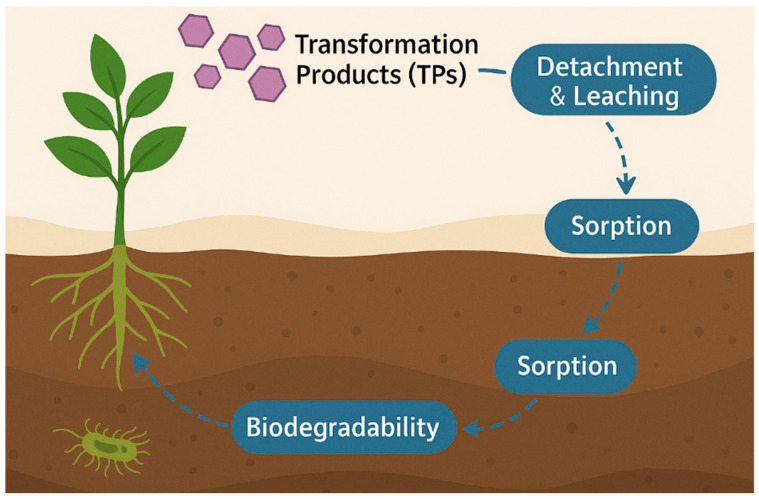
Fate of photocatalytic transformation products (TPs) in soil–plant systems.

The processes governing the fate of photocatalytic transformation products (TPs) in soil–plant systems are complex and highly dynamic, as illustrated in Figure 13 and Figure 14, which summarize the key interactions controlling their environmental behavior. This Figure describes the simultaneous occurrence of adsorption and desorption processes on soil particles, vertical leaching of hydrophilic intermediates through soil pores, and microbial degradation within the rhizosphere and subsoil. The representation also highlights the potential for root uptake and translocation of polar TPs such as hydroxylated carbamazepine, sulfanilic acid, and aromatic amines, illustrating how soil properties, microbial activity, and water movement jointly determine their persistence and mobility.

### 5.4. Comparative Toxicity to Plants and Soil Organisms—Parent vs. Degraded Compounds

Photocatalytic degradation processes are often regarded as environmentally benign because they effectively transform persistent pharmaceutical and dye pollutants into smaller, more oxidized intermediates. However, the toxicity of transformation products (TPs) relative to their parent compounds is increasingly recognized as a critical factor in assessing the sustainability of advanced oxidation treatments [33,39,120,183]. While parent compounds such as carbamazepine, sulfamethoxazole, diclofenac, and methylene blue are well-documented for their ecotoxicological effects on soil–plant systems, several of their photocatalytic intermediates can retain biological activity or even exhibit enhanced reactivity, leading to comparable or greater stress responses in plants and soil organisms [86,90,126,130].

Table 9 highlights that photocatalytic degradation reduces persistence but not necessarily ecotoxicity. While parent pollutants such as carbamazepine and methylene blue are stable and hydrophobic, their transformation products (TPs) become more polar and reactive, leading to stronger oxidative and genotoxic effects on soil microorganisms and plants [17,64,104,121,122,123,124,125,126,184,185,186,187,188]. These differences underline the necessity of assessing both chemical degradation efficiency and post-treatment biological impact when evaluating advanced oxidation processes for wastewater treatment and agricultural reuse.

#### 5.4.1. Phytotoxic Responses to Transformation Products

Comparative bioassays reveal that TPs can induce distinct and sometimes stronger phytotoxic effects than the parent pollutants. For example, hydroxylated and epoxy derivatives of carbamazepine generated during TiO_2_ and ZnO photocatalysis were shown to suppress seed germination, reduce chlorophyll content, and increase lipid peroxidation in *Lactuca sativa* and *Triticum aestivum* seedlings compared to the parent compound at equivalent concentrations [85,125,127,189]. Similarly, N4-acetyl-sulfamethoxazole (Ac-SMX), formed during oxidative treatment of SMX, has been reported to exhibit longer persistence and comparable inhibitory effects on root elongation and biomass accumulation in *Sorghum bicolor* [83,123].

In many cases, enhanced phytotoxicity results from increased polarity and bioavailability of TPs, which facilitates root uptake and translocation through the xylem. However, structural modifications such as aromatic ring hydroxylation or amide oxidation can also enhance electron transfer capacity and promote the generation of reactive oxygen species (ROS) within plant tissues [23,26,128,190]. The induction of oxidative stress markers, particularly superoxide dismutase (SOD), catalase (CAT), and malondialdehyde (MDA) has been consistently reported in plants exposed to post-photocatalytic residues of pharmaceuticals and dyes, indicating that partial degradation does not necessarily equate to detoxification.

#### 5.4.2. Soil Microbial and Faunal Sensitivity

Soil microbial communities, which play vital roles in nutrient cycling and organic matter decomposition, are particularly sensitive to residual TPs. Studies using TiO_2_- and ZnO-treated effluents show that while parent pharmaceuticals may inhibit total microbial biomass and enzyme activities (e.g., dehydrogenase, urease, phosphatase), their degradation products can disrupt microbial community composition, favoring resistant taxa such as *Pseudomonas*, *Acinetobacter*, or *Bacillus* spp. [85,125,129,130,175,191].

For instance, aromatic amines and hydroxylated quinones derived from dye degradation interfere with microbial respiration and denitrification processes, reducing soil metabolic diversity [131,192].

Additionally, earthworms (*Eisenia fetida*) and nematodes have demonstrated differential sensitivity to parent and degraded pollutants. In chronic exposure tests, earthworms exposed to photocatalytically treated methylene blue residues exhibited higher levels of DNA strand breaks and lipid peroxidation than those exposed to the parent dye, likely due to reactive intermediates such as leucomalachite green and benzaldehyde-type compounds [33,39,90,130]. These findings highlight that partial oxidation may convert stable molecules into transient electrophilic species capable of covalent interactions with proteins and nucleic acids, amplifying sublethal toxicity.

#### 5.4.3. Mechanistic Insights into Altered Toxicity

The shift in toxicity profiles from parent to degraded compounds is strongly influenced by molecular transformation pathways. Oxidative hydroxylation, nitration, or demethylation increases the number of functional groups that participate in hydrogen bonding and redox reactions, often enhancing the ability of intermediates to generate intracellular ROS or bind to macromolecules. Some TPs also form quinoid structures, which act as redox-cycling agents capable of producing superoxide anions (O_2_•^−^) and hydrogen peroxide (H_2_O_2_) under physiological conditions [33,39].

Conversely, certain TPs undergo complete mineralization to low-molecular-weight organic acids (e.g., oxalic, formic, or acetic acids), which are rapidly metabolized by soil microorganisms and have negligible toxicity [85,125]. Therefore, toxicity outcomes depend on both the structural identity and environmental persistence of each intermediate, emphasizing the need for integrated analyses that combine chemical identification with biological endpoints.

#### 5.4.4. Comparative Assessment and Implications for Risk Management

Overall, a synthesis of available data suggests that 30–60% of photocatalytic transformation products retain measurable toxicity in plant or soil bioassays [90,129,130,175]. The environmental hazard of photocatalytically treated wastewater thus depends not only on the degree of parent compound removal but also on the formation and persistence of these by-products. Comparative toxicity evaluations using multiple biological endpoints: germination, chlorophyll fluorescence (Fv/Fm), enzymatic assays, and microbial respiration are therefore crucial for comprehensive risk assessment.

Furthermore, environmental conditions such as pH, organic matter content, and light exposure modulate the fate and impact of TPs in soil ecosystems. In alkaline or low-organic soils, mobile, polar intermediates can enter the rhizosphere and accumulate in roots, while in organic-rich or clayey soils, stronger sorption reduces immediate toxicity but enhances long-term accumulation [54,94]. Thus, the interplay between soil properties, TP structure, and exposure duration determines the overall ecotoxicological profile of post-photocatalytic residues.

## 6. Comparative Ecotoxicological Assessment Pre– and Post–Photocatalysis

### 6.1. Synthesis of Experimental Findings—Summary of Studies Comparing Untreated and Treated Effluents

#### 6.1.1. Experimental Evidence of Detoxification and Transient Toxicity During Photocatalytic Treatment

Across plant bioassays, a consistent pattern emerges: photocatalysis generally reduces phytotoxicity compared with untreated effluents, provided that mineralization is extensive. Partial oxidation, however, can transiently increase toxicity. Early work on real textile effluents treated by TiO_2_ and TiO_2_/H_2_O_2_ showed high discoloration and substantial TOC removal (≈66–80% after 6 h), and, critically, lower phytotoxicity to *Lactuca sativa* seeds than the raw effluent. Yet, during the early irradiation period toxicity could rise (more markedly with TiO_2_/H_2_O_2_), before falling as treatment progressed [132,133,193,194].

For pharmaceutical mixtures, plant assays indicate that detoxification often tracks treatment time/mineralization. In a study summarized by Krakowiak et al. (2021), untreated mixtures of atenolol, chlorpromazine and metronidazole caused rapid mortality and growth inhibition in *Spirodela polyrrhiza*; after 8 h of TiO_2_ photocatalysis, plants still showed reduced growth (reflecting harmful intermediates), whereas 16 h of treatment eliminated growth inhibition (near-baseline RGR/RFN), evidencing effective detoxification post-mineralization [134,195]. At the same time, critical reviews of real-effluent applications warn that photocatalysis can generate more toxic transformation products (TPs) if stopped prematurely or operated sub-optimally. Compilations of municipal/industrial case studies conclude that toxicity removal is not guaranteed by parent-compound decay, recommending toxicity tracking alongside chemical metrics and favoring longer residence times or coupled processes to suppress TP-driven effects [55,135].

Evidence from field-relevant matrices supports this nuance. In stream waters treated by TiO_2_ under optimized but incomplete conditions, intermediate samples remained significantly phytotoxic to *Lactuca sativa*, whereas fully mineralized samples did not, underscoring the importance of treatment completeness and matrix effects [136,196]. Likewise, coupled biological + solar photocatalysis applied to mixed pharmaceuticals reports progressive toxicity attenuation during treatment (evaluated with standard bioassays), but again emphasizes that intermediate time points can be misleading if used as end-points [137,197].

Mechanistically, recent work with sulfonamides shows why detoxification may lag concentration decay: TPs formed during photocatalysis can retain or even exceed baseline ecotoxicity, requiring either extended light exposure to drive mineralization or post-photocatalytic polishing (e.g., aerobic biodegradation) to transform persistent TPs into less bioactive species [56,138]. Together, these findings align with the textile and pharmaceutical case studies: when photocatalysis proceeds to high mineralization, plant growth and germination recover; when halted early, plant endpoints can signal residual or enhanced toxicity [55,132,134,135,193,195].

#### 6.1.2. Endpoints and Sensitivity

Plant endpoints commonly used include seed germination/root elongation (*Lactuca sativa*), frond number and relative growth rate (*Spirodela*/*Spirodela*–*Lemna*), and occasionally cyto/genotoxicity assays with *Allium cepa*. Across studies, growth-based duckweed assays (72 h) and lettuce root growth prove sensitive to transient toxicity during partial oxidation, while germination percentages alone can miss sub-lethal effects. Hence, multi-endpoint panels (growth + photosynthetic performance or genotoxicity) better resolve detoxification trajectories pre-/post-treatment [55,132,134,135,193,195].

#### 6.1.3. Implications for Soil–Plant Systems

Direct soil studies remain fewer than aqueous plant bioassays, but the message is consistent: effluents destined for irrigation demand toxicity-guided endpoints, not only chemical removal, and hybrid trains (bio + photocatalysis; extended irradiation; or post-biological polishing) stabilize detoxification and limit TP carryover into the root zone [55,134,135,138,195,198].

#### 6.1.4. Bottom Line

Compared with untreated effluents, photocatalytically treated waters tend to be less phytotoxic to higher plants when mineralization is sufficient; partial treatment can transiently elevate toxicity; so, treatment completeness and toxicity–based monitoring are essential to ensure safe reuse in agro–soil–plant systems [55,56,132,134,135,138,193,195].

Table 10 summarizes representative studies that compared the phytotoxicity of untreated and photocatalytically treated effluents, highlighting differences in experimental matrices, catalysts, and plant bioassays. It complements the narrative synthesis by emphasizing mechanistic details such as the role of intermediate transformation products, correlations between organic carbon removal and toxicity attenuation, and the influence of hybrid treatment configurations [148,198,199,200,201,202,203,204,205].

Collectively, these results illustrate how treatment completeness, catalyst type, and effluent composition determine the extent of detoxification, confirming that partial oxidation can transiently enhance toxicity while full mineralization or coupled post-biological steps achieve effective ecotoxicological reduction.

### 6.2. Quantitative Analysis—Magnitude and Direction of Toxicity Change (Reduction, Persistence, Enhancement)

Quantitative evaluation of phytotoxicity variation before and after photocatalytic treatment provides a clearer understanding of the extent and consistency of detoxification across pollutant classes, catalysts, and bioassays. The magnitude of toxicity change can be expressed as the ratio between biological response metrics (e.g., germination rate, relative root length, frond number, or growth rate) for treated versus untreated effluents, allowing for the direction of change, as reduction, persistence, or enhancement, to be objectively established.

Across available studies, most photocatalytic treatments yield toxicity reductions between 50 and 100%, depending on the degree of mineralization and reactor configuration [148,206]. In the textile effluent study by Garcia et al. (2009), *Lactuca sativa* root elongation increased by approximately 70% compared with the untreated control after 6 h of TiO_2_ irradiation, correlating with TOC reduction exceeding 65% [132,193]. Conversely, samples withdrawn after only 2 h exhibited a transient 25% inhibition increase, attributed to short-lived peroxidic intermediates. For pharmaceutical mixtures, Krakowiak et al. (2021) reported a complete reversal of growth inhibition in *Spirodela polyrrhiza* after 16 h of photocatalysis, equivalent to a 100% detoxification efficiency relative to untreated mixtures that initially suppressed growth by 90% [134,195].

When comparing real effluents, Rueda-Márquez et al. (2020) found that the average reduction in toxicity among multiple case studies ranged between 40 and 85%, with marked variability linked to water matrix complexity and transformation product (TP) persistence [55,135]. Similar patterns were observed in solar–driven systems, where Stella et al. (2022) quantified a twofold improvement in lettuce root growth after complete mineralization [136,196], while intermediate samples remained inhibitory, confirming that detoxification follows a non-linear trend with treatment time. In hybrid bio–photocatalytic systems, Aliste et al. (2024) observed a gradual decrease in overall toxicity of 60–90% across sequential stages, emphasizing the kinetic synergy between biological pre-treatment and photodegradation in minimizing TP accumulation [137,197].

Nevertheless, several datasets reveal cases of toxicity persistence or even enhancement during incomplete photocatalysis. For sulfonamides, Madej–Knysak et al. (2024) showed that partial oxidation generated nitro- and sulfone-intermediates with 20–40% higher residual ecotoxicity than parent compounds, only eliminated after coupling with aerobic biodegradation [56,138]. These findings indicate that the direction of toxicity change depends not solely on pollutant removal efficiency but also on TP reactivity, redox properties, and stability in water or soil environments.

Overall, quantitative analyses confirm that photocatalytic detoxification is effective but not linear: toxicity typically decreases sharply after 60–80% mineralization, with diminishing returns or persistence observed below that threshold. Complete detoxification corresponds to near-total mineralization (TOC < 10%) or integration with a secondary biological polishing step. Thus, numerical indicators of toxicity reduction, expressed as percent inhibition decrease or endpoint recovery are essential to distinguish between true detoxification and apparent pollutant removal, ensuring a robust ecotoxicological assessment of photocatalytically treated effluents prior to agricultural reuse.

To better illustrate the variability in phytotoxicity outcomes across different photocatalytic systems and effluent types, Table 11 presents a quantitative synthesis of reported studies comparing biological responses before and after treatment.

Table 11 highlights the magnitude and direction of toxicity change, ranging from transient enhancement during partial oxidation to substantial or complete detoxification upon advanced mineralization. By linking toxicity variation with parameters such as total organic carbon (TOC) or dissolved organic carbon (DOC) removal, it provides a clearer picture of how treatment efficiency, pollutant matrix, and process configuration jointly influence ecotoxicological outcomes. These data reinforce that true detoxification correlates more closely with mineralization completeness and transformation product degradation than with pollutant disappearance alone.

### 6.3. Factors Influencing Detoxification Outcomes—Catalyst Type, Water Matrix, Soil Conditions

The efficiency of photocatalytic detoxification and the subsequent reduction in phytotoxicity depend strongly on catalyst characteristics, composition of the treated matrix, and post-treatment soil conditions. These interconnected factors govern the generation of reactive oxygen species (ROS), transformation product (TP) profiles, and pollutant bioavailability in plant–soil systems.

Catalyst type and modification play a crucial role in determining detoxification outcomes. Traditional photocatalysts such as TiO_2_ and ZnO are well established for degrading pharmaceuticals and dyes, yet their efficiency under real conditions varies with crystallinity, surface area, and bandgap energy. Studies indicate that anatase-phase TiO_2_ typically achieves the highest mineralization rates, correlating with substantial reductions in phytotoxicity [134,195]. Doping with non-metals (e.g., N, C, S) or metals (e.g., Fe, Cu, Ag) can enhance visible-light absorption and suppress recombination of photogenerated charge carriers, but may also alter degradation pathways, sometimes producing persistent intermediates that sustain residual toxicity [55,135]. Similarly, heterojunction composites such as TiO_2_/g-C_3_N_4_ or ZnO/CuO have shown higher degradation efficiencies and faster disappearance of reactive aromatic intermediates, leading to improved detoxification compared with pristine catalysts [55,56,135,138].

The composition of the water matrix substantially influences the apparent detoxification performance. Natural organic matter, bicarbonates, and suspended solids can act as ROS scavengers, reducing the oxidation rate and thereby delaying pollutant mineralization [136,196]. Conversely, the presence of nitrate or chloride ions can modify reaction kinetics and promote the formation of halogenated or nitro-aromatic by-products, sometimes more toxic than the parent compounds [132,193]. Photocatalytic studies on real industrial and municipal effluents reveal that high chemical complexity often results in lower detoxification efficiency (40–85%) compared with single-compound systems, primarily because of matrix interferences and limited light penetration [55,135]. Thus, interpreting toxicity trends in real matrices requires simultaneous monitoring of degradation intermediates and assessing potential synergistic effects among pollutants.

Following treatment, soil conditions determine the persistence, mobility, and eventual impact of residual pollutants and their transformation products. Soil pH, redox potential, and organic matter content modulate adsorption–desorption equilibria and influence plant uptake potential. For instance, alkaline soils may accelerate degradation of acidic intermediates, enhancing detoxification, whereas high organic content can increase pollutant retention, prolonging exposure to plant roots and rhizosphere microorganisms [137,197]. Experiments involving irrigation with photocatalytically treated wastewater indicate that soils with higher microbial activity can further attenuate low-level residues, achieving combined chemical and biological detoxification. Conversely, soils with low buffering capacity or high salinity can amplify stress responses even when waterborne pollutant concentrations are reduced [56,138].

**Figure 15:**
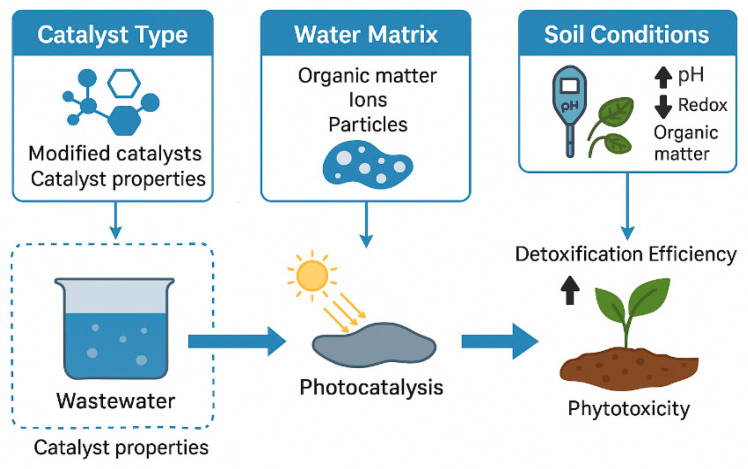
Process schematic illustrating the influence of catalyst type, water matrix composition, and soil conditions on detoxification outcomes after photocatalytic treatment. The diagram shows the transition from pollutant-containing wastewater through photocatalytic degradation to treated effluents interacting with soil–plant systems, highlighting pathways leading to toxicity reduction, persistence, or enhancement depending on process and environmental factors.

Figure 15 illustrates the overall process and key factors governing detoxification outcomes following photocatalytic treatment of contaminated effluents. The schematic integrates the main stages, from pollutant-laden wastewater undergoing photocatalysis under UV or solar irradiation to the subsequent interactions of treated effluents with soil–plant systems. It highlights how catalyst characteristics (composition, morphology, and modifications), water matrix complexity (presence of ions, natural organic matter, and turbidity), and soil conditions (pH, organic matter, and microbial activity) collectively shape the magnitude and direction of toxicity changes. Arrows representing pathways of toxicity reduction, persistence, or enhancement emphasize the need to consider both treatment efficiency and post-application environmental dynamics when evaluating photocatalytic detoxification performance.

Overall, detoxification outcomes reflect the synergistic effects of catalyst functionality, matrix composition, and soil reactivity. Optimizing these parameters, by selecting efficient visible-light catalysts, minimizing matrix interference, and ensuring favorable soil redox and pH conditions, enhances the sustainability of photocatalytically treated effluents for reuse in agricultural or ecological applications.

### 6.4. Relevance for Agricultural Reuse—Safe Limits for Irrigation and Soil Application

Safe reuse of treated effluents in agriculture relies on two complementary pillars: (i) compliance with reclaimed-water quality criteria that primarily address microbial risks, and (ii) agronomic/soil-protection thresholds that prevent salinity, sodicity, or specific-ion toxicity. In the EU framework, reclaimed water for crop irrigation is categorized into Classes A–D, with *E. coli* limits of ≤10, ≤100, ≤1000 and ≤10,000 cfu 100 mL^−1^, respectively; Class A also includes BOD_5_ ≤ 10 mg L^−1^, TSS ≤ 10 mg L^−1^, turbidity ≤ 5 NTU, and preventive criteria for *Legionella* spp. (≤1000 cfu L^−1^ when aerosolization risk) and helminth eggs (≤1 egg L^−1^ for fodder/pastures) [147,149,207,208]. WHO’s risk-based guidance converges on health-based targets using *E. coli* and helminth reduction, operationalized through multiple barriers and validation monitoring [150,209]. In the U.S., the EPA reuse guidance aligns reclaimed-water classes to end-use risk, combining microbial targets with performance– and monitoring–based controls [151,210]. ISO 16075 provides design–to–operation guidance for irrigation projects using treated wastewater, embedding risk management, fit-for-purpose treatment, and irrigation-method controls [152,211].

For agricultural users, several additional parameters are essential for evaluating the suitability of photocatalytically treated wastewater for irrigation. These include: (i) residual concentrations of pharmaceuticals, dyes, and their major transformation products; (ii) total organic carbon (TOC) and specific UV absorbance (SUVA) as indicators of remaining reactive intermediates; (iii) phytotoxicity endpoints such as germination inhibition or root elongation reduction; (iv) residual oxidants or catalyst particles originating from the treatment process; and (v) soil–water quality parameters such as electrical conductivity, sodium adsorption ratio, and nutrient balance. For crops irrigated through sprinkler systems, a short pre-harvest interval of 3–7 days limits the presence of surface residues, especially in leafy vegetables.

**Figure 16:**
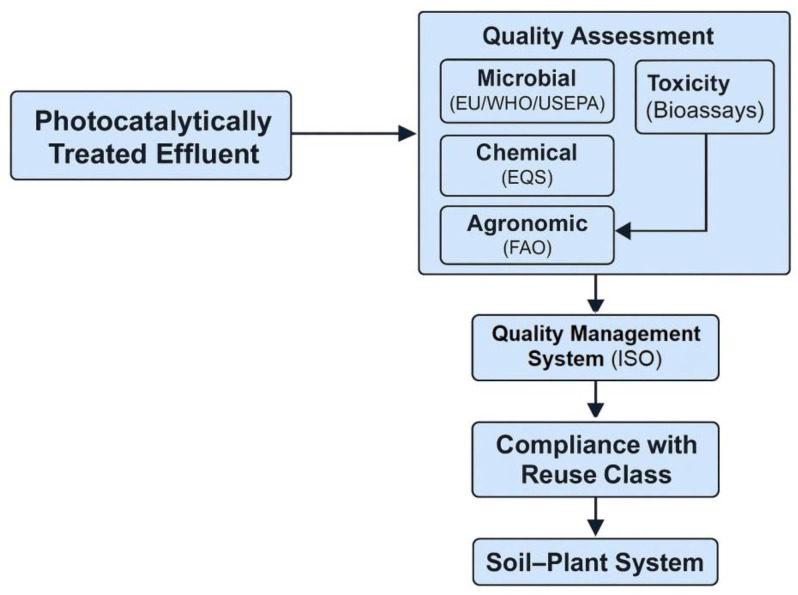
Conceptual framework illustrating the pathway from photocatalytic treatment of wastewater to its safe agricultural reuse (EQS—Environmental Quality Standards; EU—European Union; WHO—World Health Organization; USEPA—United States Environmental Protection Agency; ISO—International Organization for Standardization; FAO—Food and Agriculture Organization of the United Nations).

To complement the regulatory and agronomic overview, Figure 16 provides a visual synthesis of the integrated framework governing the safe agricultural reuse of photocatalytically treated effluents. It illustrates how treatment processes, quality assessment modules, and international guidelines interact to ensure environmental and crop safety. The diagram links the photocatalysis stage with subsequent microbial, chemical, agronomic, and toxicity evaluations, showing how these are aligned under global frameworks such as EU Regulation 2020/741, WHO and USEPA guidelines, ISO 16075, and FAO water quality standards [149,150,151,152,208,209,210,211]. This integrative perspective highlights the need for multi-criteria validation, combining conventional water-quality indicators with bioassays and soil compatibility assessments before treated effluents can be safely reused for irrigation or soil application.

From an agronomic perspective, safe application depends on classical irrigation-water quality criteria that protect soil function and crop performance. FAO guidelines set indicative thresholds for electrical conductivity (salinity hazard), sodium adsorption ratio (infiltration hazard), and specific ions such as Cl^−^, Na^+^ and B, which directly affect plant growth and soil structure [153,212]. These criteria remain essential for reclaimed water because advanced oxidation or photocatalysis does not alter salinity or major-ion composition. Where soil application of biosolids is relevant, the EU Sewage Sludge Directive 86/278/EEC constrains heavy metals in sludge and in receiving soils to prevent long-term accumulation; however, it sets no numeric limits for pharmaceuticals or dyes [154,155,213,214]. For chemical pollutants in surface waters, Environmental Quality Standards (EQS) under the Water Framework Directive regulate priority substances, but few pharmaceuticals/dyes are covered, and EQS do not directly translate to irrigation–water reuse [154,213]. For photocatalytically treated effluents, the regulatory picture is clear on microbes and conventional parameters, while trace organics (parent compounds and transformation products, TPs) lack harmonized numeric limits in irrigation reuse. European Commission (2020) and Joint Research Center (2017) position these under project–level risk management, with monitoring plans tailored to local crops, irrigation methods, and exposure pathways [147,149,207,208]. In practice, fitness for agricultural reuse rests on demonstrating: (1) compliance with the reclaimed-water class suitable for the crop category and irrigation method [147,203], and (2) agronomic compatibility per FAO thresholds for salinity, sodicity, and specific ions [153,212]. Because photocatalysis can temporarily increase phytotoxicity if treatment is incomplete, effect–based verification (plant bioassays alongside chemistry) provides an extra margin of safety before irrigation or soil application [147,150,151,207,209,210].

Key takeaways for agricultural reuse: microbial safety targets (*E. coli*, helminths, *Legionella*) and conventional quality metrics are established at EU/WHO/EPA level; salinity and ion hazards are addressed by FAO agronomic thresholds; micropollutants and TPs are managed via risk assessments and monitoring plans linked to use class, crop, and site conditions rather than fixed numeric limits [147,149,150,151,153,207,208,209,210,212].

Table 12 marks the principal international frameworks and regulatory guidelines governing the safe reuse of treated wastewater in agriculture. It consolidates the key microbiological, chemical, and agronomic criteria defined by major organizations, including the European Union, WHO, USEPA, ISO, and FAO, as well as soil–protection limits under the EU Sewage Sludge Directive. By presenting these standards side by side, the Table highlights both the convergence of global approaches, focusing on microbial safety and soil protection and their complementary scope, ranging from risk-based public health targets to agronomic and environmental quality thresholds essential for long–term soil–plant sustainability.

For agricultural users, additional parameters relevant for crop and soil safety include: (i) residual concentrations of pharmaceuticals, dyes, and major transformation products; (ii) total organic carbon (TOC) and specific UV absorbance (SUVA) as indicators of reactive intermediates; (iii) phytotoxicity bioassays such as germination or root-elongation tests; (iv) residual oxidants or catalyst particles; and (v) electrical conductivity, sodium adsorption ratio, and nutrient balance. For leafy vegetables irrigated with reclaimed water, a short pre-harvest interval of 3–7 days limits the presence of surface residues, particularly under sprinkler irrigation [18,24,28,119,147,182,207].

## 7. Implications for Plant–Soil Health and Environmental Risk Assessment

### 7.1. Pollutant Accumulation and Soil Functioning—Enzyme Activities, Nutrient Cycling, and Microbial Balance

Residual contaminants and transformation products (TPs) that reach soils via treated effluents can alter core soil functions even when aqueous phytotoxicity appears attenuated. Incompletely mineralized organics, including pharmaceutical residues and their TPs, partition to soil colloids and organic matter, changing bioavailability and exposure windows for microbes and enzymes that drive C–N–P cycling [56,138,156,215]. Field and mesocosm evidence indicates that reclaimed/treated wastewater irrigation (TWW) reshapes soil microbiomes and functional potential, with the direction and magnitude of effects governed by water chemistry (salinity, nutrients, trace organics) and soil context (texture, pH, SOC) [157,158,216,217].

- Soil enzyme activities

Dehydrogenase, urease, and phosphatases are widely used integrators of microbial oxidative and hydrolytic capacity. Short–term TWW inputs can stimulate enzyme activities via nutrient and labile–C subsidies, while elevated salinity, metals, or reactive TPs may suppress activities, as patterns that meta–analyses and controlled studies now quantify [21,23,157,216]. For example, in reclaimed–water trials, increased hydrolytic activity co–occurred with higher mineral N availability, yet sites with long TWW histories showed shifts toward stress-tolerant taxa and reduced redox enzyme signals when salinity and metal loads accumulated. These outcomes underscore the need to interpret enzyme datasets alongside drivers such as EC/SAR and TP profiles, following harmonized SOPs for sampling and assay conditions [21,23,157,159,216,218].

- Nutrient cycling

TWW commonly elevates inorganic N and P in soils, accelerating short-term mineralization and plant uptake; however, concomitant changes in microbial community structure can modulate nitrification/denitrification and organophosphorus turnover, with feedbacks on N-use efficiency and P availability [157,158,216,217]. Where residual pharmaceuticals persist, selective inhibition of nitrifiers and phosphatases has been reported, consistent with enzyme–level responses and community shifts [138,156,198,215].

Analytical advances now enable multi-residual tracking of >40 pharmaceutical classes across soil–plant–biota compartments, supporting tighter linkage of mass balances to process indicators [160,161,219,220].

- Microbial balance and community structure

High-resolution sequencing shows irrigation-water quality as a primary architect of soil microbiomes across arid and semi–arid systems: saline and wastewater sources select for halotolerant or pollutant-resistant clades (e.g., *Actinobacteria*, *Firmicutes*), while reducing sensitive taxa and altering evenness, with measurable consequences for enzyme profiles [158,217].

Long-term TWW sites exhibit increased heavy–metal resistance determinants and distinct community assemblies, indicating sustained selection pressures that can decouple enzyme potential from short-term nutrient subsidies [21,23].

- Role of transformation products

Photocatalysis reduces parent loads but may transiently generate bioactive TPs (e.g., sulfonamide sulfones/nitro–derivatives) that retain or exceed baseline bioactivity until further mineralized or biopolished; such intermediates are detectable in soils receiving residuals and can modulate microbial function [56,138,156,215]. Hence, functional verification should accompany chemical endpoints when validating treated effluents for land application.

- Assessment implications

Robust risk evaluation benefits from a triad of (i) standardized enzyme assays (dehydrogenase, urease, phosphatases, catalase), (ii) microbial community profiling and resistance markers, and (iii) nutrient balance indicators (mineral N, Olsen-P) interpreted with EC/SAR and multi-residual analytics [157,159,161,216,218,220].

Together, these lines of evidence distinguish benign nutrient subsidies from stressor-driven functional impairment, guiding management of treated (including photocatalytically treated) effluent reuse in soil–plant systems [21,23,158,217].

### 7.2. Risk Assessment Framework—PEC/PNEC Ratios, Hazard Quotients for Parent and Transformation Products

Environmental risk for contaminants introduced via treated or reclaimed waters is commonly characterized using the ratio of predicted (or measured) exposure to effect thresholds: the hazard quotient HQ = PEC/PNEC, where PEC is the predicted environmental concentration (or a representative measured concentration, MEC) and PNEC is the predicted no-effect concentration derived from ecotoxicity data with appropriate assessment factors [162,163,221,222].

For pharmaceuticals and other micropollutants, PNECs are typically obtained from chronic no-observed-effect concentrations (NOECs) or effect concentrations (EC10/EC20) for algae, crustaceans and fish, applying assessment factors in a tiered manner and adopting the most sensitive trophic–level value for risk characterization [162,164,221,223]. Where chronic data are lacking, acute–to–chronic extrapolation or read–across may be used with higher assessment factors, while mixture risk is addressed by summing substance–specific HQs to obtain a mixture risk quotient, ΣHQ [165,224].

For reclaimed water and irrigation scenarios, exposure estimation should reflect local use patterns. Surface-water PECs downstream of discharges can be estimated using effluent concentrations and realistic dilution, while soil PECs under irrigation can be calculated from application rates, irrigation frequency, degradation, and sorption (partitioning) in the soil compartment; spatially referenced exposure models now integrate per-capita wastewater flows and plant-specific loads to refine PECs at catchment scales [6,161,166,225,226]. When monitoring data are available, MECs may replace model PECs, but must represent relevant exposure windows for biota [163,222].

Transformation products (TPs) formed during treatment (including photocatalysis) can materially affect risk outcomes. The revised EMA guideline explicitly considers total residue concepts and TPs in the environmental risk assessment for medicinal products, recommending targeted analysis where TPs are persistent or bioactive [162,221]. Empirical work shows that certain pharmaceutical TPs can retain or exceed parent toxicity, underscoring the need to derive TP-specific PNECs or apply read-across supplemented by quantitative structure–activity relationship (QSAR) estimates when data are scarce [6,56,138]. In screening and prioritization of reclaimed–water contaminants for produce safety, recent initiatives combine PEC/PNEC with occurrence data and uptake potential to select monitoring lists and refine mixture assessments [167,168,227,228].

For decision-making, HQ < 1 suggests low concern for individual substances; values ≥ 1 trigger refinement (better exposure data, chronic effect data, site-specific dilution or degradation, and consideration of bioassay-based effect screening). At mixture level, ΣHQ ≥ 1 indicates potential additive risk and prioritizes drivers for management [165,224]. Uncertainties should be documented, including representativeness of PEC/MEC, variability in treatment performance (and TP profiles), and cross-compartment transport to soils and crops [6,163,222]. Integrating PEC/PNEC with effect–based tools (e.g., algal growth inhibition, daphnid reproduction, plant bioassays) provides a robust weight–of–evidence to confirm that parent and TP risks are controlled before agricultural application.

Quantitative environmental risk assessment for contaminants originating from treated or reclaimed waters relies on the integration of exposure estimation and effect thresholds across environmental compartments. In this framework, the ratio between predicted (or measured) environmental concentrations and predicted no-effect concentrations (PEC/PNEC) provides a consistent metric for evaluating the likelihood of adverse effects. This ratio, commonly expressed as the hazard quotient (HQ), serves as a screening tool to identify substances or mixtures that may require further evaluation or mitigation. The methodology is grounded in standardized procedures adopted by the European Medicines Agency (EMA), the United States Environmental Protection Agency (USEPA), and the Organisation for Economic Co-operation and Development (OECD), and it has been adapted for complex matrices such as reclaimed wastewater, soils, and irrigation systems [153,162,165,212,221,224].

Table 13 summarizes the principal equations, assumptions, and key parameters employed in PEC and PNEC derivation and in HQ calculation for both parent pollutants and their transformation products. It outlines the hierarchy of assessment, from basic risk quotient screening to mixture-level cumulative assessment, and provides analytical expressions for exposure estimation in surface waters and soils under irrigation scenarios.

Table 13 also shows how transformation products (TPs) are integrated into the risk framework and stresses the importance of incorporating site-specific parameters, degradation rates, and bioassay validation to ensure reliable characterization of potential ecological risks associated with photocatalytically treated effluents [8,18,67,169,170,171,172,229,230,231,232,233].

### 7.3. Food Chain and Crop Safety Concerns—Accumulation in Edible Tissues

Evidence from controlled and field–proximal studies shows that a subset of pharmaceuticals and other micropollutants can enter edible plant parts after irrigation with reclaimed or treated wastewater, biosolids, or amended soils, with accumulation magnitudes governed by compound properties (pKa, logKow, ionization at rhizosphere pH), plant physiology (transpiration rate, lipid content), and growth stage [6,168,228]. Broadly, ionizable, moderately polar compounds with appreciable transpiration stream concentration factors (TSCFs) display greater leafward translocation, while strong sorption to soils or root cell walls constrains movement into harvestable tissues [168,169,228,229]. Compound-specific case studies reinforce these patterns: carbamazepine, poorly degraded and relatively mobile, accumulates in leafy vegetables, with within-plant partitioning biased toward lower leaves and measurable growth effects at higher exposure levels [173,174,234,235]. Multi-pharmaceutical exposure experiments with lettuce quantify bioconcentration factors (BCFs) and reveal that mixture composition and concentration strongly shape uptake and tissue burdens, even when single–compound behavior would predict low accumulation [160,219].

Analytical advances now support simultaneous determination of parents and key metabolites directly in edible tissues, reducing underestimation from parent-only monitoring [161,175,220,236]. Such measurements are crucial because transformation products (TPs) generated during wastewater treatment or within plant tissues can retain bioactivity and contribute to the aggregate dietary burden [228]. In risk terms, current frameworks increasingly integrate crop residue data (µg kg^−1^ fresh weight), BCFs, and consumption scenarios with PEC_plant_/PNEC_diet_ or human health margin–of–exposure calculations to prioritize substances for refined monitoring [6,168,228].

While typical concentrations in market-ready produce are often low to very low (frequently non–detect to low µg kg^−1^), exceedances occur under high–load or persistent-compound scenarios, underscoring the value of (i) treatment completeness, (ii) fit-for-purpose reuse classes, and (iii) effect-based and chemical verification prior to harvest [6,168,228]. Overall, the weight of recent evidence supports targeted, compound–specific management, focusing on persistent, mobile, bioactive APIs and their metabolites, combined with multi-residual analytics in edible tissues to secure food-chain safety where reclaimed water is used in agriculture [6,168,175,228,236]. To illustrate crop–specific variability in pharmaceutical accumulation, Table 14 compiles recent data on concentration ranges, uptake factors, and the dominant physicochemical and physiological determinants [16,45,62,86,102,174,176,235].

### 7.4. Long-Term Ecological Impacts—Persistence and Cumulative Exposure in Soils

Repeated applications of treated or reclaimed wastewater can lead to progressive soil loading by persistent parent compounds and transformation products (TPs), creating multi–seasonal exposure that differs markedly from single–dose ecotoxicity tests. Field and landscape studies show that long histories of irrigation with secondary or reclaimed effluents alter soil physicochemical properties and select for resistant microbial traits, indicating legacy effects that accumulate over 5–25 years [21,23,148,206].

Such legacy arises because many pharmaceuticals exhibit slow dissipation and appreciable sorption; so, net mass in the upper soil horizons increases when seasonal inputs exceed losses by degradation, plant uptake, leaching, and erosion. Reviews and catchment syntheses corroborate that compounds like carbamazepine (CBZ) and select antibiotics persist through conventional treatment and can persist in receiving soils, thereby acting as sentinel markers of long–term exposure [177,237].

The soil–plant system modulates this persistence through sorption–desorption dynamics, rhizosphere pH and redox conditions, and microbiome functions, which together govern whether residues remain sequestered or mobilize into porewater [172,233]. Controlled greenhouse work tracking CBZ and its metabolites demonstrates that TPs can form and persist within the soil–plant continuum, sometimes decoupling apparent parent removal from true detoxification; metabolite signals can remain in soil while plant tissue burdens fluctuate with growth stage and water regime [178,238]. In long–term reclaimed–water systems, soil microbial communities also shift: studies report changes in bacterial community composition and functional potential, with evidence for enrichment of stress-tolerant clades and resistance determinants under sustained inputs [21,23,148,206]. These microbial reorganizations may influence nutrient cycling and resilience, potentially reinforcing the persistence of certain residues via altered enzymatic capacities and carbon use efficiency.

Cumulative exposure extends beyond single compounds: multi–residual mixtures from repeated irrigation, sludge co–applications, or shallow groundwater recirculation can maintain low but chronic porewater concentrations, sustaining mixture pressures on soil biota [172,177,233,237]. Of particular concern is the co-occurrence of pharmaceuticals with metals and salts, which can co-select for resistance traits and affect biodegradation pathways, thereby indirectly enhancing chemical persistence [21,23]. Recent work tracing antibiotic resistance genes (ARGs) across irrigated systems indicates that agricultural soils can accumulate and transfer ARGs along the plant pathway (e.g., to rice tissues), implying that long-term reclaimed-water use necessitates genetic as well as chemical monitoring [179,239].

Methodologically, long-term risk characterization benefits from time–integrated monitoring (soil and porewater), multi–residual analytics for parents and TPs, and process indicators (e.g., microbial community structure, resistance markers) combined with mass-balance modeling to distinguish accumulation from steady–state conditions. Emerging frameworks link plant–level accumulation models with soil fate to quantify dietary relevance and to prioritize substances for mitigation or for polishing steps that limit TP carry-over [6]. Together, these findings underline that ecological risk under long-term reuse is shaped by persistence–input balance and by soil system feedbacks; ensuring environmental safety therefore requires verification beyond parent removal, with explicit attention to TP persistence, mixture exposures, and soil functional endpoints [6,21,23,148,172,177,178,179,180,206,233,237,238,239,240].

To support comprehensive evaluation of persistence and cumulative exposure risks under long-term wastewater reuse, a set of integrated monitoring indicators is increasingly applied to track both chemical and biological dimensions of soil system responses. These indicators encompass concentration-based measures of parent compounds and transformation products (TPs), soil accumulation profiles, microbial and enzymatic biomarkers, and resistance determinants that together provide a multi-layered picture of soil health and contaminant behavior. They also bridge laboratory-scale studies and field monitoring by combining analytical chemistry, molecular ecology, and modeling approaches to assess whether residues are accumulating, transforming, or reaching steady-state conditions.

Table 15 summarizes the principal categories of monitoring indicators, the parameters and methods commonly employed, and their interpretive roles in identifying persistence, transformation, and ecological functional shifts in soils exposed to long–term irrigation with treated or reclaimed wastewater.

## 8. Knowledge Gaps and Future Perspectives

Despite significant advances in understanding the ecotoxicological implications of emerging drug and dye pollutants, several critical knowledge gaps persist, limiting a full assessment of their long–term environmental impacts. One of the most evident limitations lies in the scarcity of soil-based phytotoxicity studies involving photocatalytically treated effluents. Most available data derive from aqueous bioassays or hydroponic tests that, while informative, do not adequately reflect the complex interactions between pollutants, soil constituents, and plant roots under field conditions. The absence of such soil–specific assessments constrains our ability to predict plant uptake, persistence, and potential risks associated with repeated irrigation using treated wastewater.

Another important gap concerns the limited understanding of soil–microbiota interactions following photocatalytic treatment. Although photocatalysis can effectively degrade parent pollutants, its influence on soil microbial communities exposed to transformation products (TPs) remains poorly characterized. Microbial assemblages play a central role in nutrient cycling, organic matter turnover, and pollutant attenuation; yet, the response of microbial taxa and functional genes to residual TPs or partial mineralization products is still insufficiently documented. Addressing this gap requires long–term microcosm and field studies integrating metagenomic and enzymatic analyses to evaluate both structural and functional shifts in soil biota.

A further challenge lies in the lack of standardized analytical methodologies for detecting and quantifying transformation products in soil matrices. While modern high–resolution mass spectrometry (HRMS) enables non-target screening, differences in sample extraction, cleanup protocols, and identification criteria hinder comparability across studies. Establishing harmonized analytical workflows and reference materials would greatly enhance reproducibility and facilitate cross–study interpretation of TP behavior, bioavailability, and toxicity.

Looking ahead, the integration of omics-based tools and predictive modeling approaches holds promise for improving mechanistic understanding and risk forecasting. Genomic, transcriptomic, and metabolomic analyses can reveal subtle stress responses in plants and microbes exposed to complex pollutant mixtures, while modeling frameworks such as PEC/PNEC and hazard quotient analyses can support the extrapolation of laboratory data to real-field scenarios. Combining these tools can yield a systems-level perspective linking molecular effects to ecological outcomes.

Finally, there is growing recognition that coupling photocatalysis with biological polishing processes, such as constructed wetlands, biofiltration, or aerobic biodegradation offers a practical route to achieve complete detoxification. Such hybrid systems capitalize on the oxidative breakdown achieved by photocatalysis and the metabolic versatility of microorganisms to degrade residual intermediates, ensuring both chemical and ecological safety.

Despite significant progress in characterizing photocatalytic transformation pathways, several important knowledge gaps remain that limit reliable environmental risk evaluation. First, the long-term implications of chronic, low-dose exposure to TPs require systematic investigation, particularly under conditions of repeated irrigation where persistent intermediates may accumulate in soil or plant tissues across successive growth cycles. The consequences of such cumulative exposure remain largely unknown. Second, the response and adaptation of soil microbial communities to recurrent TP inputs warrant deeper study; shifts in microbiome structure or function could influence nutrient cycling, soil health, and secondary pollutant transformation, yet current evidence is fragmentary. Third, the field lacks standardized and high-throughput ecotoxicological assays specifically suited for complex effluents containing TP mixtures, limiting comparability across studies and constraining regulatory assessment. Finally, there is a pressing need for integrated chemical–ecotoxicological screening frameworks that combine advanced TP characterization with plant- and soil-relevant bioassays to capture mixture-level effects and identify hazard-driving intermediates. Together, these gaps highlight key research directions necessary to develop photocatalytic processes that are both efficient and protective of terrestrial ecosystems under agricultural reuse scenarios.

Bridging these knowledge gaps will require interdisciplinary collaboration between environmental chemists, microbiologists, plant scientists, and engineers. Developing integrated experimental and modeling frameworks that encompass chemical fate, biological responses, and system-level feedbacks will be essential to ensure that photocatalytic technologies deliver truly sustainable solutions for wastewater treatment and agricultural reuse.

## 9. Conclusions

The present review provides an integrated perspective on the ecotoxicological behavior of pharmaceuticals and synthetic dyes in soil–plant systems, emphasizing the comparative effects observed before and after photocatalytic wastewater treatment. These emerging contaminants exhibit a wide spectrum of persistence and toxicity, influenced by their physicochemical characteristics, soil organic matter content, pH, redox potential, and plant uptake mechanisms. Their continuous input through treated effluents or biosolid applications contributes to the gradual enrichment of surface soils, altering enzymatic activity, nutrient cycling, and microbial community composition. Such disruptions can cascade through the plant–soil interface, affecting plant metabolism, biomass accumulation, and, ultimately, crop quality and food safety.

Photocatalytic oxidation processes, particularly those based on TiO_2_ and its modified composites, have shown high potential to degrade a broad range of persistent organic pollutants under solar or artificial irradiation. When properly optimized, these systems achieve near–complete mineralization of complex organic molecules, leading to significant reductions in phytotoxicity and soil biohazard. Nonetheless, this review highlights that incomplete or suboptimal photocatalytic treatment can yield transformation products (TPs) of intermediate toxicity, some of which remain bioactive or even more persistent than the parent compounds. Consequently, the relationship between chemical removal efficiency and biological safety is not always linear, underlining the need for toxicity-based validation alongside conventional analytical monitoring.

Comparative assessments between untreated and photocatalytically treated effluents demonstrate that detoxification generally progresses with increased irradiation time and higher degrees of mineralization. Still, the persistence of certain pollutants and their metabolites necessitates caution when reusing treated water for irrigation. Long-term exposure studies reveal that cumulative inputs can influence soil physicochemical structure and biodiversity, reinforcing the need to evaluate not only acute phytotoxicity but also chronic, sub-lethal, and mixture effects under realistic environmental conditions.

From a management standpoint, integrating photocatalytic technologies with biological post-treatments or natural attenuation strategies appears to be the most sustainable path forward. This hybrid approach can minimize the formation of stable intermediates and enhance overall detoxification. Moreover, harmonized monitoring frameworks that combine chemical, biological, and modeling tools, such as PEC/PNEC and hazard quotient analyses, are essential to ensure that reclaimed water meets safety standards for agricultural reuse and soil protection.

Future research should focus on developing scalable photocatalytic materials with improved solar-light utilization, durability, and selectivity, as well as on quantifying long-term ecological outcomes in field conditions. Expanding omics-based investigations of microbial and plant responses could also deepen the understanding of the mechanisms underlying adaptation and resilience to emerging pollutants.

Life Cycle Assessment (LCA) is essential to evaluate the environmental and economic sustainability of photocatalytic and hybrid treatment configurations, ensuring that improved effluent quality does not shift impacts to other environmental compartments.

Photocatalytic wastewater treatment represents a transformative step toward sustainable pollution control and circular water reuse. However, its full environmental benefit depends on coupling technological innovation with ecotoxicological validation, ensuring that treated waters not only meet chemical quality criteria but also safeguard the functional integrity of soil–plant ecosystems and the broader environment.

## Figures and Tables

**Table 1:** Comparative toxicity endpoints before and after advanced oxidation/photocatalytic treatment.

Target Pollutant/Matrix	Treatment Process	Bioassay and Endpoint	Toxicity Before Treatment (EC50/LC50)	Toxicity After Treatment (EC50/LC50)	Detoxification Trend
Textile secondary effluent (dyes + organics)	TiO_2_ photocatalysis (suspended catalyst)	Daphnia similis, 48 h immobilization (EC50, % effluent)	70.7% (raw effluent)	95.0% (after TiO_2_ treatment)	Toxicity decreases (higher EC50)
Textile secondary effluent	HT/Fe/TiO_2_ photocatalyst	Daphnia similis, 48 h immobilization (EC50, % effluent)	70.7%	78.6%	Moderate toxicity decrease
Pharmaceutical wastewater	N-Cu-TiO_2_/CQD photocatalysis (visible light)	Daphnia magna, acute toxicity (EC50, % effluent)	62.5%	≈150% (after photocatalysis)	Strong toxicity decrease
PAHs mixture in water	GO–TiO_2_–Sr(OH)_2_/SrCO_3_ photocatalysis	Daphnia magna, acute toxicity (EC50, ng/mL)	342.56 ng/mL	631.05 ng/mL	Toxicity decreases
Brilliant Blue FCF dye	Ozonation (200 mg O_3_/L; 50% dilution)	Daphnia magna, 48 h immobilization (EC50, mg/L)	EC50 > 100 mg/L	4.8 mg/L	Toxicity increases
Diclofenac (model solution)	Ultrasonic AOP (sonication, 240 s)	Daphnia magna, acute toxicity (EC50, mg/L)	103.4 mg/L	133.7 mg/L	Toxicity decreases
Leather wastewater	ZnO photocatalysis	Artemia salina, 24 h LC50 (% effluent)	14.9%	56.82%	Toxicity decreases
Jeans laundry textile effluent	TiO_2_ P25 photocatalysis	Artemia salina, LC50 (% effluent)	27.59%	90.86%	Toxicity decreases

**Table 2:** Key physicochemical properties influencing pollutant fate in soils and plants.

Compound/Class	Representative Use	Log Kₒw	pKa	Water Solubility (mg L^−1^)	Main Factors Influencing Fate in Soil–Plant Systems	References
Diclofenac (NSAID)	Analgesic, anti-inflammatory	4.51	4.0	2.4	Moderately hydrophobic; ionized at neutral pH; sorbs to organic matter; limited mobility; partial plant uptake	[31,37,39,80]
Carbamazepine (antiepileptic)	Psychotropic drug	2.45	13.9	17.7	Neutral at environmental pH; persistent; weak sorption; readily taken up and translocated in plants	[40,41,81,82]
Sulfamethoxazole (antibiotic)	Antimicrobial	0.9	5.6	610	Ionizable; mobile in soils; limited sorption; affects microbial activity and nitrogen cycling	[19,20,42,83]
Ciprofloxacin (antibiotic)	Fluoroquinolone	1.3	6.1	30	Strong sorption to clays; cation exchange interactions; restricted plant uptake; accumulates in roots	[31,37,43,84]
Ibuprofen (NSAID)	Analgesic	3.97	4.9	21	Weak acid; moderately hydrophobic; sorption to organic matter; partial biodegradation	[1,31,37]
Atenolol (β-blocker)	Cardiovascular agent	0.16	9.6	13,000	Hydrophilic; limited sorption; leaches easily; potential foliar absorption from irrigation sprays	[38,40,79,81]
Tartrazine (azo dye)	Food/textile colorant	2.5	10.1	1200	Anionic; mobile in soil solution; may inhibit root growth and photosynthetic enzymes	[8]
Methylene Blue (cationic dye)	Textile dye, disinfectant	0.6	—	43,600	High solubility; electrostatic adsorption on clays; strong root surface binding; photosensitizing activity	[8,10]
Reactive Black 5 (azo dye)	Textile dye	1.6	7.1	200	Hydrophilic; resistant to biodegradation; persistent in soil–water interface; limited plant uptake	[10]

**Table 3:** Influence of soil properties (pH, texture, SOM, CEC) on pollutant bioavailability: representative numeric data from the literature.

Soil Property	Condition/Range	Compound(s)	Reported Metric (Units)	Key Effect/Finding	References
Clay content & CEC	20 agricultural soils; variable clay, CEC (acidic conditions)	Ciprofloxacin	Sorption capacity: 8–141 g kg^−1^; Kd: 23–200 mL kg^−1^; Koc: 54–2146 mL g^−1^ OC; correlations: r(clay) = 0.92 *, r(CEC) = 0.64 *; pH effect r < 0.25	Sorption (and reduced mobility) increases with clay and CEC; pH had little effect in this set.	[57,97]
Soil organic carbon (SOM, OC) & speciation	Cross-study synthesis (137 papers; 106 PACs; batch & column)	Class comparison	Average Koc spans 0.0915 mL g^−1^ (anionic sulfonamides) to 84,725.5 mL g^−1^ (zwitterionic norfloxacin); sorption with OC; zwitterion > cation > neutral > anion	Higher OC and positive speciation (zwitterion/cation) strongly increase sorption (lower bioavailability/mobility).	[47,87]
Texture (coarse fine) & sorption variability	Five soils with contrasting texture/OC	Carbamazepine	Kd (measured): 1.08–14.88 L kg^−1^; literature 0.43–37 L kg^−1^	Low–moderate sorption; more mobile in sandy/low-OC soils; texture and OC drive variability.	[58,98]
Texture/OC	Five soils (as above)	Ibuprofen	Kd (measured): 0.29–20.32 L kg^−1^; literature typically 0.15–3.71 L kg^−1^	Sorption ranges widely with soil; higher OC/finer texture increases retention (reduces mobility).	[58,98]
pH/charge interactions & low sorption acids	Multi-soil comparison	Sulfameter (sulfonamide)	Literature Kd: 0.09–0.17 L kg^−1^	Weak sorption for anionic sulfonamides, higher mobility/leaching risk in many soils.	[58,98]
CEC & OC (cultivation effects)	Same 20-soil dataset; cultivated vs. uncultivated	Ciprofloxacin	In cultivated soils: r(OC, capacity) = 0.96 *; r(OC, Kd) = 0.72 *	Cultivation altered SOM quality; OC correlated strongly with sorption only in cultivated soils.	[57,97]
Dissolved organic matter (DOM) competition	Manure-DOM 0–140 mg C L^−1^	Atenolol (also sulfadiazine, caffeine)	DOM up to 140 mg C L^−1^ decreased soil sorption of atenolol (mobilizing effect)	DOM competes/complexes, increasing dissolved fraction and potential mobility.	[59,99]
Cationic dye–clay electrostatics (CEC proxy)	Raw vs. activated clay	Methylene blue (cationic dye)	Langmuir qₘ: 30–50.2 mg g^−1^; higher on activated clay	Strong electrostatic adsorption to clay surfaces; higher capacity with more reactive clay (increased CEC/area).	[60,100]
Texture–hydraulics (infiltration & leaching potential)	Sandy vs. loamy vs. clayey soils (irrigation scenarios)	General (PACs/dyes)	Higher infiltration in coarse textures	Coarse-textured soils favor percolation & leaching of weakly sorbing, soluble compounds (increased bioavailability below root zone).	[50,90]
Recycled-water irrigation (field lysimeters)	Turfgrass soils under TWW irrigation	Mixed PPCPs	Detection in leachate below root zone	Weakly sorbing PPCPs can leach under irrigation; texture/irrigation intensity modulate fluxes.	[49,89]

Notes: Kd and Koc are as reported by each study (batch/column differences may apply). Asterisks indicate statistically significant correlations (* *p* < 0.001) as reported in Table 3 cited studies.

**Table 4:** Dose–response evidence for pharmaceuticals and dyes in soil–plant systems.

Topic	Matrix/ Species	Compound(s)	Exposure Design	Key Numeric Result(s)/Finding	References
Concentration–effect patterns and thresholds	Crops & wild species (multi-species meta/SSD)	Oxytetracycline (OTC)	Multiple lab datasets aggregated; plant growth endpoints	EC10 = 0.39–26.64 mg L^−1^ (crops), 0.18–64.34 mg L^−1^ (wild); EC50 = 18.0–846.78 mg L^−1^ (crops), 46.02–2611.49 mg L^−1^ (wild).	[82,122]
Sorption controls on ECx (context)	Review/soils	Multiple classes	Synthesis of 137 studies (batch/column)	Higher sorption to clay/OM and CEC sites predicts higher ECx in shoots; weakly sorbing compounds show lower ECx in sandy/low-OC soils.	[47,87]
Hormesis/low-dose stimulation	Soil–plant (pot): sorghum	Sulfamethoxazole (SMX) ± 1% microplastics	0–50 mg kg^−1^ soil; germination and biomass endpoints	≤5 mg kg^−1^: stimulation; ≥25 mg kg^−1^: inhibition; 1% MPs reduced SMX toxicity by lowering bioavailability.	[83,123]
Hormesis (broader synthesis)	Review/plants	SMX and related antibiotics	Narrative synthesis	Low-dose stimulation, high-dose inhibition (hormetic biphasic response) across plant endpoints.	[84,124]
Time-dependent toxicity (longer exposure)	Vegetables (basil, cilantro, spinach)	SMX	Multi-week exposure vs. short-term; growth + rhizosphere	Longer exposure increased impairment and ARG abundances versus short exposure (time-dependent toxicity).	[85,125]
Transformation products matter	Rice	SMX → N^4^-acetyl-SMX	Uptake/translocation and toxicity study	N^4^-acetyl-SMX formed in situ and contributed to toxicity; transformation shifts dose–response vs. nominal SMX.	[86,126]
Dyes: acute vs. prolonged effects	Microalgae (primary producers)	Methylene blue (MB)	Acute lab tests	Concentration-dependent inhibition of growth and metabolism; strong acute effect.	[87,127]
Mixtures (plant)	Maize (soil)	Diclofenac, Ibuprofen, Ampicillin; single, binary, ternary	0–1000 mg kg^−1^ in soil; 14 days	At 1000 mg kg^−1^, Fv/F0 decreased ≈ 10–12%; mixtures produced similar or additive inhibition patterns.	[88,128]
Mixtures (aquatic plant model)	Duckweed (Lemna minor)	Diclofenac + Paracetamol	0.2–20 mg L^−1^ each, 7-day exposure	Mixture interaction ambiguous; DCF toxicity dominated; accumulation similar between single and binary exposures.	[89,129]
Modeling guidance	Review/multi-class	Pharmaceuticals and dyes	ECx modeling and risk comparison guidance	4-parameter log-logistic or Weibull for standard endpoints; Brain–Cousens for hormesis; use TWA or BMD when TPs accumulate.	[65,85,86,105,125,126]

Notes: EC_x_ = effect concentration causing x% response; ARG = antibiotic resistance genes; MPs = microplastics. According to Biczak et al. (2024), percent changes vs. control are computed from reported Fv/F_0_ means at 1000 mg kg^−1^ [128].

**Table 5:** Examples of incomplete mineralization and transformation product formation during photocatalytic degradation of pharmaceuticals and dyes.

Parent Compound	Photocatalyst/Light Source	Identified Transformation Products (TPs)	Extent of Degradation/Mineralization	Key Findings and Remarks	References
Carbamazepine	TiO_2_–g–C_3_N_4_ under simulated solar (500 W Xe lamp)	10,11-epoxycarbamazepine, acridone, oxalic acid	98% degradation, ~75% TOC removal	Rapid breakdown of parent compound but incomplete mineralization; persistent aromatic intermediates	[97,137]
Diclofenac	ZnO nanoparticles under UV–C (254 nm)	4′-hydroxydiclofenac, 5-hydroxydiclofenac, 2,6-dichloroaniline	95% degradation, ~80% TOC removal	Chlorinated intermediates resist oxidation; partial mineralization dominates	[95,135]
Sulfamethoxazole	TiO_2_ (P25) under UV–A (365 nm)	3-amino-5-methylisoxazole, sulfanilic acid, formic acid	90% degradation, ~70% TOC removal	Hydroxylation and cleavage products identified; transformation products exhibit residual toxicity	[86,126]
Methylene blue	ZnO–graphene under UV–A (365 nm)	N-demethylated derivatives, thionine, sulfate ions	99% color removal, ~80% TOC removal	Complete decolorization but persistent organic carbon; incomplete oxidation of aromatic structure	[87,127]
Malachite green	g–C_3_N_4_ nanosheets under visible light (>420 nm)	Leucomalachite green, benzophenone, benzoic acid	96% degradation, ~85% TOC removal	Formation of partially oxidized intermediates; mineralization limited by light intensity	[90,130]
Tetracycline	TiO_2_–Ag hybrid under solar light (1 sun)	Oxytetracycline, hydroxylated derivatives, carboxylic acids	94% degradation, ~78% TOC removal	Oxidative cleavage of amide and phenolic groups; slower mineralization kinetics	[62,102]

**Table 6:** Representative photocatalytic systems for the degradation of pharmaceuticals and dyes, with identified transformation products (TPs), operational conditions, and key mechanistic insights from recent studies.

Photocatalyst System	Pollutant(s)	Light Source/Conditions	Main Transformation Products (TPs) Identified	Remarks/ Key Findings	References
TiO_2_ (anatase, P25)	Sulfamethoxazole (SMX)	UV-A (365 nm), 10 mg L^−1^, 0.5 g L^−1^ catalyst	3–Amino–5–methylisoxazole, sulfanilic acid, N^4^–acetyl–SMX, short–chain carboxylic acids	Sequential hydroxylation and sulfonamide cleavage; partial mineralization	[86,126]
TiO_2_–g-C_3_N_4_ heterojunction	Carbamazepine (CBZ)	Simulated solar, 500 W Xe lamp, pH 7	10,11–Epoxy–CBZ, 2–hydroxy–CBZ, acridone, maleic acid, oxalic acid	Hydroxylation and ring-opening dominate; visible-light activation enhances degradation	[111,151]
ZnO nanoparticles	Diclofenac (DCF)	UV–C (254 nm), 15 mg L^−1^	4′–Hydroxy–DCF, 5–hydroxy–DCF, 2,6–dichloroaniline, CO_2_, H_2_O	Hydroxylation of aromatic ring and C–N bond cleavage; efficient mineralization	[95,135]
Fe–doped TiO_2_	Sulfamethazine (SMZ)	Solar irradiation (1 sun), 20 mg L^−1^	Hydroxylated SMZ derivatives, aniline, benzoquinone, SO_4_^2−^	Metal doping increased visible–light response and TP oxidation	[85,125]
TiO_2_–Ag plasmonic hybrid	Tetracycline (TC)	Solar, 10 mg L^−1^, pH 6	Oxytetracycline, hydroxylated TC, smaller carboxylic acids	Plasmonic enhancement improved light absorption and TP oxidation	[62,102]
g–C_3_N_4_ nanosheets	Malachite green (MG)	Visible light (>420 nm), 20 mg L^−1^	Leucomalachite green, 4–(dimethylamino)benzophenone, benzoic acid	N–demethylation and chromophore cleavage; complete color loss	[90,130]
ZnO–graphene composite	Methylene blue (MB)	UV–A, 365 nm, 15 mg L^−1^	Azure B, thionine, N–demethylated intermediates, sulfate ions	Stepwise demethylation and aromatic ring opening; high reusability	[87,127]
TiO_2_ immobilized on glass	Reactive Black 5 (RB5)	Real sunlight, flow reactor	Aromatic amines, naphthol, oxalic acid	Azo bond cleavage followed by aromatic ring oxidation	[31,37]
ZnO–biochar composite	Rhodamine B (RhB)	Solar simulator, 25 mg L^−1^	N,N′–diethylrhodamine, benzoic acid, CO_2_	Progressive deethylation and ring–opening; biochar improved sorption	[8]
TiO_2_–WO_3_ heterojunction	Sulfadiazine (SDZ)	Simulated sunlight, 400 W Xe lamp	Hydroxylated SDZ, sulfanilamide, small organic acids	Effective visible–light response and sustained ROS generation	[4]

**Table 7:** Representative PEC, PNEC, and Hazard Quotient (HQ) values reported for selected micropollutants and their transformation products, illustrating how risk modeling identifies hazard-driving TPs.

Compound/TP	PEC * (µg/L)	PNEC (µg/L)	HQ	Notes	References
Diclofenac (DCF)	0.05–1.0	0.05	1–20	High-risk pharmaceutical	[159,161]
4′-Hydroxydiclofenac	0.01–0.2	0.02	0.5–10	Persistent hydroxylated TP	[158]
DCF-Quinone-Imine	0.005–0.05	0.005	1–10	Strong redox cycler	[158]
2,6-Dichloroaniline	0.001–0.01	0.001	1–10	Genotoxic halogenated TP	[166]
Carbamazepine (CBZ)	0.1–1.5	0.5	0.2–3	Recalcitrant pharmaceutical	[167]
Acridone/Acridine TPs	0.005–0.05	0.005	1–10	Persistent and mutagenic	[162]
Malachite Green (MG)	0.001–0.1	0.0001	10–1000	Carcinogenic dye	[164]
Leucomalachite Green	0.001–0.05	0.00005	20–1000	Persistent and toxic TP	[168]
Benzophenone-type TPs	0.0005–0.005	0.0001	5–50	Photoreactive carbonyl TPs	[169]

* PEC ranges represent concentrations reported in wastewater effluents, downstream surface waters, or modeled environmental compartments depending on the pollutant.

**Table 8:** Fate of selected photocatalytic/biological transformation products (TPs) in soils: sorption, mobility, and biodegradability.

Transformation Product (Parent)	Soil/Matrix & Conditions	Sorption (Kd/Kf/Trend)	Mobility/ Leaching Evidence	Biodegradability/ Persistence	Notes	References
10,11–Epoxycarbamazepine (carbamazepine)	Mediterranean agricultural soils; batch sorption (single- vs. multi–solute)	Adsorption order across CBZ group: 3OH-CBZ > CBZ > EPCBZ > 10OH-CBZ; adsorption decreases in multi–solute (competition)	Lower adsorption in sandy/low-OM soils implies higher leaching potential vs. CBZ	Reported as TP in soil systems (incomplete removal)	Texture (silt/clay) strengthened adsorption; OM less dominant under competitive conditions	[114,177]
10,11–Epoxycarbamazepine (carbamazepine)	Loamy sand; soil–water contact kinetics (100 µg L^−1^)	Sorption contributes early (≤48 h) but is modest for CBZ group	EPCBZ detected as TP; indicates mobility from biotic transformation during soil contact	First–order removal observed overall; EPCBZ appears as biotransformation product (incomplete mineralization)	Microbial adaptation reduced half-lives for some compounds; CBZ removal limited	[115,178]
N^4^–Acetyl–sulfamethoxazole (Ac-SMX) (sulfamethoxazole)	Loamy sand; soil–water contact kinetics	SMX shows fast initial sorption; Ac–SMX reported as TP (sorption not separately quantified)	Presence of Ac–SMX indicates potential to move with pore water post–formation	Microbial adaptation reduces apparent half-lives; Ac–SMX appears as TP (incomplete degradation)	Highlights that TPs form during infiltration/contact and can persist transiently	[99,139]
Sulfonamide TPs/sulfanilic-type fragments	Five agricultural soils; soil & soil-manure mixes; batch & column	Manure increased Kd of sulfonamides (up to 5.87× for sulfadiazine, 2.49× for SMX); pH & OC were key drivers	Under high simulated rainfall (180 mm), sulfonamides showed high migration potential in low–OC/high-pH soils	Not TP-specific half-lives; points to higher mobility of more polar species	Manure can immobilize near surface yet does not eliminate leaching risk under intense percolation	[116,179]
Aromatic amines (azo dye reduction/photolysis TPs; e.g., aniline derivatives)	Soils/sediments; mechanistic sorption overview	Sorption via H-bonding, π–π with humics; also irreversible binding/aging pathways	Mobility increases with DOM complexation; risk of subsurface transport when sorption sites are saturated	Some amines persistent; can undergo oxidative coupling to more recalcitrant products	Key class of azo–dye TPs with documented mutagenicity concerns	[117,180]
Aromatic amines (azo dye TPs)	Photocatalytic dye studies feeding into soil exposure	— (formation stage)	—	Aromatic amines identified as intermediates during azo-dye degradation under light; can persist post-discharge	Confirms environmental relevance of amine TPs entering soils via reuse	[118,181]
General sulfonamide TPs (incl. de-sulfonated, hydroxylated forms)	Multi–soil datasets, model analysis	Kd/Kf negatively correlated with pH for SMX; SOM/CEC increase sorption	Higher pH → greater mobility; effects modulated by soil OC	Increased polarity → often more biodegradable, but compound-dependent	Mechanistic evidence for pH–dependent sorption of ionizable TPs	[116,179]
Malachite green → leucomalachite green (LMG) (dye TP)	Aquatic → soil relevance; microbial degradation studies	— (soil sorption not quantified here)	Potential mobility as reduced, more lipophilic LMG varies by matrix	Half-lives of MG in microbial systems on the order of ~2–4 days (strain-dependent); LMG persistence known in biota	Demonstrates dye TP formation and variable persistence; informs soil exposure via reuse	[119,182]

Notes: Kd (distribution coefficient) and Kf (Freundlich sorption constant) are expressed in L kg^−1^ where available. Higher Kd or Kf values indicate stronger sorption and lower mobility. Mobility and persistence trends were derived from batch and column studies and supported by field leaching evidence. OM: organic matter; DOM: dissolved organic matter; SMX: sulfamethoxazole; CBZ: carbamazepine; EPCBZ: 10,11-epoxycarbamazepine. Arrows and the “—” symbol are used to convey qualitative trends and data availability in a concise and transparent manner. Arrow (→) indicate directional trends in sorption, mobility, or biodegradability derived from comparative or multi-condition studies, where quantitative values (Kd, Kf, half-lives) were reported as ranges, relative changes, or correlations rather than as single fixed parameters. The “—” symbol denotes cases where a specific process (e.g., soil sorption or mobility) was not directly quantified or not the primary focus of the cited study, rather than indicating absence of the process.

**Table 9:** Comparative characteristics, examples, and effects of parent compounds and photocatalytically degraded transformation products (TPs) in soil–plant systems.

Feature	Parent Compounds (Examples)	Photocatalytically Degraded Compounds (TPs) (Examples)
Chemical structure	Stable aromatic or heterocyclic molecules such as carbamazepine, diclofenac, sulfamethoxazole, and dyes like methylene blue, malachite green	Hydroxylated or carboxylated derivatives such as 10,11–epoxycarbamazepine, 4′–hydroxydiclofenac, sulfanilic acid, or leucomalachite green
Polarity and solubility	Generally low polarity and solubility; e.g., carbamazepine (log Kₒw = 2.45) and methylene blue (log Kₒw = 1.2)	Increased polarity and solubility; e.g., hydroxylated or ring–opened intermediates (log Kₒw < 1) exhibit greater mobility in water
Sorption to soil	Strong sorption to organic matter and clay (Kd ≈ 20–100 L kg^−1^ for diclofenac)	Weak sorption (Kd < 5 L kg^−1^ for sulfanilic acid); higher leaching and bioavailability potential
Persistence	High stability; e.g., carbamazepine half-life > 120 days in soil	Reduced persistence (t_1_/_2_ ≈ 10–30 days), but some intermediates (e.g., benzoquinones, aromatic amines) remain reactive and stable
Bioavailability	Limited due to hydrophobicity and strong adsorption	Enhanced uptake due to smaller size and higher solubility; detected in plant tissues after exposure to treated effluents
Typical transformation pathways	—	Hydroxylation, demethylation, nitration, ring cleavage, or oxidation to quinones and low-molecular-weight acids
Plant responses	Moderate inhibition of germination and photosynthesis; e.g., reduced chlorophyll in lettuce and maize under diclofenac or methylene blue exposure	Enhanced oxidative stress, chlorophyll loss, membrane lipid peroxidation, and DNA damage; e.g., ROS accumulation after exposure to sulfanilic acid or 4′-hydroxydiclofenac
Microbial effects	Inhibition of soil enzyme activity (dehydrogenase, urease, phosphatase); reduced microbial biomass under antibiotic exposure	Community shifts toward resistant taxa (e.g., *Pseudomonas*, *Bacillus*); inhibition of nitrifying and denitrifying bacteria
Soil fauna responses	Sublethal effects on *Eisenia fetida* (growth inhibition, behavioral changes)	Oxidative stress and DNA damage from reactive aromatic amines and quinone intermediates
Ecotoxicological implication	Long–term persistence, chronic low–level toxicity, potential bioaccumulation	Acute oxidative or genotoxic effects; reduced persistence but increased biological reactivity
Differentiated effects in soil and plants	In soil: reduced microbial activity, enzymatic inhibition, and nitrogen cycle disruption. In plants: mild toxicity, reduced biomass, pigment alteration, and moderate ROS generation.	In soil: strong oxidative stress, increased antibiotic resistance genes, and microbial imbalance. In plants: pronounced oxidative stress, pigment degradation, enzymatic inhibition, and DNA fragmentation indicating genotoxicity.

**Table 10:** Comparative summary of representative studies evaluating phytotoxicity before and after photocatalytic treatment of effluents.

Reference	Pollutant Type/Matrix	Photocatalytic System	Plant or Bioassay	Distinct Findings (Beyond Narrative)	Ecotoxicological Outcome
[132,193]	Real textile effluent (dyes, surfactants, auxiliaries)	TiO_2_ and TiO_2_/H_2_O_2_ under UV (up to 6 h)	*Lactuca sativa* (germination, root growth)	Identified intermediate aromatic acids and peroxides responsible for early toxicity spikes; full TOC reduction > 70% restored normal germination.	Transient ↑ toxicity → full detoxification with extended irradiation
[134,195]	Pharmaceutical mixture (atenolol, chlorpromazine, metronidazole)	TiO_2_ (UV 365 nm, 8–16 h)	*Spirodela polyrrhiza* (growth rate, chlorophyll a content)	Demonstrated correlation between dissolved organic carbon (DOC) decrease and frond regrowth; photosynthetic recovery lagged 2–3 h behind DOC removal.	Progressive ↓ toxicity proportional to DOC/mineralization
[55,135]	Real industrial & municipal effluents	TiO_2_, ZnO, g-C_3_N_4_ (various reactors)	Review of multi-species assays	Highlighted disparity between > 90% chemical removal and < 50% toxicity reduction; proposed multi-endpoint testing to detect latent TPs.	Variable detoxification depending on TP persistence
[136,196]	Natural stream water with contaminants	Solar TiO_2_ photocatalysis	*Lactuca sativa* (root elongation)	Quantified residual hydrogen peroxide accumulation affecting seed viability during mid–treatment; complete mineralization eliminated effect.	↓ toxicity after complete mineralization
[137,197]	Pharmaceutical-laden secondary effluent	Coupled bio-solar photocatalysis	Mixed aquatic plant assays	Noted synergy between biological pre-treatment and photocatalysis lowering TP formation rate; toxicity curve fitted to logistic decay model (R^2^ > 0.9).	Gradual detoxification with hybrid process
[56,138]	Sulfonamide antibiotics (lab scale)	TiO_2_ UV + aerobic biodegradation	Microbial/mechanistic analysis	Identified sulfone/nitro-intermediates persisting post–photolysis; complete mineralization after bio-polishing confirmed by TOC < 5%.	Effective detoxification only after combined process

Legend: ↑ = increase; ↓ = decrease; TOC = total organic carbon; DOC = dissolved organic carbon. Arrows (↑, →, ↓) denote qualitative directional trends (increase or decrease) in parameters such as sorption strength, mobility, persistence, or biodegradability, as inferred from comparative analyses, correlations, or multi-condition experiments reported in the cited studies. The “—” symbol indicates that a given parameter was not explicitly measured, not reported, or not the primary focus of the referenced study. This notation does not imply absence of the process, but rather reflects data unavailability or study scope limitations.

**Table 11:** Quantitative summary of phytotoxicity change following photocatalytic treatment of different effluents.

Reference	Pollutant/ Matrix	Photocatalytic System	Plant Bioassay	% TOC/DOC Removal	% Change in Phytotoxicity	Direction of Change
[132,193]	Textile effluent (dyes, surfactants)	TiO_2_ and TiO_2_/H_2_O_2_ (UV, 6 h)	Lactuca sativa (root elongation)	≈65–80% TOC removal	≈70% toxicity reduction after 6 h; transient + 25% inhibition after 2 h	↓ after complete treatment; transient ↑ early stage
[134,195]	Pharmaceutical mixture (atenolol, chlorpromazine, metronidazole)	TiO_2_ (UV 365 nm, 8–16 h)	Spirodela polyrrhiza (growth, frond number)	≈90% DOC removal after 16 h	≈100% detoxification; initial 90% inhibition reversed after 16 h	↓ toxicity with time/mineralization
[55,135]	Municipal/industrial real effluents	Various photocatalysts (TiO_2_, ZnO, g–C_3_N_4_)	Multiple plant species (reviewed data)	40–90% pollutant removal (average)	40–85% reduction in toxicity (varies by effluent type)	↓ moderate to high reduction; variable persistence
[136,196]	Contaminated stream water	Solar TiO_2_ (optimized vs. incomplete)	*Lactuca sativa* (root elongation)	≈80–95% TOC removal (complete mineralization)	≈50–100% toxicity reduction; 2× root length vs. untreated	↓ significant reduction after full mineralization
[137,197]	Pharmaceutical–laden secondary effluent	Coupled biological + solar photocatalysis	Mixed aquatic plants (growth assays)	≥85% organic carbon removal	60–90% decrease in toxicity across treatment stages	↓ gradual detoxification
[56,138]	Sulfonamides (sulfamethoxazole, sulfadiazine)	TiO_2_ (UV) + aerobic biodegradation	Mechanistic/microbial endpoints	≈95% TOC removal (after bio–polishing)	20–40% higher toxicity during partial oxidation; full detox after biodegradation	↑ transient toxicity → ↓ final detoxification

Legend: ↑ = increase; ↓ = decrease; TOC = total organic carbon; DOC = dissolved organic carbon. Arrows (↑, ↓) are used to indicate the direction of change in phytotoxicity following photocatalytic treatment, based on quantitative comparisons of biological endpoints before and after treatment. Combined notations (e.g., ↑ → ↓) are used where studies explicitly report non-linear toxicity evolution, with initial toxicity enhancement followed by detoxification upon extended treatment or higher mineralization levels.

**Table 12:** Comparative overview of international standards and guidelines for the safe reuse of treated wastewater in agriculture.

Focus	Main Parameters/Limits	Application Scope	References
Microbial safety for irrigation water (Classes A–D)	*E. coli* ≤ 10–10,000 cfu/100 mL; BOD_5_ ≤ 10 mg L^−1^; TSS ≤ 10 mg L^−1^; Turbidity ≤ 5 NTU; Legionella ≤ 1000 cfu/L (aerosol risk)	Crop irrigation, aquifer recharge	[147,149,207,208]
Health–based targets for reuse	*E. coli* reduction ≥ 6–7 log units; Helminth eggs ≤ 1/L	Global risk–based approach	[150,209]
Risk–based water reuse guidance	*E. coli*, enteric viruses, helminths; turbidity and residual chlorine control	Crop and landscape irrigation	[37,47,151,210]
Design and operation of reuse systems	Fit–for–purpose quality; microbiological and chemical validation	Design standards for irrigation reuse	[152,211]
Agronomic and soil protection criteria	EC < 3 dS/m; SAR < 9; Cl^−^ < 140 mg/L; B < 0.7 mg/L	Crop productivity and soil structure	[153,212]
Soil protection from heavy metals	Cd ≤ 3 mg/kg; Pb ≤ 750 mg/kg; Zn ≤ 2500 mg/kg; Cu ≤ 1000 mg/kg	Sludge reuse in agriculture	[154,213]

Note: EC—electrical conductivity; SAR—sodium adsorption ratio; NTU—nephelometric turbidity unit.

**Table 13:** Summary of equations and parameters for PEC, PNEC, and hazard quotient (HQ) derivation for parent compounds and transformation products.

Component	Equation/Description	Key Parameters and Notes
Risk metric	HQ = PEC/PNEC ΣHQ = Σᵢ (PECᵢ/PNECᵢ)	HQ < 1 = low concern; HQ ≥ 1 = refinement required (improved data, site specifics, or bioassays).
Pnec derivation	PNEC = NOEC/AF	AF: 10 (≥3 chronic taxa); 50–100 (1–2 chronic taxa); 1000 (acute data only). Use read-across or QSAR for TPs with high AF (≥1000).
PECsurface water	PECsw = Ceff/DF or (Ceff × Qeff)/(Qriver + Qeff)	Ceff = effluent concentration (µg L^−1^); DF = dilution factor (5–20); Qeff, Qriver = flow rates. Use 90th percentile MECs for screening.
PECsoil (Irrigation)	PECsoil,init ≈ [Cw × I]/[zmix × ρb] PECsoil,TWA ≈ [Cw × I × N]/[zmix × ρb] × [1 − e^−^ᵏᵀ]/(kT)	Cw = water concentration (µg L^−1^); I = irrigation (1 mm = 1 L m^−2^); zmix = 0.05–0.20 m; ρb = 1.3–1.6 kg L^−1^; k = ln2/DT50.
Crop Exposure (Soil → Plant)	PECplant ≈ UF × Cpw	Cpw = porewater conc. (µg L^−1^) ≈ PECsoil/Kd; UF = uptake factor (e.g., TSCF for leafy crops).
Transformation Products (TPs)	HQTP = PECTP/PNECTP	Derive PNECTP from QSAR or read-across using AF ≥ 1000; include in ΣHQ to capture mixture risk.
Uncertainty and Verification	—	Evaluate Ceff/MEC representativeness, DF, soil depth, TP formation; pair PEC/PNEC with effect-based bioassays for confirmation.

**Table 14:** Reported accumulation ranges of pharmaceuticals in edible crops and key drivers of uptake.

Crop Type	Reported Concentration Range in Edible Tissue ^1^	Typical BCF/TSCF or Uptake Factor	Key Physicochemical or Crop Drivers
Leafy vegetables (e.g., *Lactuca sativa*, spinach)	Up to ~0.1–5 µg kg^−1^ fresh weight; frequently low ng kg^−1^ for many compounds; higher for persistent APIs such as carbamazepine and diclofenac [16,175,236]	BCF ≈ 0.1–1 (for moderately mobile compounds)	High transpiration and direct foliar contact; weak ionisation favours xylem transport.
Fruiting crops (e.g., tomato, cucumber)	Typically tens–hundreds ng kg^−1^; some persistent compounds (e.g., carbamazepine) up to ~0.2–0.5 µg kg^−1^ under repeated irrigation [16,173,234].	TSCF or UF~0.01–0.1	Lower transpiration, fruit cuticle barrier, longer growth cycle limits systemic translocation.
Root/tuber crops (e.g., carrot, potato)	Usually <100 ng kg^−1^; <50 ng kg^−1^ for most APIs under field conditions [45,86,175,236]	BCF~0.01–0.1	Direct contact with soil; strong sorption and vacuolar sequestration reduce upward movement.
Grain/cereal crops (e.g., wheat, maize)	Often a few ng kg^−1^ to non–detect levels for most pharmaceuticals [6,174,235]	BCF < 0.01	Low transpiration rate and limited xylem transport to reproductive tissues.

^1^ Ranges approximate, derived from recent greenhouse and field studies; actual values depend on compound properties, irrigation history, soil type, and crop physiology.

**Table 15:** Key monitoring indicators and metrics for assessing long–term persistence and cumulative exposure of pharmaceuticals and transformation products (TPs) in soils under treated wastewater reuse.

Indicator Category	Parameter/Metric	Analytical or Methodological Approach	Purpose/ Interpretation	References
Chemical persistence	Time–weighted mean concentrations of parent compounds and TPs in soil porewater (ng L^−1^–µg L^−1^ range)	Passive samplers (PES), periodic LC–MS/MS campaigns	Assess temporal accumulation and potential for groundwater migration	[172,177,233,237]
Soil accumulation	Residue mass balance (mg kg^−1^ soil), depth profiles	Soil core sampling, solvent extraction + LC–MS/MS	Quantify vertical persistence and sorption to upper horizons	[148,178,206,238]
Transformation product tracking	Parent/TP ratio and metabolite identification	Target/non–target HRMS, suspect screening	Evaluate incomplete mineralization or secondary formation of TPs	[172,177,233,237]
Microbial community integrity	Shannon diversity index, functional gene abundance	16S rRNA and metagenomic sequencing	Detect structural or functional shifts due to chronic exposure	[21,23,148,206]
Resistance indicators	Abundance of antibiotic resistance genes (ARGs), mobile genetic elements	qPCR, metagenomics, plasmid profiling	Assess co-selection effects under wastewater–derived pressure	[179,239]
Soil enzyme activity	Dehydrogenase, phosphatase, urease activities (µmol product g^−1^ h^−1^)	Colorimetric enzymatic assays	Evaluate microbial metabolic capacity and nutrient cycling disruption	[21,23,172,233]
Model–based exposure	Modeled PEC_soil, half-life (DT_50_), cumulative hazard index (HQsum)	Multimedia fate or crop-soil coupling models (e.g., SimpleBox, Trapp framework)	Quantify persistence–input balance and risk thresholds over time	[6,179,180,239,240]

## Data Availability

No new data were created or analyzed in this study.
